# Aromatic Glucosinolate Biosynthesis Pathway in *Barbarea vulgaris* and its Response to *Plutella xylostella* Infestation

**DOI:** 10.3389/fpls.2016.00083

**Published:** 2016-02-08

**Authors:** Tongjin Liu, Xiaohui Zhang, Haohui Yang, Niels Agerbirk, Yang Qiu, Haiping Wang, Di Shen, Jiangping Song, Xixiang Li

**Affiliations:** ^1^Key Laboratory of Biology and Genetic Improvement of Horticultural Crops, Institute of Vegetables and Flowers, Chinese Academy of Agricultural Sciences, Ministry of AgricultureBeijing, China; ^2^Copenhagen Plant Science Center and Plant Biochemistry Laboratory, Department of Plant and Environmental Sciences, University of CopenhagenFrederiksberg, Denmark

**Keywords:** *Barbarea vulgaris*, diamondback moth, glucosinolate, gene expression profile, induced defenses, plant-herbivore interaction, side chain modification

## Abstract

The inducibility of the glucosinolate resistance mechanism is an energy-saving strategy for plants, but whether induction would still be triggered by glucosinolate-tolerant *Plutella xylostella* (diamondback moth, DBM) after a plant had evolved a new resistance mechanism (e.g., saponins in *Barbara vulgaris*) was unknown. In *B. vulgaris*, aromatic glucosinolates derived from homo-phenylalanine are the dominant glucosinolates, but their biosynthesis pathway was unclear. In this study, we used G-type (pest-resistant) and P-type (pest-susceptible) *B. vulgaris* to compare glucosinolate levels and the expression profiles of their biosynthesis genes before and after infestation by DBM larvae. Two different stereoisomers of hydroxylated aromatic glucosinolates are dominant in G- and P-type *B. vulgaris*, respectively, and are induced by DBM. The transcripts of genes in the glucosinolate biosynthesis pathway and their corresponding transcription factors were identified from an Illumina dataset of G- and P-type *B. vulgaris*. Many genes involved or potentially involved in glucosinolate biosynthesis were induced in both plant types. The expression patterns of six DBM induced genes were validated by quantitative PCR (qPCR), while six long-fragment genes were validated by molecular cloning. The core structure biosynthetic genes showed high sequence similarities between the two genotypes. In contrast, the sequence identity of two apparent side chain modification genes, the *SHO* gene in the G-type and the *RHO* in P-type plants, showed only 77.50% identity in coding DNA sequences and 65.48% identity in deduced amino acid sequences. The homology to *GS-OH* in *Arabidopsis*, DBM induction of the transcript and a series of qPCR and glucosinolate analyses of G-type, P-type and F_1_ plants indicated that these genes control the production of *S* and *R* isomers of 2-hydroxy-2-phenylethyl glucosinolate. These glucosinolates were significantly induced by *P. xylostella* larvae in both the susceptiple P-type and the resistant G-type, even though saponins are the main DBM-resistance causing metabolites in G-type plants. Indol-3-ylmethylglucosinolate was induced in the G-type only. These data will aid our understanding of the biosynthesis and induction of aromatic glucosinolates at the molecular level and also increase our knowledge of the complex mechanisms underpinning defense induction in plants.

## Introduction

Plants have evolved constitutive and inducible resistance against herbivores, which compete for the same resources in the plant (Rasmann et al., 2015). The classic theory presumed that inducible defenses is a cost-saving strategy, because resources can divert from defense to growth under suitable growth conditions (Rasmann et al., 2015). Hence, induction of defenses is potentially advantageous in crops, where resources allocated to defense should be minimized.

*Barbarea vulgaris* is a wild crucifer, growing in temperate regions (Badenes-Pérez et al., 2010; Toneatto et al., 2010). It is a model plant for studying saponin and glucosinolate biosynthesis, insect resistance and plant-insect co-evolution (Kuzina et al., 2011). A long term goal of this research is identification of genes, metabolites and regulatory mechanisms that could confer resistance traits to cultivated crucifers. In addition, this research aims at a deeper understanding of insect counter-resistance development. There are two morphologically distinct types of *B. vulgaris*: G-type and P-type, which are named from their glabrous and pubescent leaves, respectively (Nielsen, 1997; Christensen et al., 2014). These types also have contrasting resistance phenotypes and secondary metabolite profiles (Dalby-Brown et al., 2011). The G-type is strongly resistant to some crucifer-specific insect species, including the diamondback moth (DBM, *Plutella xylostella*) and some kinds of flea beetles (*Phyllotreta nemorum*), while the P-type is completely susceptible to them. The G-type DBM and flea beetle resistance is attributed to biosynthesis of triterpenoid saponins, a unique feature among crucifers (Nielsen, 1997; Shinoda et al., 2002; Kuzina et al., 2009, 2011; Dalby-Brown et al., 2011; Khakimov et al., 2015; Liu et al., 2015a; Zhang et al., 2015). P- and G-type *B. vulgaris* also differ in the type and content of glucosinolates, which are secondary metabolites known as effecters in plant defenses against other insects and some diseases.

**Figure 1:**
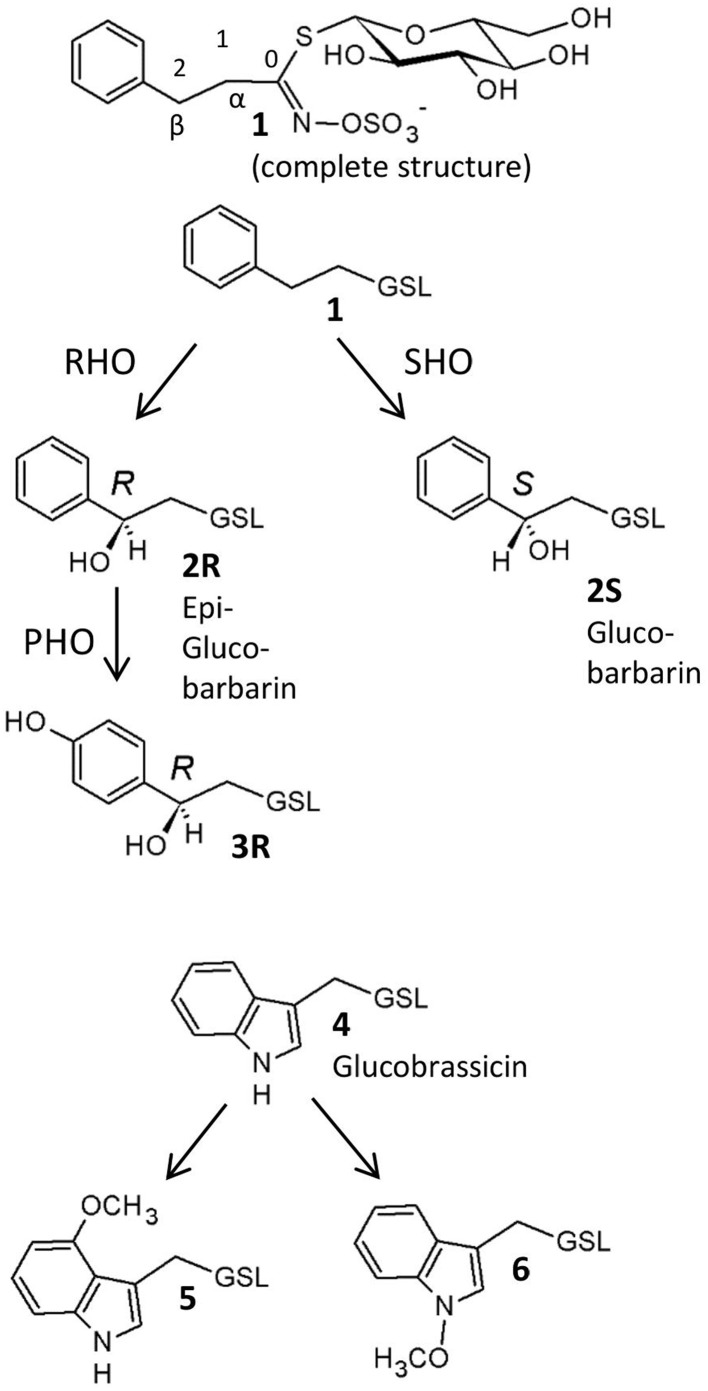
**Glucosinolates in *Barbarea vulgaris* leaves as detected in this investigation, with suggested biosynthetic relationships**. **1**, 2-phenylethyl GSL (gluconasturtiin); **2R**, (2*R*)-2-hydroxy-2-phenylethyl GSL (epiglucobarbarin); **2S**, (2*S*)-2-hydroxy-2-phenylethyl GSL (glucobarbarin); **3R**, (2*R*)-2-hydroxy-2-(4-hydroxyphenyl)ethyl GSL (4-hydroxyepiglucobarbarin); **4**, 3-indolylmethyl GSL (glucobrassicin); **5**, 4-methoxy-3-indolylmethyl GSL (4-methoxyglucobrassicin); **6**, *N*-methoxy-3-indolylmethyl GSL (neoglucobrassicin). GSL, glucosinolate; RHO, *R*-hydroxylation; SHO, *S*-hydroxylation, PHO, *para*-hydroxylation. In all structures except the upper complete structure, the constant glucosinolate backbone is indicated GSL.

Glucosinolates (Figure 1) are thioglucosides derived from amino acids and are the distinctive secondary metabolites in crucifers (Agerbirk and Olsen, 2012). They provide an activated defense system because their hydrolysis catalyzed by endogenous thioglucosidases, also known as myrosinases (E.C. 3.2.1.147), produce toxic isothiocyanates and other products (Kuchernig et al., 2012) as well as signal molecules important for resistance against microbes (Bednarek et al., 2009; Clay et al., 2009). In contrast to antimicrobial phytoalexins, which are only biosynthesized upon induction (Pedras et al., 2015), glucosinolates are classified as phytoanticipins because they are pre-formed defenses. However, additional induction of some biosynthetic groups of glucosinolates, in particular the tryptophan derived indole glucosinolates, is well-known (Bodnaryk, 1992; Hopkins et al., 1998; Bartlet et al., 1999). In this way, the glucosinolate-myrosinase defense system is available immediately upon tissue damage, while supplementary induction serves to allocate additional resources only when needed.

The G-type of *B. vulgaris* is resistant to DBM larvae due to its saponin content, while DBM larvae are known to be insensitive to glucosinolates (Ratzka et al., 2002). The major glucosinolates in both types are phenethyl glucosinolates with a β-hydroxy group (Figure 1), which are hydrolyzed to non-isothiocyanate metabolites (oxazolidine-2-thiones and thiazolidine-2-ones). These products were recently suggested to be intermediates in phytoalexin biosynthesis in *B. vulgaris* (Agerbirk and Olsen, 2015; Pedras et al., 2015). In addition to the defensive function, glucosinolates also stimulate feeding and oviposition of many glucosinolate-adapted insects such as the DBM. Indeed, gravid DBM prefer oviposition on *B. vulgaris* because of the glucosinolate content (Badenes-Pérez et al., 2011); however, no larvae could survive on G-type plants. Thus, this plant could be used as a “dead-end” trap crop (Lu et al., 2004; Badenes-Pérez et al., 2005). A quantitative increase of glucosinolates in *B. vulgaris* was accomplished by sulfur fertilization and found to improve the effectiveness of the insect trap (Badenes-Pérez et al., 2010, 2011). For all of these reasons, it was interesting to test the induction of glucosinolates and compare their magnitude in both types of *B. vulgaris*.

In contrast to the dominance of methionine derived glucosinolates in *Arabidopsis thaliana* (Gigolashvili et al., 2008), the major glucosinolates in *B. vulgaris* are derived from homo-phenylalanine, and their biosynthesis is largely unknown. Collectively, these glucosinolates are best named phenethyl glucosinolates. A number of genetic variants contain various phenethyl glucosinolates and yield different hydrolysis products upon damage (Agerbirk et al., 2014; Agerbirk and Olsen, 2015), conferring differential effects on insect herbivores (van Leur et al., 2008). The G- and P- type *B. vulgaris* differ in their content of stereochemical isomers (diastereomers with respect to hydroxyl groups and hence termed epimers) of 2-hydroxy-2-phenylethylglucosinolate (Figure 1; Kuzina et al., 2011). The G-type mainly contains the (2*S*)-epimer (glucobarbarin, **2S**) and the P-type mainly contains the (2*R*)-epimer (epiglucobarbarin, **2R**), which are both assumed to be biosynthesized by hydroxylation of a common precursor, 2-phenylethylglucosinolate (**1**) (Kuzina et al., 2011). Two previous reports have investigated the genetics of this hydroxylation. In the first report, the gene coding for biosynthesis of **2S** was found to be a single dominant gene, while a rare phenotype dominated by **1**, devoid of a hydroxyl group, was controlled by a recessive allele of the same locus (van Leur et al., 2006). In the second report, quantitative trait locus mapping was applied to identify the genes involved in G- and P-type *B. vulgaris* glucosinolate polymorphism using a P × G-type derived F_2_ population (Kuzina et al., 2011). The genes determining the **2S**/**2R** difference between G-type and P-type were mapped on two chromosome regions spanning 20–60 cM (Kuzina et al., 2011). Also in this study, recombinant plants dominated by **1** rather than **2R** or **2S** were reported, in accordance with separate loci for biosynthesis of **2R** and **2S**, respectively, from **1**.

Despite these pioneering genetic investigations, the glucosinolate biosynthesis pathway has not been compared in molecular detail between the two plant types, neither has their DBM-feeding-responses been studied. RNA-seq allows simultaneous acquisition of sequences for gene discovery, as well as transcript identification involved in specific biological processes (Wang et al., 2013). In recent papers, using Illumina paired-end sequencing, we reported the transcriptome profile of G-type *B. vulgaris* at 0, 1, 4, 8, 12, 24, and 48 h (Wei et al., 2013) and P-type at 0 and 4 h after infestation by DBM (Zhang et al., 2015). These data offered sufficient information to study glucosinolate synthesis related genes and their regulation in different types of *B. vulgaris* in response to DBM.

In the present work, we evaluated the impact of constitutive and DBM-induced changes in glucosinolates of *B. vulgaris*, using G-type and P-type plants, which contain resistant and non-resistant saponins, respectively. Consequently, we identified genes that were likely to be involved in phenethyl glucosinolate biosynthesis in G- and P-type *B. vulgaris*, based on RNA-seq data by comparison with the known pathway of methionine derived glucosinolates in *A. thaliana* (Wittstock and Halkier, 2002). We then characterized the gene expression patterns in response to DBM. The sequences and expression of some genes in the pathway were additionally validated by molecular cloning and quantitative PCR (qPCR). Two genes controlling the *S* and *R* epimers of glucosinolates in the two genotypes were identified. These data revealed a correlation between glucosinolate production and gene expression, and provide a better understanding of the defensive strategy of DBM resistant and susceptible *B. vulgaris* in terms of glucosinolates. At the same time, these results will deepen our understanding of the biosynthesis of phenethyl glucosinolates at the molecular level.

## Materials and methods

### Plant material and cultivation

Seeds of *B. vulgaris* accessions B4 (P-type) and B44 (G-type) were obtained from the University of Copenhagen, Denmark (Agerbirk and Olsen, 2015). Accession B4 is identical to accession NGB23547 publicly available at www.nordgen.org. The F_1_ hybrid was generated by a G-type (male) × P-type (female) cross in the Institute of Vegetables and Flowers, Chinese Academy of Agricultural Sciences, China. Plant growth conditions were the same as in previous pyrosequencing studies (Wei et al., 2013; Zhang et al., 2015). *Barbarea vulgaris* seeds were surfaced-sterilized in 1% NaClO and sown into 10 × 10 cm pots filled with a mixture of peat soil. Plants were kept in a growth chamber at 25°C/20°C (light/dark), and 60% relative humidity, at 225 μmol·m^−2^·s^−1^ light intensity, and on a 16 h:8 h (light:dark) photoperiod. Plants were watered as needed and fertilized with half-strength Hoagland's nutrient solution. Plants at 10 weeks old were used in this study.

### Insect feeding treatment

Diamondback moth larvae were originally obtained from a cabbage field in Taigu, Shanxi, China in the autumn of 2010 and reared on cabbage at 25°C, 12 h:12 h photoperiod and 60% relative humidity. Three DBM third-instar larvae were inoculated on each fully extended leaf of the 10 weeks old *B. vulgaris* plants from time zero until the time of sampling (8 or 48 h); seven leaves per plant were inoculated. The transcriptome results showed that most genes were significantly induced after 4 h of infestation by DBM; therefore, this time point was set for qPCR. To obtain optimal glucosinolate induction, 8 and 48 h of infestation by DBM were used for glucosinolate analysis. Plants with DBM and DBM-free control plants were kept in a growth chamber under the same condition. After 48 h, the leaves of control plants (not including the petiole) were cut and immediately flash frozen in liquid nitrogen. For DBM-treated plants, the DBM larvae were removed with a brush and the leaves were harvested using the same method used to harvest the control. Five plants were used as biological replicates. All material was stored at −80°C.

### Glucosinolate extraction and analysis

Glucosinolates were extracted according to the method of La et al. (2009). Two hundred milligrams of freeze-dried leaf powder was weighed in a 15 mL tube and 5 mL of boiling 100% methanol was added. Glucotropaeolin (benzylglucosinolate) was added as an internal standard. Samples were then incubated at 80°C for 15 min in a water bath. The mixture was centrifuged at 7000 × g for 10 min at 4°C and the supernatant was decanted into another tube. The extraction was repeated twice from residues using the same procedure with 70% methanol (v/v). The three supernatants were combined and 2 mL of each glucosinolate extract was added to a mini-column filled with diethylaminoethanol (DEAE) Sephadex A-25 (Amersham Bio-sciences, Uppsala, Sweden) activated with 0.02 M NaAc (Sinopharm Chemical Reagent, Beijing, China), and desulfated by sulfatase (Dikma Technologies, CA, USA). After reaction at room temperature overnight (16 h), the desulfated glucosinolates were eluted with 2 mL de-ionized water and stored at −20°C before to high performance liquid chromatography (HPLC) analysis.

Samples were analyzed by HPLC on an Agilent HP 1100 Series instrument equipped with a C-18 reversed-phase column (Nova-PakR, 3.9 × 150 mm, 5 μm particle size) using 0.5 g·L^−1^ ammonium acetate (solvent A)–a mixture of 1 L 0.1 mol·L^−1^ ammonium acetate and 300 mL methanol (solvent B) gradient at a flow rate of 1 mL·min^−1^ (injection volume 20 μL). The gradient was as follows: constant 100% (A) at 0–6 min, a linear gradient from 100 to 30% (A) at 6–21 min, a linear gradient from 30% to 0 (A) at 21–24 min, constant 100% (B) at 24–28 min, a linear gradient from 0 to 100% (A) at 28–30 min, and constant 100% (A) at 30–35 min. The eluent was monitored by diode array detection between 200 and 400 nm. Desulfoglucosinolates were identified by comparing retention times and UV absorption spectra with those of known standards. Results are given as μmol·g^−1^ dry weight, calculated using peak areas and generally agreed relative response factors for UV detection at 229 nm: 0.95 in case of **1**, **2R**, **2S** and the internal standard benzylglucosinolate, 0.50 in case of the phenolic **3R**, 0.29 in case of **4**, 0.25 in case of **5**, and 0.20 in case of **6** (Agerbirk et al., 2015).

### Database for glucosinolate biosynthetic genes identification in *B. vulgaris*

Sequences representing the complete set of glucosinolate biosynthetic genes in *A. thaliana* were acquired from the TAIR database (www.arabidopsis.org). Data for G- and P-type *B. vulgaris* transcriptome sequence were obtained from previous pyrosequencing studies [ftp://shanjie:shanjie123@brassicadb.org and EMBL/NCBI/SRA (accession numbers SRR1582492 and SRR1583630); Wei et al., 2013; Zhang et al., 2015]. We identified candidate genes related to glucosinolate biosynthesis and transcription factors of *B. vulgaris* using BLASTN with a cutoff *E*-value ≤ 1E-10.

### RNA extraction and first-strand cDNA synthesis

Total RNA of the samples was isolated using an *EasyPure*® Plant RNA Kit (TransGen Biotech, China), according to the manufacturer's instructions. 800 ng of total RNA was reverse transcribed to synthesize first-strand cDNA using oligo dT primers and *EasyScript*® One-Step gDNA Removal and cDNA Synthesis SuperMix (TransGen Biotech, China) and diluted 20-fold as templates for molecular cloning and qPCR.

### Quantitative real-time PCR (qPCR)

Quantitative real-time PCR was performed on a StepOne™ Real-Time PCR System (Applied Biosystems), using the *TransStart*®Green qPCR SuperMix (TransGen Biotech), following the manufacturer's instructions. Primers were designed using Primer3web (version 4.0.0, http://primer3.ut.ee/; Untergasser et al., 2012). A list of genes and primers is shown in Table S1. The reaction volume was 20 μL, including 0.4 μL of 10 mM Forward and Reverse primer respectively, 10 μL of 2 × TransStart Green qPCR SuperMix, 2.0 μL of the cDNA sample, 0.4 μL of Passive Reference Dye I, and 6.8 μL of ddH_2_O. The thermal cycling profile was: 95°C for 10 min; 40 cycles of 95°C for 15 s, 59°C for 15 s, 72°C for 10 s; then 95°C for 15 s, 60°C for 1 min, ramping to 95°C for 15 s. Three independent biological and technical replicates were performed. Data were analyzed using StepOne™ Software v.2.0 (Applied Biosystems). *Tubulin* was used as an internal control. The relative expression level were estimated by the 2^−ΔΔCT^ method (Livak and Schmittgen, 2001).

### Gene cloning, sequencing, and sequence analysis

Reverse transcription (RT)-PCR cloning was performed to confirm the assembly quality of genes involved in glucosinolate biosynthesis. Specific PCR primers for the six selected genes were designed corresponding to the ends of longer unigenes of G-type and P-type *B. vulgaris*, using Primer3web (version 4.0.0, http://primer3.ut.ee/; Untergasser et al., 2012). The list of genes and primers is shown in Table S1. PCR was performed in a total volume of 50 μL, including 5 μL of 10 × PCR buffer, 5 μL of 25 mM MgSO_4_, 3 μL of 2 mM dNTPs, 1.5 μL of each 10 mM primer, 1 μL of 1.0 U/μL KOD-Plus-Neo polymerase (Toyobo, Osaka, Japan), 2 μL of cDNA and 31 μL of ddH_2_O, with the following reactions: an initial denaturation step at 94°C for 2 min; followed by 35 cycles of 98°C for 10 s and 68°C for 60 s. The PCR products were separated on 1% (w/v) agarose gel and isolated using a MaxiGel Extraction Kit (CoWin Biotech, Beijing, China), ligated into the *pEASY*®-Blunt Cloning vector (TransGen Biotech, Beijing, China), and then transformed into *Escherichia coli* DH 5α. Positive clones were confirmed by PCR and sequenced using an ABI 3730 instrument (Applied Biosystems, CA, USA). Sequence data alignment and amino acid deduce and alignment were performed using DNAMAN version 8 (Lynnon, Quebec, Canada) with the default parameters.

### Statistical analysis

The SPSS 17.0 software package for Windows was used for all statistical analyses. The data were analyzed for significant differences using Tukey's HSD test at a significance threshold of *p* = 0.05.

## Results

### A pair of phenethyl glucosinolate epimers is dominant in G- and P-type *B. vulgaris* and inducible by DBM

**Figure 2:**
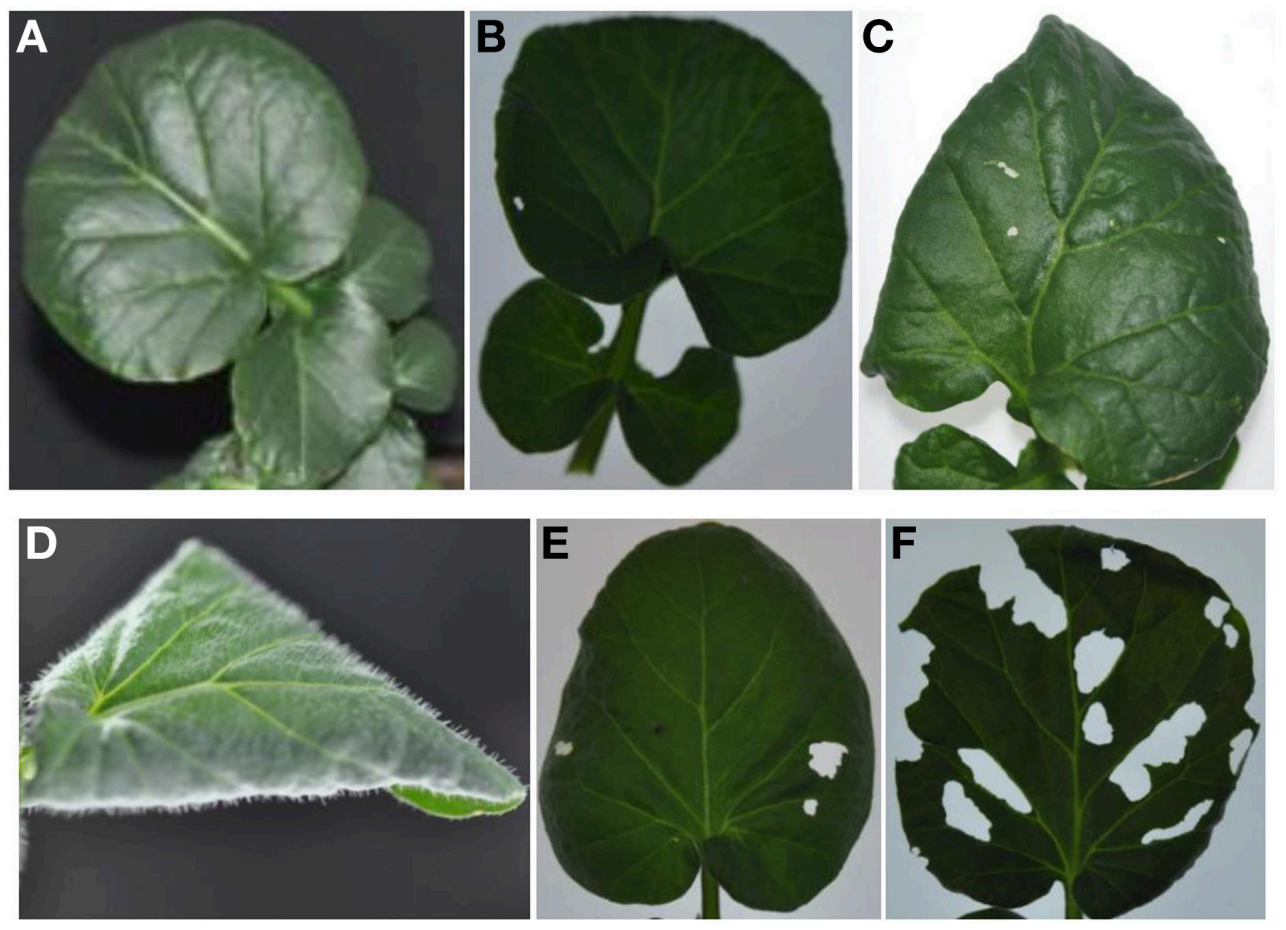
**G-type (A–C) and P-type (D–F) *B. vulgaris* leaves exposed to larvae of the diamondback moth (*Plutella xylostella*) over 8 and 48 h**. **(A,D)**, control leaves; **(B,E)**, leaves treated with three DBM third-instar larvae for 8 h; **(C,F)**, leaves treated with three DBM third-instar larvae for 48 h.

**Figure 3:**
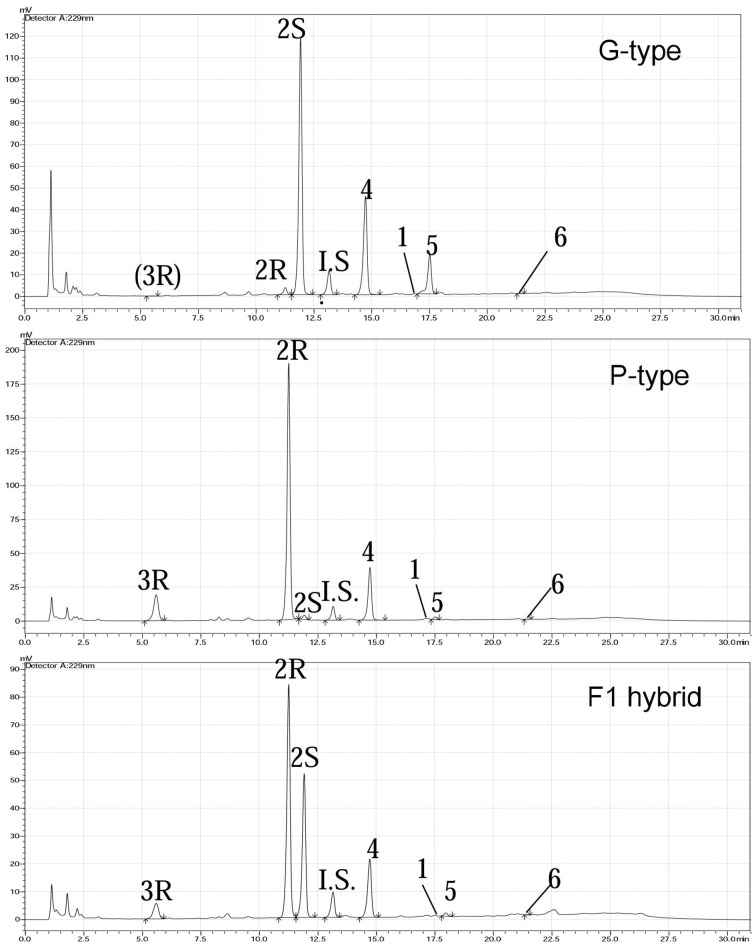
**Quantification of glucosinolates from G-type, P-type, and hybrid F_1_ plants by HPLC after desulfation of the native metabolites and an added standard**. The desulfated glucosinolates, in order of elution, are: **3R**, (2*R*)-2-hydroxy-2-(4-hydroxyphenylethyl) GSL (4-hydroxyepiglucobarbarin); **2R**, (2*R*)-2-hydroxy-2-phenylethyl GSL (epiglucobarbarin); **2S**, (2*S*)-2-hydroxy-2-phenylethyl GSL (glucobarbarin); **I.S**, Glucotropaeolin (internal standard); **4**, 3-indolylmethyl GSL (glucobrassicin); **1**. 2-phenylethyl GSL (gluconasturtiin); **5**, 4-methoxy-3-indolylmethyl GSL (4-methoxyglucobrassicin); **6**, *N*-methoxy-3-indolylmethyl. Trace levels of **3R** in G-type represents maximum estimates based on integration of a trace signal at the same retention time, and is not proof of presence of this glucosinolate in the G-type.

We examined leaf damage, glucosinolate types and their quantities of G- and P-type *B. vulgaris* over 0, 8, and 48 h after DBM infestation. The DBM-resistance ability differed significantly between the G- and P-type *B. vulgaris*, as evaluated by the fraction of leaf area damaged. G-type plants were more resistant and only suffered minor injuries, while about a quarter of P-type leaves were damaged at 48 h after DBM infestation (Figure 2). Glucosinolate profiling indicated that seven types of major glucosinolates were present in the leaves of *B. vulgaris* (Table 1 and Figure 3). The dominant class was aromatic glucosinolates with a phenethyl backbone, of which the most abundant compound was glucobarbarin (**2S**) in G-type and epiglucobarbarin (**2R**) in the P-type (Figures 1, 3); both of them were induced by DBM infestation (Table 1). As previously reported for the P-type, the phenolic glucosinolate **3R**, apparently a ring-oxidized derivative of **2R**, was also detected but at moderate levels. Neither **3R** nor its *S*-epimer has ever been detected in the G-type by specific HPLC-MS. However, for purely statistical reasons a trace signal with similar retention time was quantitated for the G-type (Table 1, Figure 3). Glucobrassicin (**4**) was the most abundant indole glucosinolate in both types, accompanied by the 4-methoxy derivative (**5**) and traces of the *N*-methoxy derivative (**6**). Indole glucosinolates accumulated more abundant in the G-type. Only levels of three individual glucosinolates could be induced by DBM infestation: **2S** (only in G-type), **2R** (only in P-type) and **4** (only in G-type). Because the dominating glucosinolate in each plant type was induced, total glucosinolate levels were also “induced” in both types, but only due to changes in the three mentioned individual glucosinolates. The contents of both **2S** and **4** in the G-type were gradually induced at 8 and 48 h, while the P-type's response to DBM infestation was more rapid: significant induction of epiglucobarbarin was observed at 8 h, with no significant difference with 48 h (Table 1). In relative terms, the induction of the glucobarbarins (**2R** and **2S**) was similar, around 1.4-fold in 48 h. This extend of induction was comparable to the mean induction of the indole glucosinolate glucobrassicin in the G-type (around 1.5-fold). In absolute terms the induction of **2R** in the P-type was moderately higher than the induction of **2S** in the G-type, reflecting a higher glucosinolate level in this type at our growth conditions (Table 1).

**Table 1:** **Glucosinolate contents in rosette leaves of G-type and P-type *Barbarea vulgaris* under diamondback moth larvae infestation**.

***B. vulgaris* type**	**Time after DBM infestation**	**1 (μmol/g Dw)**	**2S (μmol/g Dw)**	**2R (μmol/g Dw)**	**3R (μmol/g Dw)**	**4 (μmol/g Dw)**	**5 (μmol/g Dw)**	**6 (μmol/g Dw)**	**Total (μmol/g Dw)**
G-type	0 h	0.262 ± 0.104 b	29.572 ± 0.122 c	0.893 ± 0.009 d	0.008 ± 0.002 b	1.197 ± 0.018 c	0.344 ± 0.035 a	0.001 ± 0.001 a	32.277 ± 0.286 e
	8 h	0.324 ± 0.012 a	32.121 ± 0.114 b	1.079 ± 0.086 d	0.017 ± 0.004 b	1.576 ± 0.019 b	0.346 ± 0.003 a	0.001 ± 0.001 a	35.464 ± 0.230 d
	48 h	0.123 ± 0.040 b	42.122 ± 0.407 a	1.088 ± 0.017 d	0.013 ± 0.002 b	1.819 ± 0.034 a	0.312 ± 0.033 a	0.001 ± 0.001 a	45.478 ± 0.579 c
P-type	0 h	0.388 ± 0.035 a	0.888 ± 0.004 d	51.585 ± 0.120 c	4.486 ± 0.024 a	1.144 ± 0.015 d	0.034 ± 0.002 b	0.002 ± 0.001 a	58.527 ± 0.112 b
	8 h	0.322 ± 0.031 a	0.933 ± 0.007 d	67.329 ± 0.165 b	4.709 ± 0.018 a	0.976 ± 0.001 e	0.032 ± 0.002 b	0.002 ± 0.001 a	74.303 ± 0.054 a
	48 h	0.310 ± 0.008 a	1.068 ± 0.034 d	69.273 ± 0.496 a	4.148 ± 0.022 a	0.968 ± 0.010 e	0.029 ± 0.002 b	0.002 ± 0.001 a	75.798 ± 0.494 a

*The data were presented as mean ± standard error (N = 5 plants of each type and for each infestation type). Different letters in the same column indicate significant differences at 0.05 level (Tukey's HSD test). Trace levels of ***3R*** in G-type represents maximum estimates based on integration of a trace signal at the same retention time, and is not proof of presence of this glucosinolate in the G-type. Glucosinolate numbers according to Figure 1*.

### Prediction of glucosinolate metabolic pathway in *B. vulgaris* and its candidate genes response to DBM infestation

**Figure 4:**
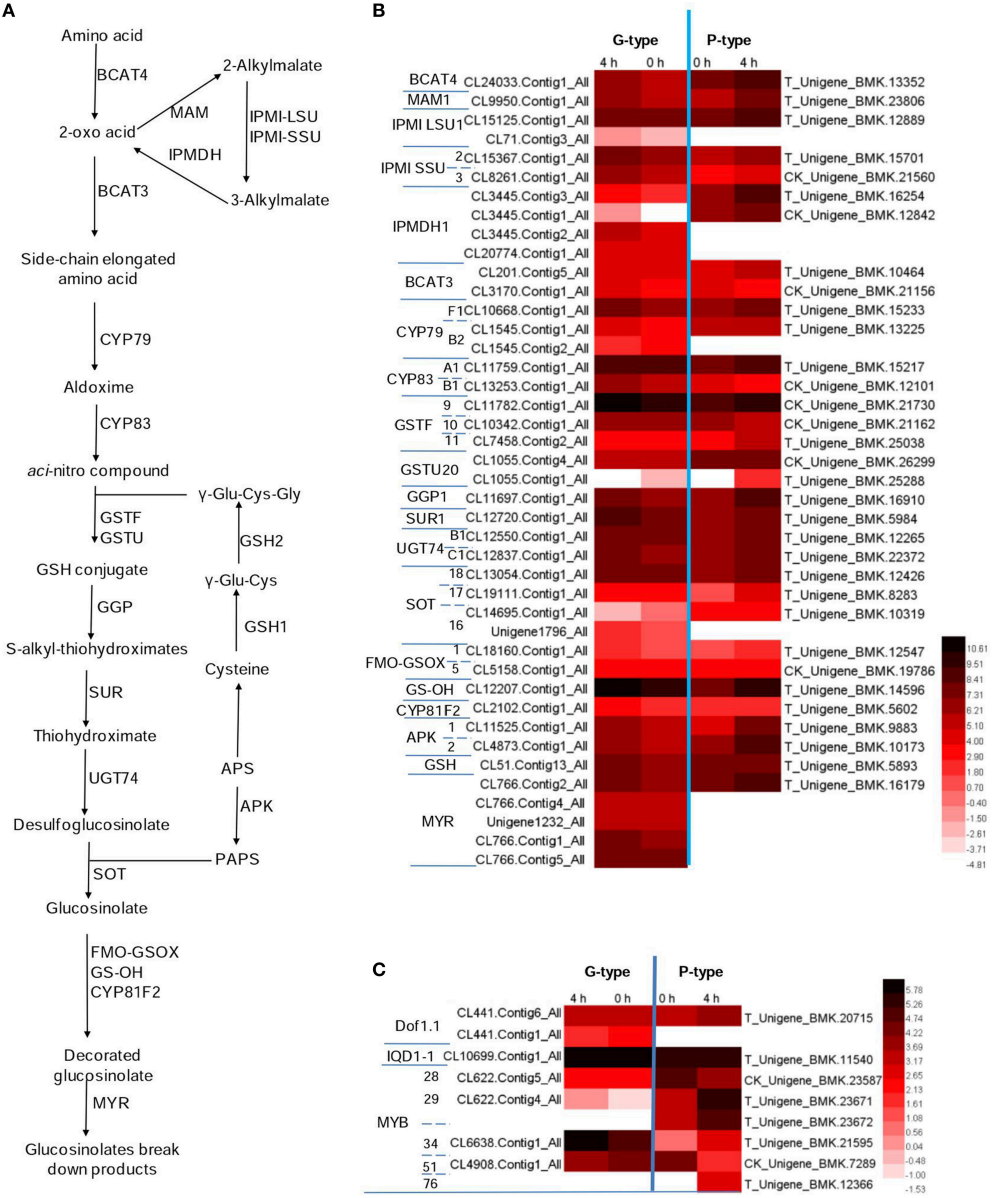
**Potential glucosinolates biosynthesis and degradation pathway in *Barbarea vulgaris***. **(A)** Glucosinolate biosynthesis and degeneration pathway adopted from *Arabidopsis thaliana*. Because the enzymology in *B. vulgaris* is yet unknown, enzyme names (e.g., BCAT) in *A. thaliana* are indicated even if corresponding enzymes involved in aromatic glucosinolate biosynthesis in *B. vulgaris* would have other names and specificities **(B)** Differentially expressed genes in *B. vulgaris* types potentially involved in glucosinolate biosynthesis and degeneration. **(C)** Differentially expressed transcription factors potentially involved in regulating glucosinolate biosynthesis in *B. vulgaris* types. The color code indicates differential expression values and the scale on the right represents gene expression ration values, log_2_ of reads per kilo bases per million reads (RPKM) of plants treated 0 and 4 h of G-type and P-type after DBM feeding. BCAT, branched-chain aminotransferase; BAT, bile acid transporter; MAM, methylthioalk-ylmalate synthase; IPMI, isopropylmalate isomerase; IPMDH, isopropylmalate dehydrogenase; CYP, cytochromes P450; GSTF, glutathione S-transferase; GSTU, glutathione S-transferase tau; GGP, gamma-glutamyl peptidase; SUR, C-S lyase SUPERROOT; UGT, UDP-dependent glycosyl transferases; SOT, sulfotransferase; GS-OH, 2-oxoglutarate (2OG) and Fe(II)-dependent oxygenase superfamily protein; MYR, myrosinase; FMO-GSOX, flavin-monooxygenase glucosinolate S-oxygenase; APK, Adenylyl-sulfate kinase; GSH, gamma-glutamylcysteine synthetase; Dof, Dof zinc finger protein; MYB, MYB domain protein; APS, adenosine-5′-phosphosulfate; PAPS, 3′-phosphoadenosine-5′-phosphosulfate.

To investigate the molecular basis of the glucosinolate biosynthesis in *B. vulgaris*, a glucosinolates metabolic pathway was deduced according to the KEGG pathway (PATHWAY: map00966) and previous reports (Sønderby et al., 2010; Figure 4A). The pathway is generally characterized into four stages: (i) chain elongation of selected precursor amino acids (e.g., Met and Phe), (ii) core structure formation, (iii) secondary modification of the side chain, and (iv) degradation of glucosinolate. Forty-two G-type and 33 P-type unigenes were identified as candidates of the 30 enzymes in the pathway, by homology searching from our former reported *B. vulgaris* transcriptome dataset with the glucosinolate metabolic genes from *A. thaliana* as baits. The gene list and their corresponding *Arabidopsis* homolog AGI codes, as well as the sequence similarities with G- or P-type, are shown in Tables S2 and S4.

The biosynthesis pathway of methionine derived glucosinolates starts from the side-chain elongation process catalyzing the parent amino acid deamination to form 2-oxo acid by a branched-chain amino acid aminotransferase (BCAT4; Figure 4A and Table S3). In our annotated *B. vulgaris* transcriptome unigene dataset, a unigene corresponding to *BCAT4* was identified in both G- and P-type plants. It was significantly induced by DBM in the P-type, but not significantly in the G-type plant (Figure 4B and Table S3). The 2-oxo acid then enters a cycle of three successive transformations (Sønderby et al., 2010): (1) condensation with acetyl-CoA by MAM (methylthioalkylmalate synthase); (2) isomerization by IPMI LSU (isopropylmalate isomerase large subunit) and IPMI SSU (isopropylmalate isomerase small subunit); and (3) oxidative decarboxylation by IPMDH (isopropylmalate dehydrogenase). A similar chain elongation is also needed for biosynthesis of phenethyl glucosinolates (Figure 1, **1**–**3**), but would not be relevant for the tryptophan derived (Figure 1, **4**–**6**). One *MAM*, two *IPMI LSU*, two *IPMI SSU*, four *IPMDH* transcripts were identified in G-type while one *MAM*, one *IPMI LSU*, two *IPMI SSU*, and two *IPMDH* transcripts were identified in P-type plants. All of these genes were upregulated by DBM infestation in P-type plants but not in the G-type (Figure 4B and Table S3). Thereafter, the molecules are transaminated by BCAT3. Two unigenes encoding BCAT3 were identified in both G- and P-type plants, amongst, the genes in the P-type were induced by DBM. The products of the above reactions then enter the core glucosinolate structure pathway.

The formation of the glucosinolate core structure is accomplished by 13 enzymes catalyzing five biochemical steps (glutathione S-transferase (GSTF), glutathione S-transferase tau (GSTU) are predicted and gamma-glutamyl peptidase (GGP) is a partially characterized enzyme; they are considered as one step along with CYP83 in previous and current reports; Grubb and Abel, 2006; Sønderby et al., 2010). It begins with the oxidation of the side-chain elongated amino acids to convert them to aldoximes by cytochromes P450 of the CYP79 family. There are seven *CYP79* genes in the *Arabidopsis* genome and one of them (*CYP79A2*) uses Phe as substrate. A high similarity homolog of *CYP79A2* was not found in *B. vulgaris*. However, three and two *CYP79s* were identified in G- and P-type *B. vulgaris* respectively. Among them, CL10668.Contig1_−_All in G-type and T_Unigene_BMK.15233 in P-type had higher expression levels and were induced by DBM. They are most likely the candidate genes for the enzyme involved in phenethyl glucosinolate biosynthesis. Next step is catalyzed by cytochrome P450 of the CYP83 family. Two unigenes in each G- and P-type *B. vulgaris* were identified as homologs to the two *Arabidopsis CYP83* genes (*CYP83A1* and *CYP83B1*). The *CYP83B1* were classified and named *CYP83B1v1* (G-type) and *CYP83B1v2* (P-type) based on sequence comparisons. This was also done for additional *B. vulgaris CYPs* mentioned below. The next step involves conjugation with a sulfur donor to form a GSH conjugate. Two predicted enzymes, encoded by *GSTF* and *GSTU*, may be involved in this reaction, but this reaction can also happen non-enzymatically (Sønderby et al., 2010). Three homolog unigenes of *GSTF*, two homolog unigenes of *GSTU* were identified in G- and P-type *B. vulgaris*. The above steps were not significantly affected by DBM infestation. In *Arabidopsis*, there is good evidence that the sulfur donor is glutathione, γ-Glu-Cys-Gly, and its biosynthesis involves five committed enzymes, including ATPs, APR, OASTL, GSH1, and GSH2 (Geu-Flores et al., 2009, 2011). One unigene was discovered encoding GSH in G- and P-type, respectively. The GSH conjugate is then hydrolyzed by GGP (Geu-Flores et al., 2009) to form an *S*-alkyl-thiohydroximate, which is subsequently degraded by C-S lyase SUPERROOT1 (SUR1) to form a thiohydroximate, which is in turn S-glucosylated by glucosyltransferase UGT74 to form a desulfoglucosinolate. One *GGP*, one *SUR1* and two *UGT74* homolog sequences were discovered in G- and P-type *B. vulgaris*, respectively. All of them were significantly induced by DBM infestation in both types of plants, except *UGT74B1*, in G-type plants. The final step is catalyzed by desulfoglucosinolate sulfotransferase (SOT) to generate the glucosinolate itself, with 3-phosphoadenosine-5′-phosphosulfate (PAPS) as the sulfate donor. Four and three unigenes were identified as *SOTs* in G- and P-type *B. vulgaris*. One of them, T_Unigene_BMK.12426 in the P-type had the highest expression level and was upregulated by DBM (Figure 4B and Table S3). PAPS is produced from adenosine-5′-phosphosulfate (APS) through a two-step catalysis by ATP sulfurylase (ATPS) and APS kinase (APK; Sønderby et al., 2010). In the present study, two DBM inducible unigenes were discovered encoding APKs in G- and P-type *B. vulgaris*, respectively. By the end of these steps, the parent phenethyl glucosinolate, 2-phenylethylglucosinolate (**1**) would be produced.

Both methionine derived glucosinolates and phenethyl glucosinolates are further subjected to hydroxylation modification, resulting in increased structural diversity. However, existing genetic and enzymological knowledge concerns methionine-derived glucosinolates. The flavin-monooxygenase glucosinolate S-oxygenase (FMO-GSOX), alkenyl hydroxalkyl producing (AOP), Fe (II)-dependent oxygenase superfamily protein (GS-OH) and CYP81F2 are reported to take part in these processes and other side chain modification (Hansen et al., 2007; Wentzell et al., 2007; Li et al., 2008; Bednarek et al., 2009; Clay et al., 2009; Pfalz et al., 2009). Assuming that related genes would be responsible for hydroxylation of phenethyl glucosinolates, we searched for homologs in our transcriptome. Two FMO-GSOX and one CYP81F2 homolog sequences were discovered in G- and P-type *B. vulgaris*, respectively. These *CYPs* were classified and named *CYP81F1v1* (G-type) and *CYP81F1v2* (P-type). However, unigenes responsible for AOP were not detected. In this study, CL12207.Contig1_−_All in G- and T_−_Unigene_−_BMK.14596 in P-type *B. vulgaris* were homologous to the *Arabidopsis GS-OH*, which is responsible for modification of 3-butenyl glucosinolate to produce 2-hydroxy-3-butenyl glucosinolate. These two genes were expressed at high levels and were upregulated by 2.5- and 4.4-fold in G- and P-types, respectively, under DBM infestation (Figure 4B, Tables S2, S3).

Five unigenes in G-type plants were identified as homologs of genes encoding myrosinase, while only one unigene was found in P-type. All of these genes were slightly upregulated by DBM but did not fulfill the two-fold cutoff in both types (Figure 4B and Table S3).

### Candidate regulatory transcription factor genes and their response to DBM infestation

It was reported that three transcription factors (IQD1-1, Dof1.1 and MYB) could regulate the expression of genes involved in glucosinolate metabolism in *A. thaliana* (Wang et al., 2011). Two and one homolog sequences were discovered encoding Dof1.1 in G- and P-type *B. vulgaris*, respectively. One unigene was predicted to encode IQD1-1 in both plant types. These two types of transcription factors were not significantly affected by DBM in both plant types (Figure 4C and Table S3). Six members of the MYB family (MYB28, 29, 34, 51, 76, and 122) are reported to regulate the biosynthesis of glucosinolates (Gigolashvili et al., 2008; Sonderby et al., 2010). From our *B. vulgaris* transcriptome, one member of each of *MYB28, 29, 34*, and *51* homolog was found in the G-type and one homolog of each of *MYB28, 34, 51*, and *76* and two homologs of *MYB29* were identified in P-type plants. *MYB28* was downregulated in the P-type but not in the G-type *B. vulgaris*. *MYB29* was significantly upregulated in both types, but accumulated more than 27-fold higher in the P-type compared with the G-type plants after 4 h of infestation by DBM. While *MYB34* accumulated more than nine-fold more in the G-type than in the P-type. *MYB51* was downregulated in both plant types. *MYB76* was only identified in P-type pants and was induced by DBM (Figure 4C and Table S3). Thus, the induction of the glucosinolate pathway is possibly regulated by *MYB34* in the G-type and by *MYB29* and *MYB76* in the P-type.

### qPCR confirmation of glucosinolate genes expression patterns

**Figure 5:**
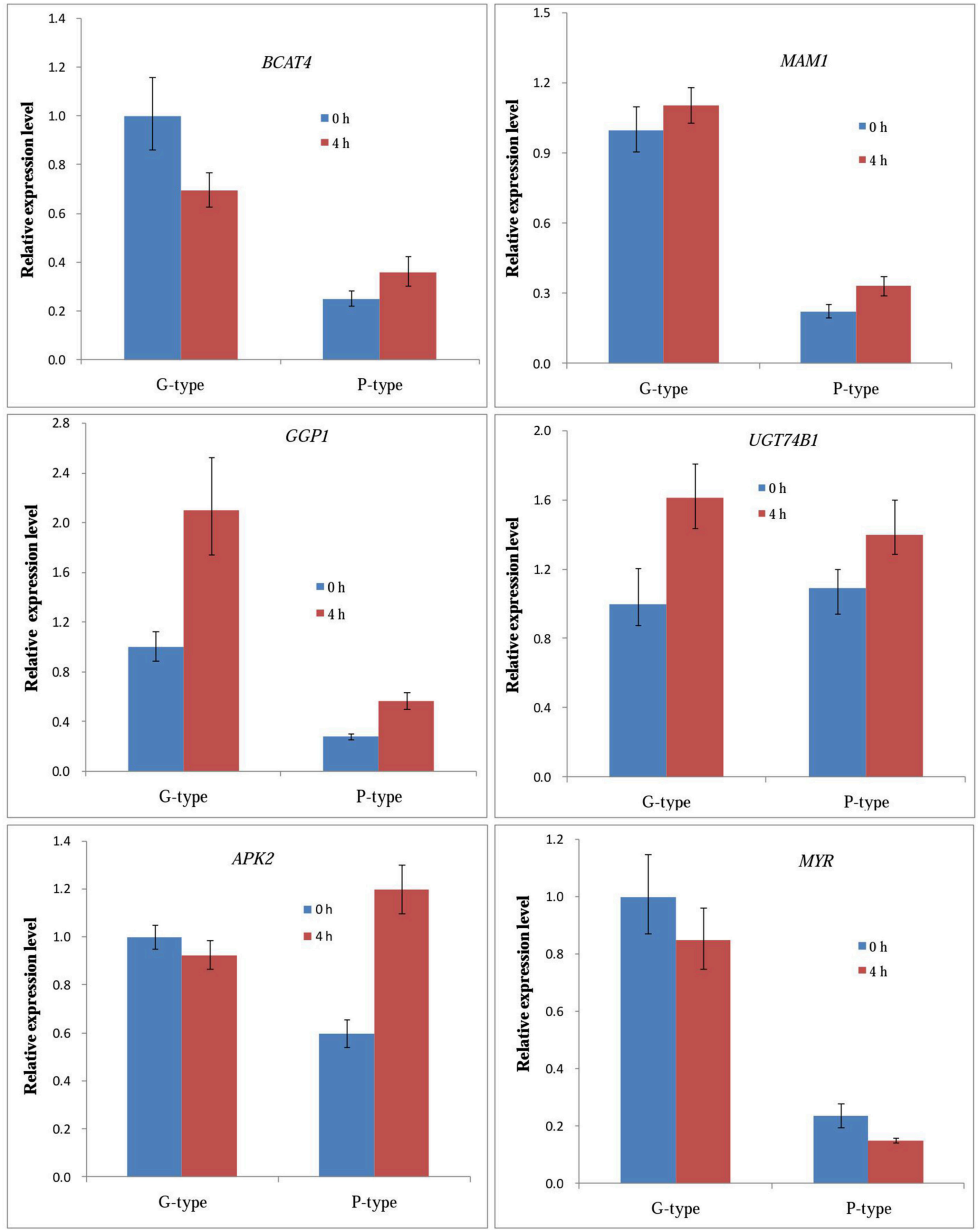
**qRT-PCR expression analysis of expression levels of six selected genes in leaves of *Barbarea vulgaris* at 0 h (control) and 4 h after being infested by diamondback moth larvae**.

We confirmed the expression patterns of six genes responsible for glucosinolates biosynthesis in leaves of *B. vulgaris* before and after (4 h) DBM infestation using qPCR. The expression patterns of these genes are shown in Figure 5. Most genes showed good correlation with the profiles from transcriptome sequencing. The unigenes encoding MAM1, GGP1, and UGT74B1 showed the same expression patterns between the two genotypes, both were upregulated at 4 h after DBM infestation. While the transcription of myrosinases was suppressed in both plant types. The *BCAT4* genes were significantly downregulated in G-type plants, but upregulated in P-type. In contrast to *BCAT4*, the *APK2* gene was stable in the G-type, while they were significantly upregulated in the P-type by DBM infestation.

### Molecular cloning and comparison of genes involved in glucosinolates metabolism

**Figure 6:**
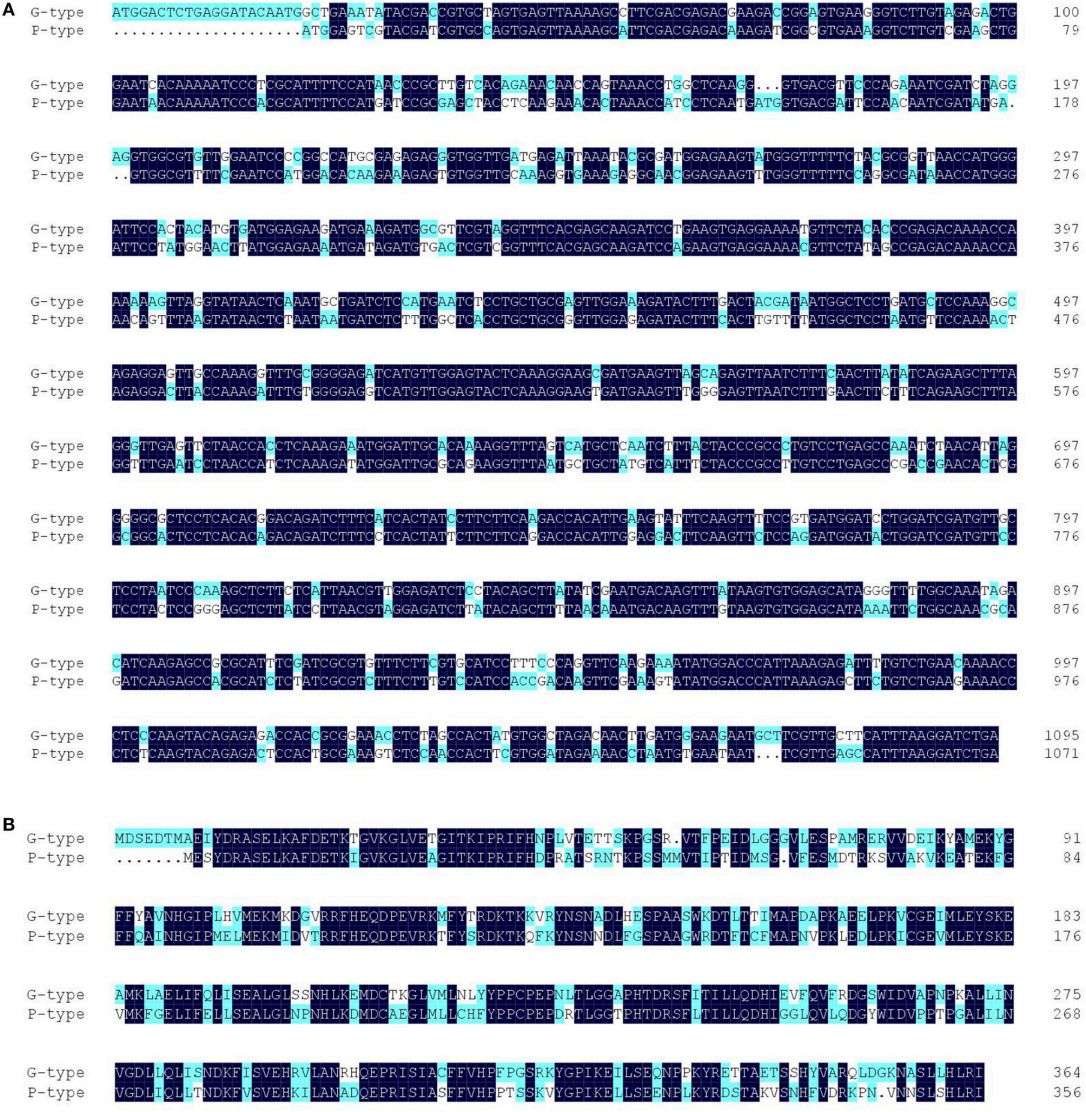
**Sequence alignment of the full-length coding DNA sequences (A) and deduced amino acid sequences (B) of *GS-OH* homologes of G-type (*SHO*) and P-type (*RHO*) *Barbarea vulgaris***.

Several RT-PCR cloning experiments were performed to check the quality of the unigene assembly. cDNA sequences of six selected genes [*BCAT4, CYP83A1, GGP1, SUR1, GS-OH* (*SHO* in G-type and *RHO* in P-type)] from the glucosinolate biosynthetic pathway were isolated from both G- and P-type *B. vulgaris* by blunt end cloning and were subsequently sequenced using the Sanger method. The sequence similarities between the clones and corresponding unigenes were more than 99.2% pairwise identity, which validated the reliability of RNA-seq assembly (Table 2). Additionally, sequences similarities between G- and P-type were also compared, showing that the genes in both types were highly conserved (>99.0% pairwise identity) except for *GS-OH*, which showed significantly sequence variation (77.50% pairwise identity in coding DNA sequences and 65.48% identity in deduced amino acid sequences) between the two types (Table 2, Figure 6). The sequence diversity of *GS-OH* could be responsible for the different glucobarbarin epimers of the G-type and P-type glucosinolates (glucobarbarin, **2S**, vs. epiglucobarbarin, **2R**). Furthermore, the known biochemical function of GS-OH is equivalent to the functions envisioned for SHO and RHO: β-hydroxylation (Figure 1) of a glucosinolate side chain (Hansen et al., 2008). Thus, we named the *GS-OH* in G- and P-type separately as *SHO* and *RHO*, respectively, in accordance with a previous hypothesis put forward by Agerbirk et al. (2015).

**Table 2:** **Sequence analyses of the six genes putatively involved in glucosinolate biosynthesis in two types of *B. vulgaris***.

**Gene**	***B. vulgaris* type**	**Sequence length**	**Similarity with transcriptome (%)**	**Similarity between G- and P-type (%)**
BCAT4	G-type	1064	99.4	99.0
	P-type	1064	99.2	
CYP83A1	G-type	1506	99.7	98.9
	P-type	1506	99.7	
GGP1	G-type	750	99.6	99.7
	P-type	750	100.0	
SUR1	G-type	1344	100.0	99.0
	P-type	1344	100.0	
SHO	G-type	1095	100.0	78.0 (compared with RHO)
	P-type	–	–	
RHO	G-type	–	–	–
	P-type	1071	99.5	

### A genetic model in which SHO and RHO produce the S and R epimers of glucobarbarins in *B. vulgaris*

**Figure 7:**
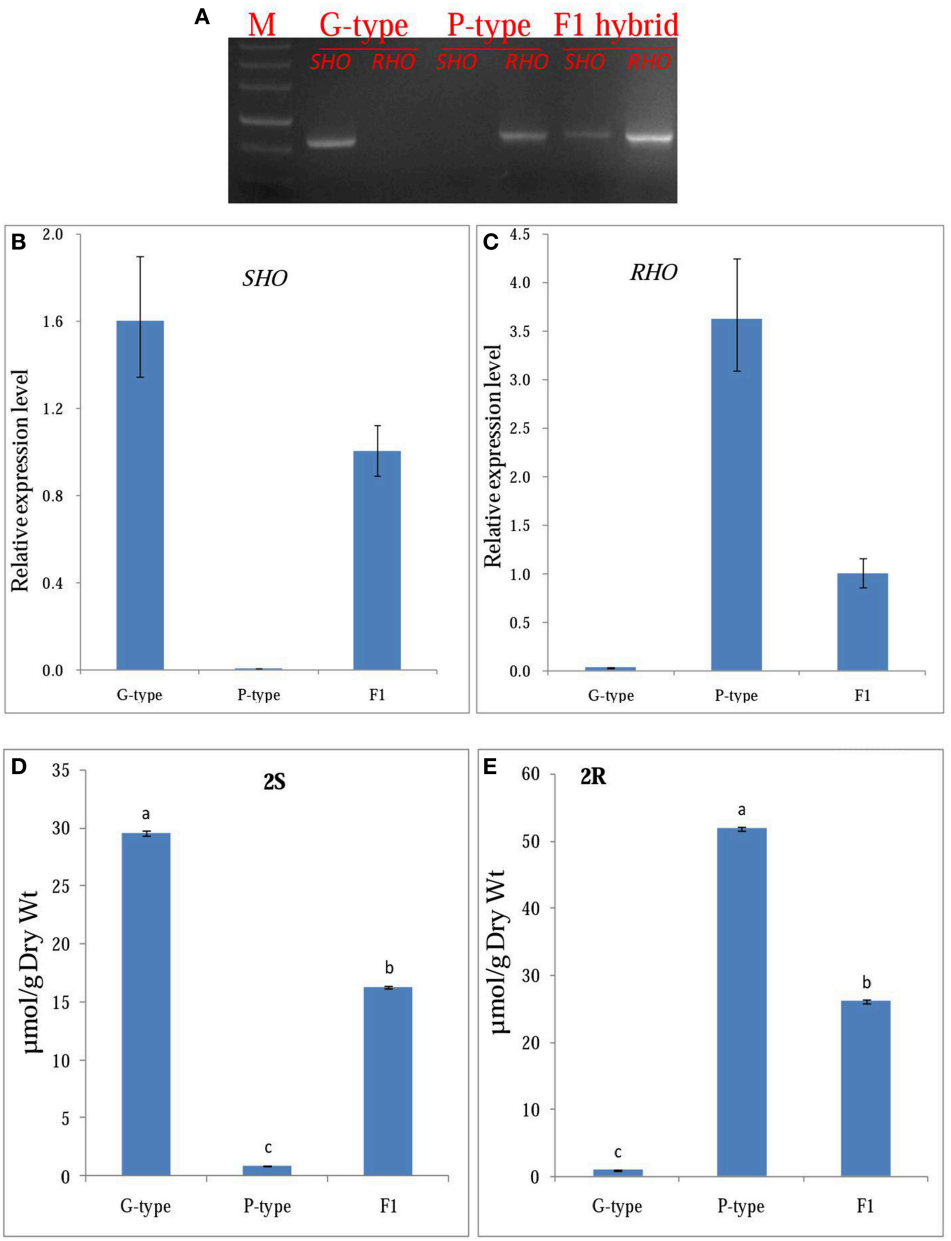
**Functional confirmation of candidate *GS-OH* gene**. **(A)** gene expression of the *SHO, RHO* in *Barbarea vulgaris* leaves of G-type, P-type, and F_1_ (generated by hybridization of G- and P-type plants). **(B,C)**, qRT-PCR expression analysis of *SHO, RHO* in *Barbarea vulgaris* leaves of G-type, P-type and F_1_, respectively. **(D,E)**, Mean (± SE) concentration in rosette leaves of *Barbarea vulgaris* of G- and P-type. **2S**, (2*S*)-2-hydroxy-2-phenylethyl GSL (glucobarbarin); **2R**, (2*R*)-2-hydroxy-2-phenylethyl GSL (epiglucobarbarin).

To test the hypothesis that CL12207.Contig1_−_All and T_−_Unigene_−_BMK.14596 are the *GS-OH* genes responsible for the glucosinolate epimers in the G-type (*SHO*) and P-type (*RHO*) *B. vulgaris*, we generated F_1_ plants by hybridization of G- and P-type plants and analyzed the correlation between gene expression and glucosinolate production. The *SHO* and **2S**, *RHO* and **2R** co-occurred in G-, P-type, and F_1_ plants (Figure 7). For testing co-segregation, cooccurrence of gene and suggested glucosinolate product in F_2_ plants was needed. Unfortunately, F_2_ plants were not available in this project. Indeed, an F_2_ generation from a G-type × P-type cross appears to have been obtained only once in the literature (Kuzina et al., 2011), while further F_2_ progenies have not been published, possibly due to a highly frequent sterility barrier between the accessions of the types that have so far been tested. Based on the available results, we established a double-codominance gene model to explain the **2S** and **2R** inheritance in the two types of *B. vulgaris*. In this model, the double heterozygote SsRr plants (F_1_) generated by hybridization of SSrr (G-type, mainly containing **2S**) and ssRR (P-type, mainly containing **2R**) will contain both epimers (**2S** and **2R**; Figures 3, 7). Therefore, F_2_ populations should contain recessive homozygous plants (ssrr), which only accumulated precursor glucosinolate, **1** (or traces of the glucobarbarin epimers if additional minor genes were present). A previous study showed that the profile of five plants in 129 F_2_ *B. vulgaris* population were dominated by **1** (Kuzina et al., 2011), which supports our codominance-gene model. The model is further supported by observation of low levels of epiglucobarbarin in so-called “NAS-forms” of the G-type devoid of glucobarbarin (van Leur et al., 2006; Agerbirk et al., 2015).

## Discussion

Glucosinolates can be divided into groups according to their amino acid precursor, of which we consider three: methionine (Met), phenylalanine (Phe), and tryptophan (Trp) including chain-elongated homologs of the former two. Glucosinolates derived from Met (including chain elongated homologs) and Trp are well-studied and their biosynthetic pathways have been identified in the model plant *A. thaliana* (Sønderby et al., 2010). These served as a model for our search for genes involved in the biosynthetic pathway of aromatic Phe-derived glucosinolates. This is currently unknown, although a recent review lists a couple of genes that may be involved in their biosynthesis in natural or engineered systems (*CYP79A2, CYP83A1, CYP83B1, SUR1, UGT74B1*, and *SOT16*; Baskar et al., 2012). In an evolutionary perspective, Phe-derived glucosinolates should be divided in non-chain elongated glucosinolates directly derived from Phe and named “benzyl glucosinolates,” and chain elongated derived from homoPhe and named “phenethyl glucosinolates.” The former, without chain elongation, are believed to be an ancient character, while the latter seem more recent and occur as the core structure gluconasturtiin (**1**) in roots of most cruciferous crops, and as oxidized derivatives in *Barbarea* and occasionally elsewhere. *B. vulgaris* is dominated by oxidized phenethyl glucosinolates (Agerbirk et al., 2015), making it an ideal plant for the study of the phenethyl glucosinolate biosynthetic pathway including side chain decoration. In this study, a potential glucosinolate biosynthesis pathway was manually predicted by homology with biosynthesis of Met-derived glucosinolates in *Arabidopsis* and a number of candidate genes in this pathway were discovered in G- and P-type *B. vulgaris*. As Met-derived glucosinolates are not known from *Barbarea*, the genes would seem to be involved in biosynthesis of phenethyl glucosinolates. Our model will serve as a starting point for exploring the biosynthesis of phenethyl glucosinolates at the molecular level. Furthermore, we found the GS-OH candidate genes *SHO* and *RHO* in G- and P-type plants, which are most likely responsible for the stereospecific biosynthesis of glucobarbarin and its epimer, epiglucobarbarin. The functions of the genes identified in this study require confirmation by further studies, such as expression in *A. thaliana*. The transcription factors involved in regulating the glucosinolate biosynthetic pathway, including Dof1.1, IQD1-1, and MYB, were also identified successfully in G- and P-type *B. vulgaris*. Based on their expression patterns, the MYB34 in the G-type and MYB29 and MYB76 in the P-type were deduced as regulators of the glucosinolate induction in the DBM response.

Recently, it has been speculated that resistant and susceptible plant types could have diverged during the ice age because of geographical isolation and adaption to their new environment, leading to the formation of different evolutionary lineages and taxa, which differ in their resistance, hairiness, saponins, flavonoids, and glucosinolates (Hauser et al., 2012; Christensen et al., 2014). The results of the present study showed that upstream genes of GS-OH were highly conserved during evolution: the sequence similarity between the two genotypes exceeded 98.9%. Unexpectedly, the *SHO* and *RHO* have significantly sequence variation between G- and P-type *B. vulgaris*, as low as 77.5% in coding DNA sequences and 65.48% identity in deduced amino acid sequences (Figure 6). Thus, we consider them as two independent genes that may have diverged during the separation of the two types.

There is ample evidence that glucosinolates not only function in defense against generalist herbivores, but also play a key role in host recognition for crucifer specialist insects (Mewis et al., 2005; Badenes-Pérez et al., 2011). Previous reports on *A. thaliana* indicated that both generalist and specialist insects can induce glucosinolate synthesis pathways, while the transcription of myrosinases was suppressed (Kuśnierczyk et al., 2007). In our previous study using the same data set, the P-type glucosinolate biosynthesis pathway was not over-represented among the upregulated pathways by a hypergeometric test, mainly because many differentially expressed genes have not been annotated to the glucosinolate biosynthesis pathway by the automatic KEGG annotation pipeline. In our present study, the glucosinolate biosynthesis pathways were constructed manually and refined, and the expression level of the most glucosinolate synthesis genes were increased in G- and P-type *B. vulgaris* after DBM infestation, as revealed by transcriptome and qPCR experiments, which is consistent with previous reports in *Arabidopsis* and *Brassica* plants (Kuśnierczyk et al., 2007).

Herbivory is probably a multifaceted challenge of plants given that the wounds from the herbivore provide a direct access for pathogenic microbes as well as increased evaporation, stress from released phytochemicals, etc. Hence, it is likely that plant responses to insect herbivory should include not only defenses against the herbivore, but also defenses against a variety of microbes and other stresses. Furthermore, induction responses may not be specific for each herbivore, but produce a response that on average has defensive properties against a range of frequent herbivores. For these reasons, it is not surprising that some glucosinolates were induced by DBM larvae despite the resistance of the larvae to this defense. Indeed, a similar induction of **2S** and **2R** by a flea beetle was recently reported (van Mölken et al., 2014). Quantitatively, the reported induction was similar to the induction reported here. However, we find it striking that the massive tissue damage in the P-type (Figure 1) did not result in induction of the indole glucosinolate **4**, which is in many plant species a highly inducible glucosinolate (Bodnaryk, 1992; Hopkins et al., 1998; Bartlet et al., 1999). In contrast, the modest tissue damage in the G-type (Figure 2) never-the-less induced **4**. Furthermore, it is interesting that the phenolic **3R**, believed to be biosynthesized from **2R**, was not induced in the P-type, although the apparent precursor was induced. In contrast, the phenolic **3R** was reported to be many fold induced in the transition from summer to fall (Agerbirk and Olsen, 2015) suggesting that environmental regulation of glucosinolate hydroxylation in *B. vulgaris* is complex. A gene sequence (*PHO*) for this hydroxylation of **2R** to **3R** is not suggested here, but the availability of the P-type transcriptome (Zhang et al., 2015), and the present refinement, now provide candidates for future investigations of glucosinolate regulation in the species. From this investigation, the relevant gene would be expected to be unique for the P-type and not be induced by DBM herbivory. This hydroxylation is known to have functional significance, as the hydrolysis product of **3R** is a thiazolidine-2-one, in contrast to the oxazolidine-2-thione produced from **2R** and **2S**.

The DBM is one of the most destructive pests of crucifer crops causing about $ 4-5 billion loss annually in the world (Furlong et al., 2013). It is reported to have developed resistance to all major classes of insecticides including *Bacillus thuringiensis* (*Bt*) insecticidal proteins (Shelton, 2004; Furlong et al., 2013). In China, high dose of insecticide with short interval time are commonly used to control DBM, which causes severe environmental damage and food contamination. Therefore, an integrated pest management is urgently needed. Previous studies on agricultural uses of *B. vulgaris* mostly focus on the use as a “dead-end” trap crop (Lu et al., 2004; Badenes-Pérez et al., 2005) and mining resistance genes for breeding insect resistant cultivars (Wei et al., 2013; Khakimov et al., 2015; Zhang et al., 2015). In recent reports, we have confirmed the resistance-properties of G-type *B. vulgaris* to a contemporary Chinese field-isolate of DBM (Wei et al., 2013; Liu et al., 2015b; Zhang et al., 2015), further implying the application potential of this wild crucifer in the DBM controlling. Our present research shows that DBM infection induced the content of glucosinolate, an oviposition attracting signal for DBM, in agreement with usage of *B. vulgaris* as a “dead-end” trap for DBM control. On the other hand, DBM chewing can be envisioned to expose the plant to pathogenic microbes which may lead to additional loss for Brassicaceae vegetable production. The DBM induction of glucosinolates possibly could reduce pathogenic microbes' access via the wounds rather than waste of resources, thus improving insect resistance or tolerance, respectively, of G-type and P-type *B. vulgaris*.

In conclusion, the present study identified genes involved in glucosinolate biosynthesis of G- and P-type *B. vulgaris*, and characterized the relationship between gene expression patterns and glucosinolate contents in response DBM. These findings will deepen our understanding of the biosynthesis of the phenethyl group of aromatic glucosinolates at the molecular level and provide the basis for further investigation of the molecular ecology of insect resistance in *B. vulgaris* plants.

## Author contributions

Conceived and designed the experiments: XL, TL, XZ. Performed the experiments: TL, XZ, HY, JS. Analyzed the data: TL, XZ, XL, NA, YQ, HW, DS. Wrote the paper: TL, XZ, XL, NA. All authors read and approved the final manuscript.

### Conflict of interest statement

The authors declare that the research was conducted in the absence of any commercial or financial relationships that could be construed as a potential conflict of interest.

## References

[B1] Agerbirk N. Olsen C. E. (2012). Glucosinolate structures in evolution. Phytochemistry 77, 16–45. 10.1016/j.phytochem.2012.02.005 22405332

[B2] Agerbirk N. Olsen C. E. (2015). Glucosinolate hydrolysis products in the crucifer *Barbarea vulgaris* include a thiazolidine-2-one from a specific phenolic isomer as well as oxazolidine-2-thiones. Phytochemistry 115, 143–151. 10.1016/j.phytochem.2014.11.002 25467719

[B3] Agerbirk N. Olsen C. E. Cipollini D. Ørgaard M. Linde-Laursen I. Chew F. S. (2014). Specific glucosinolate analysis reveals variable levels of epimeric glucobarbarins, dietary precursors of 5-phenyloxazolidine-2-thiones, in watercress types with contrasting chromosome number. J. Agric. Food Chem. 62, 9586–9596. 10.1021/jf5032795 25226408

[B4] Agerbirk N. Olsen C. E. Heimes C. Christensen S. Bak S. Hauser T. P. (2015). Multiple hydroxyphenethyl glucosinolate isomers and their tandem mass spectrometric distinction in a geographically structured polymorphism in the crucifer *Barbarea vulgaris*. Phytochemistry 115, 130–142. 10.1016/j.phytochem.2014.09.003 25277803

[B5] Badenes-Pérez F. R. Reichelt M. Gershenzon J. Heckel D. G. (2011). Phylloplane location of glucosinolates in *Barbarea* spp. *(Brassicaceae)* and misleading assessment of host suitability by a specialist herbivore. New Phytol. 189, 549–556. 10.1111/j.1469-8137.2010.03486.x 21029103

[B6] Badenes-Pérez F. R. Reichelt M. Heckel D. G. (2010). Can sulfur fertilisation improve the effectiveness of trap crops for diamondback moth, *Plutella xylostella* (L.) (Lepidoptera: Plutellidae)? Pest Manage. Sci. 66, 832–838. 10.1002/ps.1949 20603876

[B7] Badenes-Pérez F. R. Shelton A. M. Nault B. A. (2005). Using yellow rocket as a trap crop for diamondback moth (Lepidoptera: Plutellidae). J. Econ. Entomol. 98, 884–890. 10.1603/0022-0493-98.3.884 16022317

[B8] Bartlet E. Kiddle G. Williams I. Wallsgrove R. (1999). Wound-induced increases in the glucosinolate content of oilseed rape and their effect on subsequent herbivory by a crucifer specialist, in Proceedings of the 10th International Symposium on Insect-Plant Relationships (Oxford: Springer), 163–167.

[B9] Baskar V. Gururani M. A. Yu J. W. Park S. W. (2012). Engineering glucosinolates in plants: current knowledge and potential uses. Appl. Biochem. Biotechnol. 168, 1694–1717. 10.1007/s12010-012-9890-6 22983743

[B10] Bednarek P. Piślewska-Bednarek M. Svatoš A. Schneider B. Doubský J. Mansurova M.. (2009). A glucosinolate metabolism pathway in living plant cells mediates broad-spectrum antifungal defense. Science 323, 101–106. 10.1126/science.1163732 19095900

[B11] Bodnaryk R. P. (1992). Effects of wounding on glucosinolates in the cotyledons of oilseed rape and mustard. Phytochemistry 31, 2671–2677. 10.1016/0031-9422(92)83609-3

[B12] Christensen S. Heimes C. Agerbirk N. Kuzina V. Olsen C. E. Hauser T. P. (2014). Different geographical distributions of two chemotypes of *Barbarea vulgaris* that differ in resistance to insects and a pathogen. J. Chem. Ecol. 40, 491–501. 10.1007/s10886-014-0430-4 24777484

[B13] Clay N. K. Adio A. M. Denoux C. Jander G. Ausubel F. M. (2009). Glucosinolate metabolites required for an *Arabidopsis* innate immune response. Science 323, 95–101. 10.1126/science.1164627 19095898 PMC2630859

[B14] Dalby-Brown L. Olsen C. E. Nielsen J. K. Agerbirk N. (2011). Polymorphism for novel tetraglycosylated flavonols in an eco-model crucifer, *Barbarea vulgaris*. J. Agric. Food Chem. 59, 6947–6956. 10.1021/jf200412c 21615154

[B15] Furlong M. J. Wright D. J. Dosdall L. M. (2013). Diamondback moth ecology and management: problems, progress, and prospects. Annu. Rev. Entomol. 58, 517–541. 10.1146/annurev-ento-120811-153605 23020617

[B16] Geu-Flores F. Moldrup M. E. Böttcher C. Olsen C. E. Scheel D. Halkier B. A. (2011). Cytosolic γ-glutamyl peptidases process glutathione conjugates in the biosynthesis of glucosinolates and camelexin in *Arabidopsis*. Plant Cell 23, 2456–2469. 10.1105/tpc.111.083998 21712415 PMC3160024

[B17] Geu-Flores F. Nielsen M. T. Nafisi M. Møldrup M. E. Olsen C. E. Motawia M. S.. (2009). Glucosinolate engineering identifies a γ-glutamyl peptidase. Nat. Chem. Boil. 5, 575–577. 10.1038/nchembio.185 19483696

[B18] Gigolashvili T. Engqvist M. Yatusevich R. Mueller C. Fluegge U. I. (2008). Hag2/myb76 and hag3/myb29 exert a specific and coordinated control on the regulation of aliphatic glucosinolate biosynthesis in *Arabidopsis thaliana*. New Phytol. 177, 627–642. 10.1111/j.1469-8137.2007.02295.x 18042203

[B19] Grubb C. D. Abel S. (2006). Glucosinolate metabolism and its control. Trends Plant Sci. 11, 89–100. 10.1016/j.tplants.2005.12.006 16406306

[B20] Hansen B. G. Kerwin R. E. Ober J. A. Lambrix V. M. Mitchell-Olds T. Gershenzon J.. (2008). A novel 2-oxoacid-dependent dioxygenase involved in the formation of the goiterogenic 2-hydroxybut-3-enyl glucosinolate and generalist insect resistance in Arabidopsis. Plant Physiol. 148, 2096–2108. 10.1104/pp.108.129981 18945935 PMC2593654

[B21] Hansen B. G. Kliebenstein D. J. Halkier B. A. (2007). Identification of a flavin-monooxygenase as the *S*-oxygenating enzyme in aliphatic glucosinolate biosynthesis in Arabidopsis. Plant J. 50, 902–910. 10.1111/j.1365-313X.2007.03101.x 17461789

[B22] Hauser T. P. Toneatto F. Nielsen J. K. (2012). Genetic and geographic structure of an insect resistant and a susceptible type of *Barbarea vulgaris* in western europe. Evol. Ecol. 26, 611–624. 10.1007/s10682-011-9515-5

[B23] Hopkins R. Griffiths D. Birch A. McKinlay R. (1998). Influence of increasing herbivore pressure on modification of glucosinolate content of swedes (*Brassica napus* spp. rapifera). J. Chem. Ecol. 24, 2003–2019. 10.1023/A:1020729524818

[B24] Khakimov B. Poulsen V. K. Erthmann P. Ø. Fukushima E. O. Augustin J. M. Olsen C. E.. (2015). Identification and genome organization of saponin pathway genes from a wild crucifer, and their use for transient production of saponins in *Nicotiana benthamiana*. Plant J. 84, 478–490. 10.1111/tpj.13012 26333142

[B25] Kuchernig J. C. Burow M. Wittstock U. (2012). Evolution of specifier proteins in glucosinolate-containing plants. BMC Evol. Biol. 12:127. 10.1186/1471-2148-12-127 22839361 PMC3482593

[B26] Kuśnierczyk A. Winge P. Midelfart H. Armbruster W. S. Rossiter J. T. Bones A. M. (2007). Transcriptional responses of *Arabidopsis thaliana* ecotypes with different glucosinolate profiles after attack by polyphagous myzus persicae and oligophagous *Brevicoryne brassicae*. J. Exp. Bot. 58, 2537–2552. 10.1093/jxb/erm043 17545220

[B27] Kuzina V. Ekstrom C. T. Andersen S. B. Nielsen J. K. Olsen C. E. Bak S. (2009). Identification of defense compounds in *Barbarea vulgaris* against the herbivore *Phyllotreta nemorum* by an ecometabolomic approach. Plant Physiol. 151, 1977–1990. 10.1104/pp.109.136952 19819983 PMC2785962

[B28] Kuzina V. Nielsen J. K. Augustin J. M. Torp A. M. Bak S. Andersen S. B. (2011). *Barbarea vulgaris* linkage map and quantitative trait loci for saponins, glucosinolates, hairiness and resistance to the herbivore *Phyllotreta nemorum*. Phytochemistry 72, 188–198. 10.1016/j.phytochem.2010.11.007 21130479

[B29] La G. X. Fang P. Teng Y. B. Li Y. J. Lin X. Y. (2009). Effect of CO_2_ enrichment on the glucosinolate contents under different nitrogen levels in bolting stem of Chinese kale (*Brassica alboglabra* L.). J. Zhejiang Univ. Sci. B. 10, 454–464. 10.1631/jzus.B0820354 19489111 PMC2689558

[B30] Li J. Hansen B. G. Ober J. A. Kliebenstein D. J. Halkier B. A. (2008). Subclade of flavin- monooxygenases involved in aliphatic glucosinolate biosynthesis. Plant Physiol. 148, 1721–1733. 10.1104/pp.108.125757 18799661 PMC2577257

[B31] Liu T. J. Zhang X. H. Li X. X. Shen D. Wang H. P. Qiu Y. (2015a). Advances on research and utilization of elite resistant resource - *Barbarea vulgris*. Acta Hortic. Sin. 42, 1719–1731. 10.16420/j.issn.0513-353x.2015-0178

[B32] Liu T. J. Zhang X. H. Shen D. Wang H. P. Qiu Y. Song J. P. (2015b). Analysis on genetic diversity of *Barbarea vulgris* germplasm resources based on phenotypic traits. J. Plant Genet. Resour. 16, 528–534. 10.13430/j.cnki.jpgr.2015.03.014

[B33] Livak K. J. Schmittgen T. D. (2001). Analysis of relative gene expression data using real-time quantitative PCR and the 2^−ΔΔCT^ method. Methods 25, 402–408. 10.1006/meth.2001.1262 11846609

[B34] Lu J. H. Liu S. S. Shelton A. M. (2004). Laboratory evaluations of a wild crucifer *Barbarea vulgaris* as a management tool for the diamondback moth *Plutella xylostella* (Lepidoptera: Plutellidae). Bull. Entomol. Res. 94, 509–516. 10.1079/BER2004328 15541190

[B35] Mewis I. Appel H. M. Hom A. Raina R. Schultz J. C. (2005). Major signaling pathways modulate arabidopsis glucosinolate accumulation and response to both phloem-feeding and chewing insects. Plant Physiol. 138, 1149–1162. 10.1104/pp.104.053389 15923339 PMC1150428

[B36] Nielsen J. K. (1997). Variation in defences of the plant *Barbarea vulgaris* and in counter adaptations by the flea beetle *Phyllotreta nemorum*. Entomol. Exp. Appl. 82, 25–35. 10.1046/j.1570-7458.1997.00110.x

[B37] Pedras M. S. C. Alavi M. To Q. H. (2015). Expanding the nasturlexin family: nasturlexins C and D and their sulfoxides are phytoalexins of the crucifers *Barbarea vulgaris* and *B. verna*. Phytochemistry 118, 131–138. 10.1016/j.phytochem.2015.08.009 26318326

[B38] Pfalz M. Vogel H. Kroymann J. (2009). The gene controlling the indole glucosinolate modifier1 quantitative trait locus alters indole glucosinolate structures and aphid resistance in Arabidopsis. Plant Cell 21, 985–999. 10.1105/tpc.108.063115 19293369 PMC2671713

[B39] Rasmann S. Chassin E. Bilat J. Glauser G. Reymond P. (2015). Trade-off between constitutive and inducible resistance against herbivores is only partially explained by gene expression and glucosinolate production. J. Exp. Bot. 66, 2527–2534. 10.1093/jxb/erv033 25716695 PMC4986863

[B40] Ratzka A. Vogel H. Kliebenstein D. J. Mitchell-Olds T. Kroymann J. (2002). Disarming the mustard oil bomb. Proc. Natl. Acad. Sci. U.S.A. 99, 11223–11228. 10.1073/pnas.172112899 12161563 PMC123237

[B41] Shelton A. M. (2004). Management of the diamondback moth: déjà vu all over again? in the management of diamondback moth and other crucifer pests, in Diamondback Moth and Other Crucifer Pests: Proceedings of the Fourth International Workshop Management, eds Endersby N. M. Ridland P. M. (Melbourne, VIC: Regional Institute), 3–8.

[B42] Shinoda T. Nagao T. Nakayama M. Serizawa H. Koshioka M. Okabe H.. (2002). Identification of a triterpenoid saponin from a crucifer, *Barbarea vulgaris*, as a feeding deterrent to the diamondback moth, *Plutella xylostella*. J. Chem. Ecol. 28, 587–599. 10.1023/A:1014500330510 11944835

[B43] Sonderby I. E. Burow M. Rowe H. C. Kliebenstein D. J. Halkier B. A. (2010). A complex interplay of three R2R3 MYB transcription factors determines the profile of aliphatic glucosinolates in Arabidopsis. Plant Physiol. 153, 348–363. 10.1104/pp.109.149286 20348214 PMC2862430

[B44] Sønderby I. E. Geu-Flores F. Halkier B. A. (2010). Biosynthesis of glucosinolates–gene discovery and beyond. Trends Plant Sci. 15, 283–290. 10.1016/j.tplants.2010.02.005 20303821

[B45] Toneatto F. Nielsen J. K. Orgaard M. Hauser T. P. (2010). Genetic and sexual separation between insect resistant and susceptible *Barbarea vulgaris* plants in Denmark. Mol. Ecol. 19, 3456–3465. 10.1111/j.1365-294X.2010.04760.x 20670365

[B46] Untergasser A. Cutcutache I. Koressaar T. Ye J. Faircloth B. C. Remm M.. (2012). Primer3-new capabilities and interfaces. Nucleic Acids Res. 40, e115–e115. 10.1093/nar/gks596 22730293 PMC3424584

[B47] van Leur H. Raaijmakers C. E. van Dam N. M. (2006). A heritable glucosinolate polymorphism within natural populations of *Barbarea vulgaris*. Phytochemistry 67, 1214–1223. 10.1016/j.phytochem.2006.04.021 16777152

[B48] van Leur H. Vet L. E. Van der Putten W. H. van Dam N. M. (2008). *Barbarea vulgaris* glucosinolate phenotypes differentially affect performance and preference of two different species of lepidopteran herbivores. J. Chem. Ecol. 34, 121–131. 10.1007/s10886-007-9424-9 18213497 PMC2239252

[B49] van Mölken T. Kuzina V. Munk K. R. Olsen C. E. Sundelin T. van Dam N. M.. (2014). Consequences of combined herbivore feeding and pathogen infection for fitness of *Barbarea vulgaris* plants. Oecologia 175, 589–600. 10.1007/s00442-014-2928-4 24687328

[B50] Wang H. Wu J. Sun S. Liu B. Cheng F. Sun R.. (2011). Glucosinolate biosynthetic genes in *Brassica rapa*. Gene 487, 135–142. 10.1016/j.gene.2011.07.021 21835231

[B51] Wang Y. Pan Y. Liu Z. Zhu X. Zhai L. Xu L.. (2013). *De novo* transcriptome sequencing of radish (*Raphanus sativus* L.) and analysis of major genes involved in glucosinolate metabolism. BMC Genomics 14:836. 10.1186/1471-2164-14-836 24279309 PMC4046679

[B52] Wei X. C. Zhang X. H. Shen D. Wang H. P. Wu Q. J. Lu P.. (2013). Transcriptome analysis of *Barbarea vulgaris* infested with diamondback moth (*Plutella xylostella*) larvae. PLoS ONE 8:e64481. 10.1371/journal.pone.0064481 23696897 PMC3655962

[B53] Wentzell A. M. Rowe H. C. Hansen B. G. Ticconi C. Halkier B. A. Kliebenstein D. J. (2007). Linking metabolic QTLs with network and cis-eQTLs controlling biosynthetic pathways. PLoS Genet. 3:e162. 10.1371/journal.pgen.0030162 17941713 PMC1976331

[B54] Wittstock U. Halkier B. A. (2002). Glucosinolate research in the *Arabidopsis* era. Trends Plant Sci. 7, 263–270. 10.1016/S1360-1385(02)02273-2 12049923

[B55] Zhang X. H. Liu T. J. Wei X. C. Qiu Y. Song J. P. Wang H. P.. (2015). Expression patterns, molecular markers and genetic diversity of insect-susceptible and resistant *Barbarea* genotypes by comparative transcriptome analysis. BMC Genomics 16:486. 10.1186/s12864-015-1609-y 26126637 PMC4487577

