# Supplementary material for: Aromatic Glucosinolate Biosynthesis Pathway in Barbarea vulgaris and its Response to Plutella xylostella Infestation
Source: Front Plant Sci. 2016 Feb 8;7:83. doi: 10.3389/fpls.2016.00083 (PMC4744896; doi:10.3389/fpls.2016.00083)
Supplement: Supplementary file 2 [file Table2.DOCX]

Table S2 Gene list of the glucosinolate pathway and *B. vulgaris* orthologs

| Glucosinolate pathway | Gene name | Corresponding AGI | *B. vulgaris* type | *B. vulgaris* ID | sequence similarity with *Arabidopsis thaliana* |
| --- | --- | --- | --- | --- | --- |
| Amino acid side chain  elongation | BCAT4 | At3g19710 | G-type | CL24033.Contig1_All | 908/1055 (86%) |
|  |  |  | P-type | T_Unigene_BMK.13352 | 908/1055 (86%) |
|  | MAM1 | At5g23010 | G-type | CL9950.Contig1_All | 1002/1154 (87%) |
|  |  |  | P-type | T_Unigene_BMK.23806 | 1231/1422 (87%) |
|  | MAM3 | At5g23020 |  | - |  |
|  | IPMI LSU1 | At4g13430 | G-type | CL15125.Contig1_All | 668/711 (93%) |
|  |  |  |  | CL71.Contig3_All | 759/832 (91%) |
|  |  |  | P-type | T_Unigene_BMK.12889 | 1422/1539 (92%) |
|  | IPMI SSU2 | At2g43100 | G-type | CL15367.Contig1_All | 674/777 (87%) |
|  |  |  | P-type | T_Unigene_BMK.15701 | 676/777 (87%) |
|  | IPMI SSU3 | At3g58990 | G-type | CL8261.Contig1_All | 661/755 (88%) |
|  |  |  | P-type | CK_Unigene_BMK.21560 | 667/758 (88%) |
|  | IPMDH1 | At5g14200 | G-type | CL3445.Contig3_All | 1087/1230 (88%) |
|  |  |  |  | CL3445.Contig2_All | 1006/1125 (89%) |
|  |  |  |  | CL20774.Contig1_All | 986/1125 (88%) |
|  |  |  |  | CL3445.Contig1_All | 903/989 (91%) |
|  |  |  | P-type | T_Unigene_BMK.16254 | 1126/1230 (92%) |
|  |  |  |  | CK_Unigene_BMK.12842 | 976/1125 (87%) |
|  | IPMDH3 | At1g31180 | - | - | - |
|  | BCAT-3 | At3g49680 | G-type | CL201.Contig5_All | 1117/1263 (88%) |
|  |  |  |  | CL3170.Contig1_All | 989/1230 (80%) |
|  |  |  | P-type | T_Unigene_BMK.10464 | 1116/1263 (88%) |
|  |  |  |  | CK_Unigene_BMK.21156 | 1001/1252 (80%) |
| Core structure formation | CYP79F1 | At1g16410 | G-type | CL10668.Contig1_All | 1407/1615 (87%) |
|  |  |  | P-type | T_Unigene_BMK.15233 | 1406/1615 (87%) |
|  | CYP79F2 | At1g16400 | - | - | - |
|  | CYP79A2 | At5g05260 | - | - | - |
|  | CYP79B2 | At4g39950 | G | CL1545.Contig1_All | 1509/1626 (93%) |
|  |  |  |  | CL1545.Contig2_All | 1421/1626 (87%) |
|  |  |  | P | T_Unigene_BMK.13225 | 1512/1626 (93%) |
|  | CYP79B3 | At2g22330 | - | - | - |
|  | CYP83A1 | At4g13770 | G | CL11759.Contig1_All | 1336/1508 (89%) |
|  |  |  | P | T_Unigene_BMK.15217 | 1343/1508 (89%) |
|  | CYP83B1 | At4g31500 | G | CL13253.Contig1_All | 1270/1394 (91%) |
|  |  |  | P | CK_Unigene_BMK.12101 | 1364/1500 (91%) |
|  | GSTF9 | At2g30860 | G | CL11782.Contig1_All | 582/636 (92%) |
|  |  |  | P | CK_Unigene_BMK.21730 | 584/636 (92%) |
|  | GSTF10 | At2g30870 | G | CL10342.Contig1_All | 583/648 (90%) |
|  |  |  |  | CK_Unigene_BMK.21162 | 472/588 (80%) |
|  | GSTF11 | At3g03190 | G | CL7458.Contig2_All | 593/645 (92%) |
|  |  |  | P | T_Unigene_BMK.25038 | 595/645 (92%) |
|  | GSTU20 | At1g78370 | G | CL1055.Contig4_All | 479/615 (78%) |
|  |  |  |  | CL1055.Contig1_All | 470/619 (76%) |
|  |  |  | P | CK_Unigene_BMK.26299 | 485/622 (78%) |
|  |  |  |  | T_Unigene_BMK.25288 | 464/639 (73%) |
|  | GGP1 | At4g30530 | G | CL11697.Contig1_All | 672/753 (89%) |
|  |  |  | P | T_Unigene_BMK.16910 | 673/753 (89%) |
|  | SUR1 | At2g20610 | G | CL12720.Contig1_All | 1218/1345 (91%) |
|  |  |  | P | T_Unigene_BMK.5984 | 1211/1345 (90%) |
|  | UGT74B1 | At1g24100 | G | CL12550.Contig1_All | 1252/1383 (91%) |
|  |  |  | P | T_Unigene_BMK.12265 | 1246/1383 (90%) |
|  | UGT74C1 | At2g31790 | G | CL12837.Contig1_All | 1239/1374 (90%) |
|  |  |  | P | T_Unigene_BMK.22372 | 1237/1374 (90%) |
|  | SOT18 | At1g74090 | G | CL13054.Contig1_All | 942/1013 (93%) |
|  |  |  | P | T_Unigene_BMK.12426 | 940/1013 (93%) |
|  | SOT17 | At1g18590 | G | CL19111.Contig1_All | 625/701 (89%) |
|  |  |  | P | T_Unigene_BMK.8283 | 666/744 (90%) |
|  | SOT16 | At1g74100 | G | CL14695.Contig1_All | 453/497 (91%) |
|  |  |  |  | Unigene1796_All | 453/497 (91%) |
|  |  |  | P | T_Unigene_BMK.10319 | 745/808 (92%) |
| Secondary modification | FMO-GSOX1 | At1g65860 | G | CL18160.Contig1_All | 368/411 (90%) |
|  |  |  | P | T_Unigene_BMK.12547 | 669/761 (88%) |
|  | FMO-GSOX2 | At1g62540 | - | - | - |
|  | FMO-GSOX3 | At1g62560 | - | - | - |
|  | FMO-GSOX4 | At1g62570 | - | - | - |
|  | FMO-GSOX5 | At1g12140 | G | CL5158.Contig1_All | 597/692 (86%) |
|  |  |  | P | CK_Unigene_BMK.19786 | 1204/1376 (88%) |
|  | AOP2 | At4g03060 | - | - | - |
|  | AOP3 | At4g03050 | - | - | - |
|  | GS-OH | At2g25450 | G | CL12207.Contig1_All | 892/1086 (82%) |
|  |  |  | P | T_Unigene_BMK.14596 | 888/1068 (83%) |
|  | CYP81F2 | At5g57220 | G | CL2102.Contig1_All | 1005/1412 (71%) |
|  |  |  | P | T_Unigene_BMK.5602 | 1009/1412 (71%) |
| Co-substrate pathways | APK1 | At2g14750 | G | CL11525.Contig1_All | 737/834 (88%) |
|  |  |  | P | T_Unigene_BMK.9883 | 735/834 (88%) |
|  | APK2 | At4g39940 | G | CL4873.Contig1_All | 1035/1188 (87%) |
|  |  |  | P | T_Unigene_BMK.10173 | 1054/1221 (86%) |
|  | GSH1/PAD2 | At4g23100 | G | CL51.Contig13_All | 1461/1572 (93%) |
|  |  |  | P | T_Unigene_BMK.5893 | 1463/1572 (93%) |
| Hydrolysis pathway | MYR | AT5g25980 | G | CL766.Contig2_All | 1415/1774 (80%) |
|  |  |  |  | CL766.Contig4_All | 1402/1768 (79%) |
|  |  |  |  | Unigene1232_All | 1285/1612 (80%) |
|  |  |  |  | CL766.Contig1_All | 1285/1618 (79%) |
|  |  |  |  | CL766.Contig5_All | 797/1029 (77%) |
|  |  |  | P | T_Unigene_BMK.16179 | 1447/1866 (78%) |
| Transcription factors | Dof1.1 | At1g07640 | G | CL441.Contig6_All | 853/972 (87%) |
|  |  |  |  | CL441.Contig1_All | 534/582 (91%) |
|  |  |  | P | T_Unigene_BMK.20715 | 885/1018 (86%) |
|  | IQD1-1 | At3g09710 | G | CL10699.Contig1_All | 837/914 (91%) |
|  |  |  | P | T_Unigene_BMK.11540 | 876/964 (90%) |
|  | MYB28 | At5g61420 | G | CL622.Contig5_All | 969/1113 (87%) |
|  |  |  | P | CK_Unigene_BMK.23587 | 974/1109 (87%) |
|  | MYB29 | At5g07690 | G | \| CL622.Contig4_All \| \| --- \| | 370/391 (94%), 206/235 (87%), 297/362 (82%), |
|  |  |  | P | T_Unigene_BMK.23671 | 207/235 (88%),  299/362 (82%), |
|  |  |  |  | T_Unigene_BMK.23672 | 370/391 (94%), |
|  | MYB34 | At5g60890 | G | CL6638.Contig1_All | 787/906 (86%), |
|  |  |  | P | T_Unigene_BMK.21595 | 793/912 (86%), |
|  | MYB51 | At1g18570 | G | CL4908.Contig1_All | 542/602 (90%), 366/432 (84%) |
|  |  |  | P | CK_Unigene_BMK.7289 | 550/613 (89%), 250/279 (89%), 66/74 (89%) |
|  | MYB76 | At5g07700 | G | - | - |
|  |  |  | P | T_Unigene_BMK.12366 | 841/986 (85%) |
|  | MYB122 | At1g74080 | - | - | - |
