# Supplementary material for: Aromatic Glucosinolate Biosynthesis Pathway in Barbarea vulgaris and its Response to Plutella xylostella Infestation
Source: Front Plant Sci. 2016 Feb 8;7:83. doi: 10.3389/fpls.2016.00083 (PMC4744896; doi:10.3389/fpls.2016.00083)
Supplement: Supplementary file 4 [file Table4.DOCX]

**BCAT4**

>CL24033.Contig1_All 79 1128 minus strand branched-chain aminotransferase 4 [Brassica rapa subsp. pekinensis]

GTCCAAAGCTTTTGTAGACAACTCAAGCACAACAAACTCAAGTTTCTTACACCGCAACTC

GACTCAAACAGCAAATCCATGGCTCCTTCTGCGCAAACTCTTCCTTCAAGTGTTTCGGAC

GAAAAATACGCGAATATGAATTGGGAAGAATTGGGGTTTGGGTTTGTTCGTACGGATAAT

ATGTATGTTACCAAGTGCAAACATGGAGAGAATTTCCAAGAAGGGAAGATTGTTCCCTAT

GCTGAGCTTCAAGTCGACCCTTCCGCTGCAATTCTTAATTATGGACAGGGTCTATATGAA

GGGCTGAAGGCTTACAGGACAGAAGATGGTCGGATTATGATATTCCGACCAGACCAAAAC

GCTCTCCGCCTTCAAATGGGAGCCGAGAGACTCTGTATGCATTATCCTTCGGTCGATCAA

TTCATATCTGCCGTCAAACAAGTTGTTCTTGCCAACAAGAGATGGATTCCTCCTCCGGGG

AAAGGAACATTGTATATTAGGCCAATCTTGTTTGGGAGTGGTCCTCTGCTTGGTTCACTT

CCGGTTCCTGAGTACACCTTCACAGTGTTTGCATGTCCTGTTGGACGTTTTCACAAGGAT

AGCTCAGGGTTGAATCTCAAAATCGAAGACAGGGTTCGCCGAGCTTTTCCTAGTGGAACT

GGTGGTGTGAAGAGTGTTACAAACTATTCTCCTGTTTGGCTAACATTGTCAGAGGCGAAA

AAACAAGGTTTCTCTGATATTTTGTTTTTGGATGCTGCAACTGGCAAAAACGTTGAAGAA

CTTTTCGCATCTAACATTTTCGTCGTGAAGGGAAATGTCGTGTCGACTCCATCAATTTCA

GGAACTATTTTGCCCGGAGTCACAAGAAAAAGTGTCATCGAATTAACTCGTGATTTCGGC

TACAAGGTGGAGGAACGTGTTCTTCCCGTCGAGGACCTTCTCGACGCCGAAGAAGTTTTC

TGTACTGGGACTGCTGCAATTGTGACAAGTATTGCGTCCGTGACTTTCAAAGACAAAAAG

ACCGGATTCAAAACAGGAGAAGAAACATTAGCTACGAAGCTATTCACGACGTTAATGGGT

ATTCAGTCGGGTCAGATCGAGGATACCAAGGGATGGACGGTGGAGATTGACCGGTGCCAC

TAGTGTTGAAACTGTAACTTGACAAATCCCTTATATATGTATGCAAGTTTATATAAGGAA

CATCTGAAGATGTCTCTCTATCTTTGTGTTTGTCATCATGTTGTCATGTCGTGTCGTCGT

TGCAATGTATTTTAAAAAGTTGGTATGTAAGTAAGTTATTTATAACTTTGGCTATAAATG

ATAATCCATGCTCTACAACAAAAAA

> T_Unigene_BMK.13352 gi|237682390|gb|ACR10244.1| 0 gi|237682390|gb|ACR10244.1| branched-chain aminotransferase 4 [Brassica rapa subsp. pekinensis]

AGAGAAGCAGTCCAAAGCTTTTGTAGACAACTCAAGCACAACAAACTCAAGTTTCTTACACCGCAACTCGACTCAAACAGCAAATCCATGGCTCCTTCTGCGCAAACTCTTCCTTCAAGTGTTTCGGACGAAAAATACGCGAATATGAATTGGGAAGAATTGGGGTTTGGGTTTGTTCGTACGGATAATATGTATGTTACCAAGTGCAAACATGGAGAGAATTTCCAAGAAGGGAAGATTGTTCCCTATGCTGAGCTTCAAGTCGACCCTTCCGCTGCAATTCTTAATTATGGACAGGGTCTATATGAAGGGCTGAAGGCTTACAGGACAGAAGATGGTCGGATTATGATATTCCGACCAGACCAAAACGCTCTCCGCCTTCAAATGGGAGCCGAGAGACTCTGTATGCATTATCCTTCGGTCGATCAATTCATATCTGCCGTCAAACAAGTTGTTCTTGCCAACAAGAGATGGATTCCTCCTCCGGGGAAAGGAACATTGTATATTAGGCCAATCTTGTTTGGGAGTGGTCCTCTGCTTGGTTCACTTCCGGTTCCTGAGTACACCTTCACAGTGTTTGCATGTCCTGTTGGACGTTTTCACAAGGATAGCTCAGGGTTGAATCTCAAAATCGAAGACAGGGTTCGCCGAGCTTTTCCTAGTGGAACTGGTGGTGTGAAGAGTGTTACAAACTATTCTCCTGTTTGGCTAACATTGTCAGAGGCGAAAAAACAAGGTTTCTCTGATATTTTGTTTTTGGATGCTGCAACTGGCAAAAACGTTGAAGAACTTTTCGCATCTAACATTTTCGTCGTGAAGGGAAATGTCGTGTCGACTCCATCAATTTCAGGAACTATTTTGCCCGGAGTCACAAGAAAAAGTGTCATCGAATTAACTCGTGATTTCGGCTACAAGGTGGAGGAACGTGTTCTTCCCGTCGAGGACCTTCTCGACGCCGAAGAAGTTTTCTGTACTGGGACTGCTGCAATTGTGACAAGTATTGCGTCCGTGACTTTCAAAGACAAAAAGACCGGATTCAAAACAGGAGAAGAAACATTAGCTACGAAGCTATTCACGACGTTAATGGGTATTCAGTCGGGTCAGATCGAGGATACCAAGGGATGGACGGTGGAGATTGACCGGTGCCAC

**MAM1**

>CL9950.Contig1_All 3 1241 minus strand methylthioalkylmalate synthase [Arabidopsis thaliana] >gi|98985838|emb|CAJ55504.1| methylthioalkylmalate synthase 1 [Arabidopsis thaliana]

GCCTGAATTCCCATCTGAGATTTTCGATCTGGTTATAAAAAATAGTTAAA

TATATAGAAAACACACACCTCAATTATTATATACTGTTTTTATTCGTTAC

AACAGCAAAACATGGATATCAAATGGAACTTGATATCATCGTATTACACA

AACACACAAAAGTGTTCACAAATTTATACAACAGACGGAATCTGGGGGAT

TGATACAGAGCCGTTAGTTCCATTACCATTAAGTTTCCCTGATGAAAGTT

CATCACAGCACACCACTAGTGCCTTCAGATCAGCATCTGTGATTCTCTTT

TTCTCTTTGGTTAAATTCCTGAATCGTGAGAAGATGTTGTTCAATTTCTC

TTCATCGATTTCATATCCCAACTCTTTCAGCCGATCTTTCACAGCATGAC

GCCCACTAAGCTTTCCAAGAACAATTCCAGACTTTAGAGATTTTGAACCC

CCAATATCTTCTGGTGATAAGATCTCATATGTACTCCGATTTTTCAGTAT

TCCATCCTGGTGAATGCCACTCTCATGAACAAAACAGTTGGCTCCAACAA

TGGGCTTATACGCTTGAACATACAAGCCGCCTAGCTCTTGAACCATCTGG

CTAGTAGCCATAATTTGGCGTGTGTCTATATTTGTGTAAACACCATCCAT

AGCATATGCTCCTCGACATTTCAAAGCCATCACAACCTCTTCAAGTGGCG

CATTCCCACTTCTTTCACCTATTCCATTGACCGTTACGTCGACTTGTCTT

GCTCCCGCACATATACCGGCGAGTGTGTTAGCGGTAGCAAGGCCAAGGTC

GTTGTGACAATGAATACTGAAGACAACATCATCAATTCCAGGAGTGTTTG

CTTTGAGATAGGATATGAGTTCTCCGAATTCGTGCGGCACGTTGTACCCT

ACCGTGTCCCCGATGTTCACCGCCGTTGCACCCACTTTTATCGCTTCTCC

CATAATCTTGCACATAAAATCCTTATCTGACCTGCCACCATCTTCGCAAC

CAAATATGATGTCTTTGAAGCCTAAGCTTTTAGCGTACTTAACACCGCTC

ACGGCCATCTCGATCACTTCTTCTTTAGTCTTCTTCAACTTATATTTCAT

GTGAATGTCACTAGTAGATGTGAATACGAGTACTGTTTGCCTCTTTGCGT

ACTTCAACGCCTCCCAAGCCGCTTCGATATCTCTATGTTTGCATCGTGCG

ATGGCAGATATCACCGGGACGTAACCTGTTTTTTCATCCACCTCGTTCCC

CACGGTCTTGGCGATGGTTTTAACGATTTCGAACTCTTCCTCAGAAGACC

CCGGAAAACCGACTTCCATGATGTCTACTCGGAGTTTAGCGAGCTGCCGG

GCAATCTTAAACTTCTGCGCCGGAGTAAGAGCTCCACCGGGAGCTTGTTC

CCCGTCACGGAGCGTCG

> T_Unigene_BMK.23806 gi|38567380|emb|CAD31141.1| 0 gi|38567380|emb|CAD31141.1| methylthioalkylmalate synthase [Arabidopsis thaliana]

TTGGGATCACAATTCAAAACAAGTTCCTCACATCCTCCAAATCAATATCCAAGTCTCTCTCTCTATCTCTCCTACGTACTCCATAGTAATGGCTTCTTCACTTTTGACATCCTCCGGTGTGATCCCAACCACCGGTTCCACCGTGGTTGTCCGGTCAGTATTACCCGTTGGATCTTCCCTGCCCTCTCTCTGCCTCAACCGTCCGTACAACAAGCCGACCTTGGTCATCTCATGTTGTTCCTCTATGTCCAAAGAAGTGGCAACTAGTACTACTGACCTCAAACCCGTCGTGGAACGGTGGCCGGAGTACATTCCAAACAAGCTCCCCGACAAGAACTATGTGCGTGTACTCGACACGACGCTCCGTGACGGGGAACAAGCTCCCGGTGGAGCTCTTACTCCGGCGCAGAAGTTTAAGATTGCCCGGCAGCTCGCTAAACTCCGAGTAGACATCATGGAAGTCGGTTTTCCGGGGTCTTCTGAGGAAGAGTTCGAAATCGTTAAAACCATCGCCAAGACCGTTGGGAACGAGGTGGATGAAAAAACAGGTTACGTCCCGGTGATATCTGCCATCGCACGATGCAAACATAGAGATATCGAAGCGGCTTGGGAGGCGTTGAAGTACGCAAAGAGGCAAACAGTACTCGTATTCACATCTACTAGTGACATTCACATGAAATATAAGTTGAAGAAGACTAAAGAAGAAGTGATCGAGATGGCCGTGAGCGGTGTTAAGTACGCTAAAAGCTTAGGCTTCAAAGACATCATATTTGGTTGCGAAGATGGTGGCAGGTCAGATAAGGATTTTATGTGCAAGATTATGGGAGAAGCGATAAAAGTGGGTGCAACGGCGGTGAACATCGGGGACACGGTAGGGTACAACGTGCCGCACGAATTCGGAGAACTCATATCCTATCTCAAAGCAAACACTCCTGGAATTGATGATGTTGTCTTCAGTATTCATTGTCACAACGACCTTGGCCTTGCTACCGCTAACACACTCGCCGGTATATGTGCGGGAGCAAGACAAGTCGACGTAACGGTCAATGGAATAGGTGAAAGAAGTGGGAATGCGCCACTTGAAGAGGTTGTCATGGCTTTGAAATGTCGAGGAGCATATGCTATGGATGGTGTTTACACAAATATAGACACACGCCAAATTATGGCTACTAGCCAGATGGTTCAAGAGCTAGGCGGCTTGTATGTTCAAGCGTATAAGCCCATTGTTGGAGCCAACTGTTTTGTTCATGAGAGTGGCATTCACCAGGATGGAATACTGAAAAATCGGAGTACATATGAGATCTTATCACCAGAAGATATTGGGGTTTCAAAATCTCAAAAGTCTGGAATTGTTCTTGGAAAGCTTAGTGGGCGTCATGCTGTGAAAGATCGGCTGAAAGAGTTGGGATATGAAATCGATGAAGAGAAATTGAACAACATCTTCTCACGATTCAGGAATTTAACCAAAGAGAAAAAGAGAATCACAGATGCTGATCTGAAGGCATTAGTGGTGTGCTGTGATGAACTTTCATCAGGGAAACTTAATGGTAAGGGAACTAACGGCTCTGTATCAATCCCCCAGATTCCGTCTGTTGTATAAATTTGTGAACACTTTTGTGTGTTTGTGTAATACGATGATATCAAGTTCCATTTGATATCCATGTGTTTGCTGTTGTAACTTGTAACGAATAAAAACAGTATATAATAATTGAGGTGTGTGTTTTCTATATATTTAACTATTTTTTATAACCAGATGGGA

**IPMI LSU1**

>CL15125.Contig1_All 2 712 3-isopropylmalate dehydratase [Arabidopsis thaliana] >gi|75249794|sp|Q94AR8.1|LEUC_ARATH RecName: Full=3-isopropylmalate dehydratase; AltName: Full=Isopropylmalate isomerase large subunit 1; Short=AtIIL1; Flags: Precursor >gi|332657878|gb|AEE83278.1| 3-isopropylmalate dehydratase [Arabidopsis thaliana] GAGTTCAGTGGTACAACTATCGAAAGTCTGAGTATGGAAGAAAGAATGACATTGTGCAAC

ATGGTTGTGGAAGCTGGGGGAAAGAATGGTGTCATCCCTCCTGATGCGACGACATTAAAT

TATGTTGAGAATAGAACATCTGTACCGTTTGAGCCAGTATACAGCGATGGAAATGCAAGC

TTTGTGGCAGATTATAGATTTGATGTGTCAAAGCTGGAGCCTGTGGTGGCTAAGCCTCAT

TCTCCTGACAACCGGGCTCTAGCAAGAGAATGCAAAGATGTGAAAATTGACAGAGTATAC

ATCGGTTCTTGTACTGGTGGGAAGACAGAAGATTTCGTGGCTGCAGCTAAGCTTTTACAT

GCAGCAGGAAGAAAGGTCAAAGTCCCAACCTTCCTCGTCCCGGCTACTCAGAAGGTATGG

ATGGATGTGTATGCTCTCCCGGTGCCTGGAGCAGGTGGAAAGACATGTGCACAGATATTT

GAAGAAGCTGGATGCGACACACCAACCAGTCCTAGCTGTGGTGCTTGCCTTGGTGGCCCA

GCAGACACCTACGCTCGTTTAAACGAACCTCAAGTGTGTGTCTCGACTACGAACAGGAAC

TTCCCAGGTCGGATGGGTCACAAAGAAGGACAGATTTACTTAGCTTCTCCTTACACAGCT

GCAGCCTCGGCTTTAACCGGTTTTGTCACTGACCCAAGAGAGTTCTTGCAG

>CL71.Contig3_All 136 960 minus strand 3-isopropylmalate dehydratase [Arabidopsis thaliana] >gi|75249794|sp|Q94AR8.1|LEUC_ARATH RecName: Full=3-isopropylmalate dehydratase; AltName: Full=Isopropylmalate isomerase large subunit 1; Short=AtIIL1; Flags: Precursor >gi|332657878|gb|AEE83278.1| 3-isopropylmalate dehydratase [Arabidopsis thaliana]

TCCTCAGTCATCTCTTCATCTCCTTTCGTCTGCAAATCCTCCTCCTCTAAGAAGGATTTT

GGAATTTCTTCGTTTCCTACTAAATCGTCACAGATTTCGATTCATCGATGTCAGAAGAAA

TCGATTTCGAGGAAGATTGTATCTGTAATGGCTCCTCAGAAGGATCGGTCTCCAGGGACT

ACAGGATCGGTGAAAACTGGAATGACTATGACGGAGAAGATTCTAGCTAGAGCTTCTGAG

AAGTCACTAGTGGTTCCTGGTGATAATATTTGGGTTAACGTTGATGTTCTTATGACTCAT

GATGTTTGTGGACCTGGTGCTTTTGGTATTTTCCAGAGAGAGTTTGGTCAAAAAGCTAAG

GTTTGGGATCCAGAGAAGATTGTTGTTATTCCAGACCATTACATATTCACAGCTGATAAG

CGTGCGAATCGCAATGTGGATATTATGCGGGAACATTGCAGGGAACAGAACATTAAGTAT

TTCTATGATATCACTGACCTTGGAGATTTTCGGGCTAATCCTGACTACAAAGGTGTTTGC

CATGTTGCACTTGCACAAGAAGGTCATTGCAGACCAGGAGAGGTTTTGTTAGGAACAGAC

TCACACACCTGTACTGCTGGAGCATTTGGTCAATTTGCTACAGGGATTGGAAACACTGAT

GCAGGTTTTGTATTGGGCACTGGAAAAATCCTCCTTAAGGTTCCACCGACAATGAGGTTT

GTCTTGGATGGTGAAATGCCCAGTTATTTGCAAGCGAAGGATCTGATTTTACAAATCATT

GGTGAAATAACTGTTGCTGGTGCAACTTACAAGACGATGGAGTTC

T_Unigene_BMK.12889 gi|18414006|ref|NP_567405.1| 0 gi|18414006|ref|NP_567405.1| IIL1 (ISOPROPYL MALATE ISOMERASE LARGE SUBUNIT 1); 4 iron, 4 sulfur cluster binding / hydro-lyase/ lyase [Arabidopsis thaliana] TACTATACAAAGAGGAGTTTTTATAACCTTAGGTTTAACACACGCCCTCAAATCTTTTACAACACCGCGCGCACACACACAGAAGAAGAAGCAGAGGAAGAAGAAACCATAACCATGGCTTCCTCAGTCATCTCTTCATCTCCTTTCGTCTGCAAATCCTCCTCCTCTAAGAAGGATTTTGGAATTTCTTCGTTTCCTACTAAATCGTCACAGATTTCGATTCATCGATGTCAGAAGAAATCGATTTCGAGGAAGATCGTCTCCGTTATGGCTCCTCAGAAGGATCGGTCTCCAGGGACTACAGGATCGGTGAAAACTGGAATGACTATGACGGAGAAGATTCTAGCTAGAGCTTCTGAGAAGTCACTAGTGGTTCCTGGTGATAATATTTGGGTTAACGTTGATGTTCTTATGACTCATGATGTTTGTGGACCTGGTGCTTTTGGTATTTTCCAGAGAGAGTTTGGTCAAAAAGCTAAGGTTTGGGATCCAGAGAAGATTGTTGTTATTCCAGACCATTACATATTCACAGCTGATAAGCGTGCGAATCGCAATGTGGATATTATGCGGGAACATTGCAGAGAACAGAATATTAAGTATTTCTATGATATCACTGACCTTGGAGATTTCCGGGCTAACCCTGACTACAAAGGTGTTTGCCATGTTGCACTTGCACAAGAAGGTCATTGCAGACCAGGAGAGGTTTTGTTAGGAACAGACTCACACACTTGTACTGCTGGAGCATTTGGTCAATTTGCTACAGGGATTGGAAACACTGATGCAGGTTTTGTGTTGGGCACTGGAAAAATCCTCCTTAAGGTTCCACCGACAATGAGGTTTGTCTTGGATGGTGAAATGCCCAGTTATTTGCAAGCAAAGGATCTGATTTTACAAATCATTGGTGAAATAACTGTTGCTGGTGCAACTTACAAGACGATGGAGTTCAGTGGTACAACTATCGAAAGTCTGAGTATGGAAGAAAGAATGACATTGTGCAACATGGTTGTGGAAGCTGGGGGAAAGAATGGTGTCATCCCTCCTGATGCGACGACATTAAATTATGTTGAGAATAGAACATCTGTACCGTTTGAGCCGGTATACAGTGATGGAAATGCAAGCTTTGTGGCAGATTATAGATTTGATGTGTCAAAGCTGGAGCCTGTGGTGGCTAAGCCTCATTCACCTGACAACCGGGCTCTAGCAAGAGAATGCAAAGATGTGAAAATTGACAGAGTATACATCGGTTCTTGTACTGGTGGGAAGACAGAAGATTTCGTGGCTGCAGCTAAGCTTTTACATGCAGCAGGAAGAAAGGTCAAAGTCCCAACCTTCCTCGTCCCGGCTACTCAGAAGGTATGGATGGATGTGTATGCTCTTCCGGTGCCTGGAGCAGGTGGGAAGACATGTGCACAGATATTTGAAGAAGCTGGATGCGACACACCAACCAGTCCTAGCTGTGGTGCTTGCCTTGGTGGCCCAGCAGACACATACGCTCGTTTAAACGAACCTCAAGTGTGTGTCTCGACCACGAACAGGAACTTCCCAGGTCGGATGGGACACAAAGAAGGACAGATTTACTTAGCTTCTCCTTACACAGCTGCAGCCTCGGCTTTAACCGGTTTTGTCACTGACCCAAGAGAGTTCTTGCAG

**IPMI SSU2**

>CL15367.Contig1_All 41 796 aconitase C-terminal domain-containing protein [Arabidopsis lyrata subsp. lyrata] >gi|297325863|gb|EFH56283.1| aconitase C-terminal domain-containing protein [Arabidopsis lyrata subsp. lyrata] ATGGCCTCTTCTCTTCCTACATTGCCCCAAGCCTTACCTTCCTCGTCAATCAAATCGTCT

TCTTCCGGCGTTCCTTTCCGATCTTCTTTCCTCAGAATCAATGGTTCCATTTCCTTCATC

CCCTCATCTATCTCACTCACTCCACGTGGCACATCCCCCACGACCATCATCCCACGTGCT

GCTGCCGCCGATACTAACGAAACGCTAGCTAAAACCACCTTCCATGGTCTCTGCTTCGTG

TTGAAAGACAACATAGACACCGACCAGATCATCCCAGCAGGATTCACTACCATCTTCCCA

TCGAACCAGCGAGAGCGTGACGAGCTCGCTGCTCATGCTCTCTCCGGTCTCCCAAGCTTC

CACAAAACCCGGTTCGTTGAACCAGGAGAGATCAAGTCAAAATATTCAATCATAATCGGC

GGAGAAAACTTCGGCTGTGGATCGTCACGTGAACACGCTCCGGTTTGTCTCGGAGCAGCG

GGAGCTAAAGTCGTTGTAGCTGAGTCTTACGCAAGGATCTTTTTCCGTAACTCGGTGGCT

ACAGGAGAAGTGTTTCCGCTCGAGTCGGAGGTTAGAGTCTGTGACGAGTGTAAGACAGGA

GACACGGTGACGATCGAGCTAAGAGATAGTGGTGGTTTATTAACTAATCACACGACCGGG

AAAAAATATAAGTTGAAGTCGATCGGTGATGCAGGACCGGTTATTAATGCCGGTGGTATT

TTTGCTTATGCGAGGAAGATGGGAATGATTCCATCT

>T_Unigene_BMK.15701 gi|297824281|ref|XP_002880023.1| 3.49101e-163 gi|297824281|ref|XP_002880023.1| aconitase C-terminal domain-containing protein [Arabidopsis lyrata subsp. lyrata] TCTAACCTTCAGAGTAAAGAAACAATGGCCTCTTCTCTTCCTACATTGCCCCAAGCCTTACCTTCCTCGTCAATCAAATCGTCTTCTTCCGGCGCTCCTTTCCGATCTTCTTTCCTCAGAATCAGTGGTTCCACTTCCTTCATCCCCTCATCTATCTCCCTCACTCCACGTGGCACATCCCCCACGACCATAATCCCACGTGCCGCTGCCGCCGATACTAACGAAACGCTAGCTGAAACCACCTTCCATGGTCTCTGCTTCGTCTTGAAAGACAACATAGACACCGACCAGATCATCCCAGCAGGATTCACTACCATCTTCCCATCGAACCAGCGAGAGCGTGACGAGCTCGCTGCTCATGCTCTCTCCGGTCTCCCAAGCTTCCACAAAACCCGGTTCGTTGAACCAGGAGAGATCAAGTCAAAATATTCAATCATAATCGGCGGAGAAAACTTCGGTTGTGGATCGTCACGTGAACACGCTCCGGTTTGTCTCGGAGCAGCGGGAGCTAAAGTCGTTGTAGCTGAGTCTTACGCAAGGATCTTTTTCCGTAACTCGGTGGCTACAGGAGAAGTGTTTCCGCTCGAGTCAGAGGTTAGAGTCTGTGACGAGTGTAAGACAGGAGACACGGTGACGATCGAGCTAAGAGATAGTGGTGGTTTATTAACTAATCACACGACCGGGAAAAAATATAAGTTGAAGTCGATCGGTGATGCAGGACCGGTTATTAATGCCGGTGGTATTTTTGCTTATGCGAGGAAGATGGGAATGATTCCATCTCCATCATTAGCT

**IPMI SSU3**

>CL8261.Contig1_All 61 819 isopropylmalate isomerase 1 [Arabidopsis thaliana] >gi|7529742|emb|CAB86927.1| 3-isopropylmalate dehydratase-like protein (small subunit) [Arabidopsis thaliana] >gi|332646336|gb|AEE79857.1| isopropylmalate isomerase 1 [Arabidopsis thaliana]

ATGGCGACTTCTCTACAATTTCTAAACCCTACACTGTTCAGATCCTTAGCTTCCTCAAAC

AAAAACTCATCCTCTCTCTCGCAATCTCCTTTCTTCCGACTCAATTCCACCTCCACCGCA

TTCAATTTCAAACCCCTGACTTCCTCCTCCGCCAAGATCACCACACGCGCCGCCGCATCT

TCCTCCGATTCAGGCGATCCGCAAGCTAGAGAGACTTTCCACGGTCTCTGCTTTGTCGTG

AAAGACAACATCGACACAGATCAAATCATACCCTCCCAGTACGGCACTCTCATCCCCTCG

AGTCCAGAAGATCGCCAGAAGCTCGGCTCGTTCGCGCTGATCGGACTACCAAGTTTCTAC

AATAATCGTTTCGTGGAGCCAGGAGAGATGAAATCAAAGTACTCCGTCATCATCGCCGGC

GATAATTTCGGCTGTGGATCCTCTCGCGAACACGCTCCGGTCTGCCTCGGTGCTGCGGGA

GCTAAAGCCGTGGTGGCGGAATCGTACGCCAGGATCTTCTTCCGGAACTGTGTAGCCACC

GGAGAGATCTTCCCGTTGGAATCGGAGGTTAGGATTTGCGATGAGTGTAAGACAGGGGAT

GTGGTGACCATCGAGCCCAACGAAGACGGTAGTAGTTTGCTGATTAATCATACGACGAGG

AAGGAATACAAATTAAAACCGCTCGGTGACGCTGGTCCGGTCATCGACGCCGGCGGAATC

TTCGCTTATGCTAGAAAAGCCGGCATGATTCCTTCATCT

> CK_Unigene_BMK.21560 gi|15231608|ref|NP_191458.1| 6.70885e-158 gi|15231608|ref|NP_191458.1| aconitase C-terminal domain-containing protein [Arabidopsis thaliana]

ACAACAAGCAAAACAATGGCGACTTCTCTACAGTTTCTAAACCCTACACTGTTCAGATCCTTAGCTTCCTCAAACAAAAACTCATCCTCTCTCTCGCCTTCTCCTTTCTTCCGACTCAATTCCACCTCCACCGCATTCAATTTCAAACCACTGACTTCCTCCTCCGCCAAGATCACCACACGCGCCGCCGCATCTTCCTCCGATTCAGACGATTCGCAAGCTAGAGAGACTTTCCACGGCCTCTGCTTTGTCGTGAAAGACAACATCGACACAGATCAAATCATACCCGCCCAGTACGGCACTCTCATCCCCTCGAGTCCAGAAGATCGCCAGAAGCTCGGCTCGTTCGCGCTAATTGGACTACCAAGTTTCTACAATAACCGTTTCGTGGATCCAGGAGAGATGAAATCAAAGTACTCCGTCATCATCGCCGGCGATAATTTCGGCTGTGGATCCTCTCGCGAACACGCTCCGGTCTGCCTCGGTGCTGCGGGAGCTAAAGCCGTGGTGGCGGAATCGTACGCCAGGATCTTCTTCCGGAACTGTGTAGCCACCGGAGAGATCTTCCCGTTGGAATCGGAGGTTAGGATTTGTGATGAGTGCAAGACAGGGGATGTGGTGACCATCGAGCCCAAGGAAGACGGTAGTAGTTTGCTGATTAATCATACGACGAGGAAGGAATACAAATTAAAACCGCTCGGTGACGCTGGTCCGGTCATCGACGCCGGCGGAATCTTCGCTTATGCTAGAAAAGCCGGCATGATTCCTTCTGCATCATCATCTACT

**IPMDH1**

>CL3445.Contig3_All 29 1243 3-isopropylmalate dehydrogenase 3 [Arabidopsis thaliana] >gi|21759264|sp|Q9FMT1.1|LEU33_ARATH RecName: Full=3-isopropylmalate dehydrogenase 3, chloroplastic; Short=3-IPM-DH 3; Short=IMDH 3; AltName: Full=Beta-IPM dehydrogenase 3; Flags: Precursor >gi|9757801|dbj|BAB08299.1| 3-isopropylmalate dehydrogenase [Arabidopsis thaliana] >gi|53749178|gb|AAU90074.1| At5g14200 [Arabidopsis thaliana] >gi|59958306|gb|AAX12863.1| At5g14200 [Arabidopsis thaliana] >gi|332004614|gb|AED91997.1| 3-isopropylmalate dehydrogenase 3 [Arabidopsis thaliana]

ATGGCGGCGGTTTCGCAAACGAACATCCGGCTCAATTCGAGCAAGATCTTCCCGGGAAAA

TACAGTCCTTTAACCGATCATCAGTCTCGAATAAGGTGCGCCGCCGCTTCACCAGGTAAA

AAAAGGTTCAACATCGCACTCCTTGCCGGCGATGGCATCGGTCCAGAAGTTATCTCCGTT

GCCAAGAAGGTGCTTCAGCAAGCTGGATCTCTCGAAGGAGTTGAATTTAGCTTCCGGGAG

ATGCCCATTGGAGGAGCTGCTTTGGATTTGGTTGGAGTGCCCTTGCCTGAAGAGACCATC

TCCGCTGCTAAAGAATCTGATGCTGTGCTTCTTGGAGCCATCGGAGGGTACAAATGGGAT

AACAATGAAAAACATCTAAGGCCTGAGAAGGGTTTACTTCAGCTTCGTGCTGGTCTCAAA

GTCTTTGCTAATCTCAGACCTGCTACAGTCCTCCCACAGTTAGTCGATGCTTCTACCTTG

AAGAGAGAGGTAGCAGAAGGTGTTGATCTTATGGTTGTAAGGGAGCTTACAGGAGGTATT

TACTTTGGAGAGCCAAGAGGCATTAAGACTAATGAAAATGGCGAAGAAGTCGGCTTTAGT

ACAGAGATCTATACTGCTCACGAGATTGATAGAATTGCTCGTGTTGCATTCGAGACTGCT

AGGAAAAGGCGTGGCAAGCTCTGTTCTGTCGACAAAGCCAATGTGTTGGATGCATCAATA

TTGTGGAGGAAAAGAGTAACAGCGTTAGCCTCTGAGTATCCAGATGTTGAACTATCACAT

ATGTATGTCGATAATGCTGCAATGCAGCTTATTCGTGACCCTAAACAGTTTGATACAATC

CTCACCAATAACATATTTGGTGATATATTATCTGATGAAGCCTCAATGATCACTGGAAGC

ATTGGAATGCTTCCATCTGCAAGTCTCGGTGAATCGGGACCTGGACTCTTTGAACCTATA

CATGGCTCTGCACCAGATATAGCTGGGCAAGACAAGGCAAATCCATTGGCTACCATTCTC

AGCGCAGCGATGCTTCTGAAATATGGACTTGGAGAAGAAAAAGCTGCAAAGAGGATTGAA

GACGCTGTTGTGGATGCTCTGAACAAAGGGTTTAGAACCGGAGACATCTACTCTCCCGGA

AATAAACTGGTGGGATGCAAGGAGATGGGTGAGGAAGTGCTCAAATCAGTGGACTCCAAA

GTTCCAGCTACTGTT

>CL3445.Contig1_All 3 989 3-isopropylmalate dehydrogenase 3 [Arabidopsis thaliana] >gi|21759264|sp|Q9FMT1.1|LEU33_ARATH RecName: Full=3-isopropylmalate dehydrogenase 3, chloroplastic; Short=3-IPM-DH 3; Short=IMDH 3; AltName: Full=Beta-IPM dehydrogenase 3; Flags: Precursor >gi|9757801|dbj|BAB08299.1| 3-isopropylmalate dehydrogenase [Arabidopsis thaliana] >gi|53749178|gb|AAU90074.1| At5g14200 [Arabidopsis thaliana] >gi|59958306|gb|AAX12863.1| At5g14200 [Arabidopsis thaliana] >gi|332004614|gb|AED91997.1| 3-isopropylmalate dehydrogenase 3 [Arabidopsis thaliana]

AATTTCCAGGAGATGCCTGTCGGAGGAGCAGCCTTGGATTTGGTCGGAGTTCCTTTGCCG

GAAGAAAGCTTTAAAGCTGCAAAAGAATCTGATGCTATTCTTCTTGGAGCTATCGGAGGG

TACAAATGGGACAAGAATGAGAAACATATGAGACCAGAGATGGCTCTGTTTTATCTTCGA

AGAGATCTCAAAGTCTTTGCTAATTTGAGACCTGCTACAGTTTTGCCACAGCTTGTTGAT

GCTTCTACCTTGAAGAGAGAAGTGGCAGAAGGTGTTGATATGATGATTGTAAGGGAGCTT

ACAGGAGGTATTTACTTTGGAGAGCCAAGGGGCATTAAGACCAATGAAAATGGCGAGGAA

GTTGGCTTTAATACTGAGGTTTATGCTGCTCACGAGATTGACAGAATTGCTCGTGTTGCC

TTTGAGACTGCTCGGAAACGTCGTGGCAAGCTGTGCTCTGTCGACAAAGCTAATGTCCTT

GATGCCTCAATCTTATGGAGGAAACGAGTAACAGCATTAGCCTCTGAATATCCTGATGTT

GAGCTGTCACATATGTATGTTGACAATGCCGCAATGCAGCTTGTTCGTGACCCTAAACAG

TTTGATACAATCCTCACCAATAACATATTTGGTGATATATTATCTGATGAAGCCTCAATG

ATCACTGGAAGCATTGGAATGCTTCCATCTGCAAGTCTCGGTGAATCGGGACCTGGACTC

TTTGAACCTATACATGGCTCTGCACCAGATATAGCTGGGCAAGACAAGGCAAATCCATTG

GCTACCATTCTCAGCGCAGCGATGCTTCTGAAATATGGACTTGGAGAAGAAAAAGCTGCA

AAGAGGATTGAAGACGCTGTTGTGGATGCTCTGAACAAAGGGTTTAGAACCGGAGACATC

TACTCTCCCGGAAATAAACTGGTGGGATGCAAGGAGATGGGTGAGGAAGTGCTCAAATCA

GTGGACTCCAAAGTTCCAGCTACTGTT

>CL3445.Contig2_All 52 1266 3-isopropylmalate dehydrogenase 2 [Arabidopsis thaliana] >gi|21759255|sp|P93832.1|LEU32_ARATH RecName: Full=3-isopropylmalate dehydrogenase 2, chloroplastic; Short=3-IPM-DH 2; Short=IMDH 2; AltName: Full=Beta-IPM dehydrogenase 2; Flags: Precursor >gi|340708011|pdb|3R8W|A Chain A, Structure Of 3-Isopropylmalate Dehydrogenase Isoform 2 From Arabidopsis Thaliana At 2.2 Angstrom Resolution >gi|340708012|pdb|3R8W|B Chain B, Structure Of 3-Isopropylmalate Dehydrogenase Isoform 2 From Arabidopsis Thaliana At 2.2 Angstrom Resolution >gi|340708013|pdb|3R8W|C Chain C, Structure Of 3-Isopropylmalate Dehydrogenase Isoform 2 From Arabidopsis Thaliana At 2.2 Angstrom Resolution >gi|340708014|pdb|3R8W|D Chain D, Structure Of 3-Isopropylmalate Dehydrogenase Isoform 2 From Arabidopsis Thaliana At 2.2 Angstrom Resolution >gi|332198299|gb|AEE36420.1| 3-isopropylmalate dehydrogenase 2 [Arabidopsis thaliana]

ATGGCGGCGGCTCTACAAACCAATATCCGACCTGTCAAGTTTCCGGCAACGTTCAGAGCT

TTCACCAAACAATCTTTGACACCCTTTAGAGTGAGATGCGCTGTTGCTTCCCCTGGGAAA

AAACGATACAACATCACTCTCCTTCCCGGTGACGGTATCGGTCCAGAGGTCATCTCTATT

GCCAAAAATGTGCTACAGCAAGCTGGATCTCTCGAAGGAGTTGAATTTAGCTTCCGGGAG

ATGCCCATTGGAGGAGCTGCTTTGGATTTGGTTGGAGTGCCCTTGCCTGAAGAGACCATC

TCCGCTGCTAAAGAATCTGATGCTGTGCTTCTTGGAGCCATCGGAGGGTACAAATGGGAT

AACAATGAAAAACATCTAAGGCCTGAGAAGGGTTTACTTCAGCTTCGTGCTGGTCTCAAA

GTCTTTGCTAATCTCAGACCTGCTACAGTCCTCCCACAGTTAGTCGATGCTTCTACCTTG

AAGAGAGAGGTAGCAGAAGGTGTTGATCTTATGGTTGTAAGGGAGCTTACAGGAGGTATT

TACTTTGGAGAGCCAAGAGGCATTAAGACTAATGAAAATGGCGAAGAAGTCGGCTTTAGT

ACAGAGATCTATACTGCTCACGAGATTGATAGAATTGCTCGTGTTGCATTCGAGACTGCT

AGGAAAAGGCGTGGCAAGCTCTGTTCTGTCGACAAAGCCAATGTGTTGGATGCATCAATA

TTGTGGAGGAAAAGAGTAACAGCGTTAGCCTCTGAGTATCCAGATGTTGAACTATCACAT

ATGTATGTCGATAATGCTGCAATGCAGCTTATTCGTGACCCTAAACAGTTTGATACAATC

CTCACCAATAACATATTTGGTGATATATTATCTGATGAAGCCTCAATGATCACTGGAAGC

ATTGGAATGCTTCCATCTGCAAGTCTCGGTGAATCGGGACCTGGACTCTTTGAACCTATA

CATGGCTCTGCACCAGATATAGCTGGGCAAGACAAGGCAAATCCATTGGCTACCATTCTC

AGCGCAGCGATGCTTCTGAAATATGGACTTGGAGAAGAAAAAGCTGCAAAGAGGATTGAA

GACGCTGTTGTGGATGCTCTGAACAAAGGGTTTAGAACCGGAGACATCTACTCTCCCGGA

AATAAACTGGTGGGATGCAAGGAGATGGGTGAGGAAGTGCTCAAATCAGTGGACTCCAAA

GTTCCAGCTACTGTT

>CL20774.Contig1_All 57 1271 3-isopropylmalate dehydrogenase 2 [Arabidopsis thaliana] >gi|21759255|sp|P93832.1|LEU32_ARATH RecName: Full=3-isopropylmalate dehydrogenase 2, chloroplastic; Short=3-IPM-DH 2; Short=IMDH 2; AltName: Full=Beta-IPM dehydrogenase 2; Flags: Precursor >gi|340708011|pdb|3R8W|A Chain A, Structure Of 3-Isopropylmalate Dehydrogenase Isoform 2 From Arabidopsis Thaliana At 2.2 Angstrom Resolution >gi|340708012|pdb|3R8W|B Chain B, Structure Of 3-Isopropylmalate Dehydrogenase Isoform 2 From Arabidopsis Thaliana At 2.2 Angstrom Resolution >gi|340708013|pdb|3R8W|C Chain C, Structure Of 3-Isopropylmalate Dehydrogenase Isoform 2 From Arabidopsis Thaliana At 2.2 Angstrom Resolution >gi|340708014|pdb|3R8W|D Chain D, Structure Of 3-Isopropylmalate Dehydrogenase Isoform 2 From Arabidopsis Thaliana At 2.2 Angstrom Resolution >gi|332198299|gb|AEE36420.1| 3-isopropylmalate dehydrogenase 2 [Arabidopsis thaliana]

ATGGCGGCGGCTCTACAAACCAATATCCGACCTGTCAAGTTTCCGGCAACGTTCAGAGCT

TTCACCAAACAATCTTTGACACCCTTTAGAGTGAGATGCGCTGTTGCTTCCCCTGGGAAA

AAACGATACAACATCACTCTCCTTCCCGGTGACGGTATCGGTCCAGAGGTCATCTCTATT

GCCAAAAATGTGCTACAGCAAGCTGGATCTCTCGAAGGAGTTGAATTTAGCTTCCGGGAG

ATGCCCATTGGAGGAGCTGCTTTGGATTTGGTTGGAGTGCCCTTGCCTGAAGAGACCATC

TCCGCTGCTAAAGAATCTGATGCTGTGCTTCTTGGAGCCATCGGAGGGTACAAATGGGAT

AACAATGAAAAACATCTAAGGCCTGAGAAGGGTTTACTTCAGCTTCGTGCTGGTCTCAAA

GTCTTTGCTAATCTCAGACCTGCTACAGTCCTCCCACAGTTAGTCGATGCTTCTACCTTG

AAGAGAGAGGTAGCAGAAGGTGTTGATCTTATGGTTGTAAGGGAGCTTACAGGAGGTATT

TACTTTGGAGAGCCAAGAGGCATTAAGACTAATGAAAATGGCGAAGAAGTCGGCTTTAGT

ACAGAGATCTATACTGCTCACGAGATTGATAGAATTGCTCGTGTTGCATTCGAGACTGCT

AGGAAAAGGCGTGGCAAGCTCTGTTCTGTCGACAAAGCCAATGTGTTGGATGCATCAATA

TTGTGGAGGAAAAGAGTAACAGCGTTAGCCTCTGAGTATCCAGATGTTGAACTATCACAT

ATGTATGTCGATAATGCTGCAATGCAGCTTATTCGTGACCCTAAACAGTTTGATACAATC

GTCACAAACAACATTTTTGGTGATATATTATCCGATGAAGCTTCAATGATAACAGGAAGC

ATAGGAATGCTTCCATCTGCTAGTCTCAGTGATTCGGGACCTGGACTCTTTGAACCTATA

CATGGTTCTGCACCTGATATTGCTGGACAGGATAAGGCAAACCCATTGGCAACCATCCTC

AGCGCTGCGATGCTTCTGAAATATGGACTTGGAGAAGAAAAGGCAGCTAAGAGAATTGAA

GACGCAGTGTTGGTTGCTCTGAACAAAGGGTTCAGAACCGGTGACATTTACTCTGCGGGA

ACTAAACTGGTGGGCTGCAAGGAGATGGGAGAGGAGGTTCTGAAGTCGGTGGACTCCCAA

ATTCCAGCTTCTGTT

>T_Unigene_BMK.16254 gi|15241338|ref|NP_196924.1| 0 gi|15241338|ref|NP_196924.1| 3-isopropylmalate dehydrogenase, chloroplast, putative [Arabidopsis thaliana]

TCCGACTCCAAATTAATGGCGGCGGTTTCGCAAACGAACATCCGGCTCAATTCGAGCAAGATCTTCCCGGGAAAATACAGTCCTTTAACCGATCATCAGTCTCGAATAAGGTGCGCCGCCGCTTCACCAGGTAAAAAAAGGTTCAACATCGCACTCCTTGCCGGCGATGGCATCGGTCCAGAAGTTATCTCCGTTGCCAAGAATGTGCTTCAGCAAGCTGGATCTCTCGAAGGACTGGAGTTCAATTTCCAGGAGATGCCTGTCGGAGGAGCAGCCTTGGATTTGGTCGGAGTTCCTTTGCCGGAAGAAAGCTTTAAAGCTGCAAAAGAATCTGATGCTATTCTTCTTGGAGCTATCGGAGGGTACAAATGGGACAAGAATGAGAAACATATGAGACCAGAGATGGCTCTCTTTTATCTTCGAAGAGATCTCAAAGTCTTTGCTAATTTGAGACCTGCTACAGTTTTGCCACAGCTAGTTGATGCTTCTACCTTGAAGAGAGAAGTGGCAGAAGGTGTTGATATGATGATTGTAAGGGAGCTTACAGGAGGTATTTACTTTGGAGAGCCAAGAGGCATTAAGACCAATGATAATGGCGAAGAAGTCGGCTTTAGTACAGAGATCTACACTGCTCACGAGATTGATAGAATTGCTCGTGTTGCATTCGAGACTGCTAGGAAAAGGCGTGGCAAGCTCTGTTCTGTAGACAAAGCCAATGTGTTGGATGCATCAATATTGTGGAGGAAAAGAGTAACAGCGTTAGCCTCTGAGTATCCAGATGTTGAACTATCACATATGTATGTCGATAATGCTGCAATGCAGCTTATTCGTGACCCTAAACAGTTTGATACAATCCTCACCAATAACATATTTGGTGATATATTATCTGATGAAGCCTCAATGATCACTGGAAGCATTGGAATGCTTCCATCTGCAAGTCTCGGTGAATCGGGACCTGGACTCTTTGAACCTATACATGGCTCTGCACCAGATATAGCTGGGCAAGACAAGGCAAATCCATTGGCTACCATTCTCAGCGCAGCGATGCTTCTGAAATATGGACTTGGAGAAGAAAAAGCTGCAAAGAGGATTGAAGACGCTGTTGTGGATGCTCTGAACAAAGGGTTTAGAACCGGAGACATCTACTCTCCCGGGAATAAACTGGTGGGATGCAAGGAGATGGGTGAGGAAGTGCTCAAATCAGTGGACTCCAAAGTTCCAGCTACTGTT

> CK_Unigene_BMK.12842 gi|15220167|ref|NP_178171.1| 0 gi|15220167|ref|NP_178171.1| 3-isopropylmalate dehydrogenase, chloroplast, putative [Arabidopsis thaliana] GGTGAACGTTGCCGGAAACAATCCTTTGTACCTGAAATGGCGGCGGCTCTGCAGACGAATATCCGACCAGTTAAGTTTCCGGCAACGTTCACCAAACAATCTTTAGCACCCTTTAGAGTGAGATGCGCTGTTGCTTCCCCTGGGAAAAAACGATACAACATCACTCTCCTTCCCGGTGACGGTATCGGTCCAGAGGTTATCTCTATTGCCAAAAATGTGCTACAGCAAGCTGGATCTCTCGAAGGAGTTGAATTTAGCTTCCGGGAGATGCCCATTGGAGGAGCTGCTTTGGATTTGGTAGGAGTGCCTTTGCCTGAAGAGACCATCTCCGCTGCTAAAGAATCTGATGCTGTGCTTCTTGGAGCCATCGGAGGGTACAAATGGGATAACAATGAAAAACATCTAAGGCCTGAGAAGGGTTTACTTCAGCTTCGTGCTGGTCTCAAAGTCTTTGCTAATCTCAGACCTGCTACAGTTCTCCCACAGTTAGTCGATGCTTCTACCTTGAAGAGAGAGGTAGCAGAAGGTGTTGATCTTATGGTTGTAAGGGAGCTTACAGGAGGTATTTACTTTGGAGAGCCTAGGGGCATTAAGACCAATGAAAATGGCGAGGAAGTTGGCTTTAATACTGAGGTTTATGCTGCTCACGAGATTGACAGAATTGCTCGTGTTGCCTTTGAGACTGCTCGGAAACGTCGTGGCAAGCTGTGCTCTGTCGACAAAGCTAATGTCCTTGATGCCTCAATCTTATGGAGGAAACGAGTAACAGCATTAGCCTCTGAATATCCTGATGTTGAGCTGTCACATATGTATGTTGACAATGCCGCAATGCAGCTTGTTCGTGACCCTAAACAGTTTGATACAATCGTCACAAACAACATTTTTGGTGATATATTATCCGATGAAGCTTCAATGATAACAGGAAGCATAGGAATGCTTCCATCTGCTAGTCTCAGTGATTCGGGACCTGGACTCTTTGAACCTATACATGGCTCTGCACCAGATATAGCTGGGCAAGACAAGGCAAATCCATTGGCTACCATTCTCAGCGCAGCGATGCTTCTGAAATATGGACTTGGAGAAGAAAAAGCTGCAAAGAGGATTGAAGACGCGGTTGTGGATGCTCTCAACAAAGGGTTTAGAACCGGAGACATCTACTCTCCCGGAAATAAACTGGTGGGATGCAAGGAGATGGGTGAGGAAGTGCTCAAATCAGTGGACTCCAAAGTTCCAGCTACTGTT

**BCAT-3**

>CL201.Contig5_All 731 1978 ATBCAT-3 [Arabidopsis lyrata subsp. lyrata] >gi|297321828|gb|EFH52249.1| ATBCAT-3 [Arabidopsis lyrata subsp. lyrata]

ATGGAGAGAGCTGCAATTTCACCAAGTATTAATCAAAATTACCTACTTTCTCCTTCACGC

GCCTTCCCCACGCGCCTCCACTCTTTCTCCTCTACTCGGAACTTATCACCATCGTCTTCC

TCCATAAAGCTTCAGCATTCTTCGTCTTCTTCCTCTGTATTGTCCAGTTCCATCAATGGC

GGAATCTCCACTACTCGATACAACGCTGTTTCTTCAAGAGCTGAGGTATCTGAATTAGCC

GACATTGATTGGGATAACATCGGATTTGGGCTTAAGCCGACTGATTACATGTATGTGATG

AAATGTAATATCGATGGAGAGTTCTCAAATGGTGAATTACAACGTTTTGGGAACATTGAA

GTCAGCCCATCTGCTGGTGTGCTCAACTATGGACAGGGATTGTTCGAAGGGCTAAAAGCG

TACAGAAAGCAAGACGGTAGTAACATCCTCCTCTTTCGTCCCGAAGAGAATGCAATCCGT

ATGAGAAACGGTGCTGAGAGGATGTGTATGCCTGCTCCAACCGTTGAGCAGTTTGTCGAA

GCTGTGAAAAAAACTGTATTAGCTAACAAACGTTGGGTTCCACCTTCAGGTAAAGGTTCC

TTATATGTTAGACCATTGCTAATGGGAACAGGAGCTGTTCTTGGTCTTGCGCCTGCACCA

GAATATACTTTCCTTGTCTATGTTTCGCCTGTCGGGAACTACTTCAAGGAAGGTGTGGCA

CCGATCAATTTGATCGTGGAAAGTGAATTTCACCGTGCAACTCCTGGTGGTACCGGAGGT

GTTAAAACCATCGGAAATTATGCTGCAGTACTCAAGGCACAGTCGATTGCGAAAGCCAAA

GGATATTCCGATGTTTTGTACCTTGATTGCGTTTACAATAGATTTCTTGAGGAGGTCTCG

TCTTGCAATATCTTCATTGTGAAGGACAATGTGATCTCTACTCCTGAAATAAAAGGAACC

ATCTTACCCGGGATTACAAGGAAAAGTATAATCGATGTGGCTCAAACCCAAGGGTTTCAG

GTTGAGGAACGTAATGTGACAGTAGACGAATTAATAGAAGCGGACGAGGTTTTCTGCACA

GGAACCGCTGTGGTTGTCTCTCCCGTTGGAAGCATTACTTACAAAGGCAAAAGAGTGTCA

TATGGAAAAGGTACCTTTGGAACTGTCTCGGAGCAACTCTACACTGTTCTGACAAGCTTG

CAGATGGGTCTGAGTGAGGACAACATGAATTGGACTGTGAATCTGAGT

>CL3170.Contig1_All 117 1352 ATBCAT-5 [Arabidopsis lyrata subsp. lyrata] >gi|297310815|gb|EFH41239.1| ATBCAT-5 [Arabidopsis lyrata subsp. lyrata]

ATGGAGCGAACCGGCGTTATCTCTGGTTTTCATCAAAGCTACATCCTCTCCCCTTCACGC

GCCGCCACATCCACCACGCCCCTCCACTCTTTCTCCTCCTCCCTCAGAAACTATCCATCT

TCCTCTCTCAGGATTCGCCACTCTGCTTCTTCCGTCTCTTTCACTTCCCGTTACCGATGC

GACGCCGTTTCACCCAATTCTTCCAGCAGCACTGATGTGACTGAATTAGCCGAAATTGAT

TGGGACAACATCGACTTTGGGCTTAAACCAACGGACTACATGTACGCCATGAAATGTAGC

CGTGATGGTGATTTCTCTAAAGGTCAATTGCAAAGTTTTGGAAACATTGAAGTCAGCCCT

TCAGCTGCTGTACTCAACTATGGACAAGGTTTGTTTGAAGGTCTAAAAGCTTACAGAAAA

CATGACGGTAATATTCTCCTCTTCCGTCCCGAGGAGAATGCCATCCGAATGAGAAATGGT

GCTGAAAGAATGTGCATGCCTGCTCCATCCGTCGAGCAGTTTGTTGAGGCCGTGAAAACA

ACTGTATTAGCAAACAAACGCTGGATTCCACCTCCAGGTAAAGGATCGTTATACATAAGG

CCATTGCTAATGGGAACTGGAGCTGTTCTTGGTCTTGCCCCTGCTCCCGACTACACTTTC

CTTATCTATGTTTCACCCGTTGGTAACTACTTCAAGGAAGGTGTGGCGCCGATCAACTTG

ATTGTTGAAAATGATTTCCATCGTGCAACTCCCGGCGGTACTGGAGGTGTTAAAACCATC

GGTAATTATGCTGCAGTCTTGAAGGCACAGTCAGCTGCGAAAGCTAAAGGGTACTCGGAT

GTTTTATATCTTGATTGCATTTACAAAAGATATATCGAGGAGGTTTCATCTTGCAACATT

TTCATTGTGAAGGATAATGTGATCTCTACTCCTGAAATTAGAGGAACCATCTTGCCTGGA

ATTACTAGGAAGAGTATAATTGAAGTGGCTCGGAGCCAAGGGTTCAAGGTGGAGGAACGA

AATGTGACAGTGGATGAATTGGTGGAAGCGGATGAAGTTTTCTGCACTGGAACCGCCGTT

GTTTTATCACCGGTGGGAAGCATTACTTACAAAAGCCAAAGGGTTTCTTATGGAGAAGAT

GGATTTGGAACTGTGTCGAAACAACTATACACTTCCTTGACGAGCCTGCAAATGGGTCTG

AGCGAAGATAACATGAACTGGACTGTTCAGTTGAGT

> T_Unigene_BMK.10464 gi|297816214|ref|XP_002875990.1| 0 gi|297816214|ref|XP_002875990.1| ATBCAT-3 [Arabidopsis lyrata subsp. lyrata]

ACGACGCTAAAAAGAAAACGCAGAGAGCCTCCTCCGTCTCCTCCCATTGTCGTCTTATCGAAGAAGAAGAAGAAGCAAACAACACACATTTTGAGAGAAATGGAGAGAGCTGCAATTTCACCAAGTATTAATCAAAATTACCTACTTTCTCCTTCACGCGCCTTCCCCACGCGCCTCCACTCTTTCTCCTCTACTCGGAACTTATCACCATCGTCTTCCTCCATAAAGCTTCAGCATTCTTCGTCTTCTTCCTCTGTATTGTCCAGTTCCATCAATGGCGGAATCTCCACTACTCGATACAACGCTGTTTCTTCAAGAGCTGAGGTGTCTGAATTAGCCGACATTGATTGGGATAACATTGGATTTGGGCTTAAGCCGACTGATTACATGTATGTGATGAAATGTAATATCGATGGAGAGTTCTCAAATGGTGAATTGCAACGTTTTGGGAACATTGAAGTCAGCCCATCTGCCGGTGTGCTCAATTATGGACAGGGATTGTTCGAAGGGCTAAAAGCGTACAGAAAGCAAGACGGTAGTAACATCCTTCTCTTTCGTCCCGAAGAGAATGCAATCCGTATGAGAAACGGTGCTGAGAGGATGTGTATGCCTGCTCCAACCGTTGAGCAGTTTGTCGAAGCTGTGAAAAAAACTGTATTAGCTAACAAACGTTGGGTTCCACCTTCAGGTAAAGGTTCCTTATATGTTAGACCATTGCTAATGGGAACAGGAGCTGTTCTTGGTCTTGCGCCTGCACCAGAATATACTTTCCTTGTCTATGTTTCGCCTGTCGGGAACTACTTCAAGGAAGGTGTGGCACCGATCAATTTGATCGTGGAAAGTGAATTTCACCGTGCAACTCCTGGTGGTACCGGAGGTGTTAAAACGATCGGAAATTATGCTGCAGTACTCAAGGCACAGTCGATTGCGAAAGCCAAAGGATATTCCGATGTTTTGTACCTTGATTGCGTTTACAATAGATTTCTTGAGGAGGTCTCGTCTTGCAATATCTTCATTGTGAAGGACAATGTGATCTCTACTCCTGAAATAAAAGGAACCATCTTACCCGGGATTACAAGGAAAAGTATAATCGATGTGGCTCGAACCCAAGGGTTTCAGGTTGAGGAACGTAATGTGACAGTAGACGAATTACTAGAAGCGGACGAGGTTTTCTGCACAGGAACCGCTGTGGTTGTCTCTCCCGTTGGAAGCATTACTTACAAAGGCAAAAGAGTGTCATATGGAGAAGGTACCTTTGGAACTGTCTCGAAGCAACTCTACACTGTTCTGACAAGCTTGCAGATGGGTCTGAGTGAGGACAACATGAATTGGACTCTGAATCTGAGT

> CK_Unigene_BMK.21156 gi|297794191|ref|XP_002864980.1| 0 gi|297794191|ref|XP_002864980.1| ATBCAT-5 [Arabidopsis lyrata subsp. lyrata] AGAAGATCAACATCATCATCACAAAATTTCAAACTCTTTTACGAATTTGAGATTATGGAGCGAACCGGCGTTATCTCTAGTTTTCATCAAAGCTACATCCTCTCCCCTTCACGCGCCGCCACATCCACCAAGCCCCTCCACTCTTTCTCCTCCTCCCTCAGAAACTATCCATCTTCCTCTCTCAGGATTCGCCACTCTGCTTCTTCCGTCTCTTTCACTTCCCGTTACCGATGCGACGCCGTTTCACCCAATTCTTCCAGCACTGATGTGACTGAATTAGCCGAAATTGATTGGGACAACATCGACTTTGGGCTTAAACCAACGGACTACATGTACGCCATGAAATGTAGCCGTGATGGTGATTTCTCTAAAGGTCAATTGCAAAGTTTTGGGAACATTGAAGTCAGCCCTTCAGCTGCTGTACTCAACTATGGACAAGGTTTGTTTGAAGGTCTAAAAGCTTACAGAAAACATGACGGTAATATTCTCCTCTTCCGTCCCGAGGAGAATGCCATCCGAATGAGAAATGGTGCTGAAAGAATGTGCATGCCTGCTCCATCCGTCGAACAGTTTGTCGAGGCCGTGAAAACAACTGTATTAGCAAACAAACGCTGGATTCCACCTCCAGGTAAAGGATCATTGTACATAAGGCCATTGCTAATGGGAACTGGAGCTGTTCTTGGTCTTGCTCCTGCTCCCGACTACACTTTCCTTATCTATGTTTCACCCGTTGGTAACTACTTCAAGGAAGGTGTGGCGCCGATCAACTTGATTGTTGAAAATGATTTCCATCGTGCAACTCCTGGCGGTACTGGAGGTGTTAAAACCATCGGTAATTATGCTGCAGTCTTGAAGGCACAGTCGGCTGCGAAAGCTAAAGGGTACTCGGATGTTTTATATCTTGATTGCATTTACAAAAGATATATCGAGGAGGTTTCATCTTGCAACATTTTTATTGTGAAGGATAATGTGATCTCTACTCCTGAAATTAGAGGAACCATCTTGCCTGGAATTACTAGGAAGAGTATAATCGAAGTGGCTCGGAGCCAAGGGTTCAAGGTGGAGGAACGAAATGTGACAGTGGATGAATTGGTGGAAGCGGATGAAGTTTTCTGCACTGGAACCGCCGTTGTTTTATCACCGGTGGGAAGCATTACTTACAAAAGCCAAAGGGTTTCTTATGGAGAAGATGGATTTGGAACTGTGTCGAAACAACTATACACTTCCTTGACGAGCCTGCAAATGGGTCTGAGCGAAGATAACATGAACTGGACTGTTCAGTTGAGT

**CYP79F1**

>CL10668.Contig1_All 54 1664 CYP79F1 [Brassica oleracea]

ATGATGATGATGATGAGCGTTACCACATCATTGCCATACCCTTTTCAAATCCTACTAGTC

TTCATCATCTCCATGGCATCAATCACTTTACTAGGCCGAATGCTCTCAAAGCCCACTAAA

ACCAAAGACCGATCTCGTCAGCTTCCTCCAGGCCCACGAGGATGGCCCATCCTCGGCAAT

TTACCGGAACTAATCATGGCTCGTCCTAAATACAAATATTTTCACAATGCCATGAAACAG

CTAAACACGGAGATCGCATGTTTCAACTTCGCCGGAACTCACGCCATCACCATAAACTCC

GACGAGATAGCCAGAGAAGCTTTTAAAGAGCGAGATGCCGACTTCGCGAGCCGGCCTGAT

GTTTTCTCCATGAGGACCATCGGAGACAATTGGAAATCAATGGGGAACTCACCTTACGGT

GAACAGTTCGTGAAGATGAAAAGAGTGATCACAACGGAACTTATGTCCGCTAAAACGTTG

AACATGATGGTAGCTTCAAGAACCATCGAAGCGGATAATCTCATTGCTTACGTTCATTCG

ATGTATCAACGGTCCGAGACAGTGGACCTTAGGGAACTTTCAAGGGTTTACGGTTACGCA

ATGACCATGAGAATGATGTTTGGAAGGAGAAATATCACGAAAGATAACGTTTTGTCGGAA

GATGGGAGATTAACAAAAGCGGAAAGAGATCATCTTGAGGCGATTTTCGATACTCTAAAC

TGTCTGCCGAGTTTTAGTCCAGCGGATTACTTGGAAAGATGGTTCAAAGGTTGGAATATT

GATGGTCAAGAGGAGATGGTGAAAAAGAGCTGTAATTTTGTTCGTAGTTATAACAATCCG

ATCATCGACGAGAGGGTCAAATTATGGAGGGAGAAAGGTGGTAAGGCTGCTGTTGAAGAT

TGGATTGATACGTTCATTACACTCAAAGATGAAAACGGAAAGTACTTGATCACGCCAGAC

GAAATCAAAGCTCAATGCGTTGAATTTTGTATAGCAGCGATTGATAATCCGGCAAATAAC

ATGGAATGGACACTTGCTGAGATGTTGAAGAACCCGGAGGTTCTCAGGAAAGCTTTGAAG

GAGTTAGATGAAGTTGTGGGGAAAGAGAGGCTTGTTCAAGAATCAGACATACCAAATCTT

AACTACATAAAAGCTTGTTGCAGAGAAACATTCAGGATTCACCCAAGTGCTCATTTTATC

CCTCCCCATGTGGCTCGCCAAGATACCACCCTCGGGGGATATTTCATTCCCAAAGGTAGC

CACATTCATATATGCCGTTCTGAACTAGGACGGAGCCCTAAAATATGGAAAGATCCAATG

GTATACAAACCGGAGCGACACCTCGAAGATGGAATCTCGAAAGAGGTTACTCTGGTCGAA

ACAGAGCTGCGTTTCGTCTCGTTTGGCACCGGTCGACGTGGGTGCGTTGGTGTTAAAGTT

GGGACGATCATGATGGTTACATTGTTGGCTAGGTTTCTTCAAGCGTTTAACTGGAAACTC

GATCCTGGTTTTGGACCGTTAAGCCTCGAGGAAGATGATGTAATGCTTATGGCTAAGTCT

CTTCTCTTGTCCGTTGAGCCACGCTTGGCACCGAACCTTTATCCAAAATTT

> T_Unigene_BMK.15233 gi|171921116|gb|ACB59213.1| 0 gi|171921116|gb|ACB59213.1| CYP79F1 [Brassica oleracea]

TTTCACAACCCTCTCTACACACACACTCACACAAACATGATGATGATGATGAGCGTTACCACATCATTGCCATACCCTTTTCAAATCCTACTAGTCTTCATCATCTCTACAGCATCAATCACTTTACTAGGCCGAATGCTATCAAGGCCCACCAAAACCAAAGACCGATCTCGTCAGCTTCCTCCGGGCCCACGAGGATGGCCCATCCTCGGCAATTTACCGGAACTAATCATGGTTCGTCCTAAATACAAATATTTTCACAATGCCATGAAACAGCTAAACACGGAGATCGCATGTTTCAACTTCGCCGGAACTCACGCCATCACCATAAACTCCGACGAGATAGCCAGAGAAGCTTTTAAAGAGCGAGATGCCGACTTCGCGAGCCGGCCTGATGTTTTCTCCATGAGGACCATCGGAGACAATTGCAAATCAATGGGGAACTCACCTTACGGTGAACAGTTCGTGAAGATGAAAAGAGTGATCACAACGGAACTTATGTCCGCTAAAACGTTGAACATGATGGTAGCTTCAAGAACCATCGAAGCGGATAATCTCATTGCTTACGTTCACTCGATGTATCAACGGTCCGAGACAGTGGACCTTAGGGAACTTTCAAGGGTTTACGGTTACGCAATGACCATGAGAATGATGTTTGGAAGGAGAAATATCACGAAAGATAACGTTTTGTCGGAAGATGGGAGATTAACAAAAGCGGAAAGAGGTCATCTTGACGCGATTTTCGATACTCTAAACTGTTTGCCGAGTTTTAGTCCAGCGGATTACCTGGAAAGATGGTTCAAAGGTTGGAATATTGATGGTCAAGAGGAGATGGTGAAAAAGAGCTGTAATTTTGTTCGTAGTTACAACAATCCGATCATCGACGAGAGGGTCAAATTATGGAGGGAGAAAGGTGGTAAGGCTGCTGTTGAAGATTGGATTGATACGTTCATTACACTCAAAGATGAAAACGGAAAGTACTTGATCACGCCAGACGAAATCAAAGCTCAATGCGTTGAGTTTTGTATAGCGGCGATTGATAATCCGGCAAATAACATGGAATGGACACTTGCTGAGATGTTAAAGAACCCGGAGGTTCTCAGGAAAGCTTTGAAGGAGTTAGACGAAGTTGTGGGAAAAGAGAGGCTTGTTCAAGAATCAGACATACCAAACCTTAACTACATAAAAGCTTGTTGCAGAGAAACATTCAGGATTCACCCAAGTGCTCATTTTATCCCTCCCCATGTGGCTCGTCAAGATACCACCCTCGGAGGCTATTTCATTCCCAAAGGTAGCCACATTCATATATGCCGTTCTGAACTAGGACGGAGCCCTAAAATATGGAAAGATCCAATGGTATACAAACCGGAGCGACACCTCGAAGATGGAATCTCGAAAGAGGTTACTCTGGTCGAAACAGAGCTGCGTTTCGTCTCGTTTGGCACCGGTCGACGTGGGTGCGTTGGTGTTAAAGTCGGGACGATCATGATGGTTACATTGTTGGCTAGGTTTCTTCAAGCGTTTAACTGGAAACTCGATCCTGGTTTTGGACCGTTAAGCCTCGAGGAAGATGATGTAATGCTTATGGCTAAGCCTCTTCTCTTGTCCGTTGAGCCACGCTTGGCACCGAACCTTTATCCAAAATTTCGTCAT

**CYP79B2**

>CL1545.Contig1_All 64 1683 minus strand CYP79B2 [Arabidopsis lyrata subsp. lyrata] >gi|297312732|gb|EFH43155.1| CYP79B2 [Arabidopsis lyrata subsp. lyrata]

ATGAACAGTTTTACCTCAAACTCTTCGGATCTCACTTTAACTACAGCTGAAACATCGTTT

AGCAACCTGTATCTCCTCACAACAATTCAAGCCTTTGTGGCTATAACCTTACTGATGCTA

CTCAAGAAACTGATCACAGATCCCAGTAAAAAGAAACTGTCTCTCCCGCCGGGTCCCACC

GGATGGCCGATCGTCGGAATGGTTCCAGCGATGCTAAAGAGCCGCCCCGTTTTCCGGTGG

CTCCACAGCATCATGAAACAGCTCAACACTGAGATAGCTTGCGTGAGGCTAGGAAACACT

CACGTGATCACCGTCACGTGCCCTAAGATAGCACGTGAGATACTCAAGCAACAAGACGCT

CTCTTCGCCTCAAGACCTTTAACTTACGCACAGAAGATCCTCTCTAACGGCTACAAAACC

TGCGTGATCACTCCTTTTGGTGAACAATTCAAGAAAATGAGGAAAGTTGTGATGACGGAA

CTCATTTGCCCCGCGAGACACAGGTGGCTTCACCAGAAGAGAGCAGAAGAAAACGATCAT

TTAACCGCTTGGGTATACAACATGGTTAAGAACTCGGGCTCTGTCGATTTCCGGTTTGTC

ACTAGGCATTACTGTGGGAATGCAATCAAGAAACTTATGTTCGGGACAAGAACGTTCTCT

AAGAACACTGCACCTGACGGTGGACCGACCGTGGAAGATGTAGAGCATATGGAAGCTATG

TTTGAAGCATTAGGGTTTACGTTTGCTTTTTGTATTTCTGATTATCTACCGATGCTAACG

GGACTTGATCTTAACGGTCACGAGAAGATCATGAGAGAATCTAGTGCCATCATGGACAAG

TATCATGACCCAATCGTCGACGAGAGGATCAAGATGTGGAGAGAAGGAAAGAGAACTCAA

ATCGAAGATTTTCTTGATATATTCATCTCTATCAAGGACGAGGAAGGCAACCCATTGCTT

ACCGCCGATGAAATCAAACCCACCATTAAGGAGCTTGTAATGGCGGCGCCAGACAATCCA

TCAAACGCCGTCGAGTGGGCCATGGCGGAAATGGTAAACAAACCGGAGATTCTTCGGAAA

GCAATGGAAGAAATCGACAGAGTCGTCGGAAAAGAAAGACTTGTTCAAGAATCCGACATC

CCAAAACTAAACTACGTCAAAGCTATTCTCCGTGAAGCTTTCCGTCTTCATCCCGTCGCT

GCCTTTAACCTCCCACACGTGGCACTTTCCGACACAACCGTCGCCGGGTATCACATCCCT

AAAGGAAGTCAAGTCCTTCTTAGCCGATATGGGTTAGGCCGTAACCCAAAAGTTTGGGCT

GACCCACTTAGCTTTAAACCGGAGAGACATCTCAACGAGTGCTCCGAAGTTGCTTTGACT

GAGAACGATCTCCGGTTTATCTCGTTTAGTACCGGGAAAAGAGGTTGTGCTGCTCCAGCC

TTAGGAACGGCGTTGACCACGATGCTGCTCGCGAGACTTCTTCAAGGTTTCACTTGGAAA

CTACCGGAGAATGAGACACGTGTCGAGCTAATGGAGTCTAGTCATGATATGTTTTTGGCT

AAACCGTTGGTTATGGTCGGTGAGCCGAGATTACCGGAGCATCTTTACCCGACGGTGAAG

>CL1545.Contig2_All 61 1680 minus strand CYP79B2 [Arabidopsis lyrata subsp. lyrata] >gi|297312732|gb|EFH43155.1| CYP79B2 [Arabidopsis lyrata subsp. lyrata]

ATGAACAGTTTTACCTCAAACTCTTCGGATCTCACTTTAACTACAGCTGAAACATCGTTT

AGCAACCTGTATCTCCTCACAACAATTCAAGCCTTTGTGGCTATAACCTTACTGATGCTA

CTCAAGAAACTGATCACAGATCCCAGTAAAAAGAAACTGTCTCTCCCGCCGGGTCCCACC

GGATGGCCGATCGTCGGAATGGTTCCAGCGATGCTAAAGAGCCGCCCCGTTTTCCGGTGG

CTCCACAGCATCATGAAACAGCTCAACACTGAGATAGCTTGCGTGAGGCTAGGAAACACT

CACGTGATCACCGTCACGTGCCCTAAGATAGCACGTGAGATACTCAAGCAACAAGACGCT

CTCTTCGCCTCAAGACCTTTAACTTACGCACAGAAGATCCTCTCTAACGGCTACAAAACC

TGCGTGATCACACCGTTCGGCGAACAATTCAAGAAGATGAGGAAAGTGATAATGACGGAG

ATTGTTTGTCCGGCAAGACACAGATGGCTACACGACAATAGAGCTGAGGAAACCGATCAC

TTGACCGGTTGGCTTTACAACATGGTTAAAACCTCAGAACCGGTTGATCTCCGGTTTGTT

ACGAGGCATTACTGTGGAAATGCGATTAAGAGGCTTATGTTTGGAAAGAGAACGTTCTCG

GCGAAAACTGAAGCTGATGGTGGACCGACCGTTGAAGATATCGAGCATATGGATGCTATG

TTCGATGGTTTAGGGTTTACATTTGCGTTTTGTGTATCGGATTATCTACCGATACTTACG

GGATTGGATTTGAACGGACATGAGAAGATCATGAGAGAAGCTAGTGCGATTATGGATAAA

TATCACGATCCTATTATTAATGAGAGGATTAAGATGTGGAAAGATGGTAAGAGAACTCAG

ATCGAAGATTTTCTAGACATTTTCATTTCTATCAAGGGTGAAGATGGCCAGCCTTTGCTT

ACCGCTGATGAAATCAAACCAACCATTAAGGAACTTGTAATGGCGGCGCCGGACAACCCA

TCAAACGCCGTGGAATGGGCCATGGCAGAGATGATAAACAAACCGGAGATCCTCCACAAA

GCTATGGAAGAGATCGATAGAGTCGTAGGCAAAGAAAGATTGGTCCAAGAATCTGACATC

CCAAAACTTAACTACGTCAAAGCTATTCTCCGTGAAGCTTTCCGTCTTCATCCCGTCGCT

GCCTTTAACCTCCCACACGTGGCACTTTCCGACACAACCGTCGCCGGGTATCACATCCCT

AAAGGAAGTCAAGTCCTTCTTAGCCGATATGGGTTAGGCCGTAACCCAAAAGTTTGGGCT

GACCCACTTAGCTTTAAACCGGAGAGACATCTCAACGAGTGCTCCGAAGTTGCTTTGACT

GAGAACGATCTCCGGTTTATCTCGTTTAGTACCGGGAAAAGAGGTTGTGCTGCTCCAGCC

TTAGGAACGGCGTTGACCACGATGCTGCTCGCGAGACTTCTTCAAGGTTTCACTTGGAAA

CTACCGGAGAATGAGACACGTGTCGAGCTAATGGAGTCTAGTCATGATATGTTTTTGGCT

AAACCGTTGGTTATGGTCGGTGAGCCGAGATTACCGGAGCATCTTTACCCGACGGTGAAG

>T_Unigene_BMK.13225 gi|297798024|ref|XP_002866896.1| 0 gi|297798024|ref|XP_002866896.1| CYP79B2 [Arabidopsis lyrata subsp. lyrata]

ACAATATTTGAGTCTTCTTCTTCTCTCTGTCTCTCTTCTTTACAAACAAACATGAACACTTTTACCTCAAACTCTTCGGATCTCACTTCAACTACAGCTGAAACATCGTTTAGCAACCTGTATCTCCTCACAACAATTCAAGCCTTTGTGGCTATAACCTTAGTGATGATACTCAAGAAACTGATCACAGATCCCAGTAAAAAGAAACTGTCTCTCCCGCCGGGTCCCACCGGATGGCCGATCGTCGGAATGGTTCCAGCGATGCTAAAGAGCCGCCCCGTTTTCCGGTGGCTCCACAGCATCATGAAACAGCTCAACACTGAGATAGCTTGCGTGAGGCTAGGAAACACTCACGTGATCACCGTCACGTGCCCTAAGATAGCACGTGAGATACTCAAGCAACAAGACGCTCTCTTCGCCTCAAGACCTTTAACTTACGCACAGAAGATCCTCTCTAACGGCTACAAAACCTGCGTGATCACTCCTTTTGGTGAACAATTCAAGAAAATGAGGAGAGTTGTGATGACGGAACTCGTTTGCCCCGCGAGACACAGGTGGCTTCACCAGAAGAGATCAGAAGAAAACGATCATTTAACCGCTTGGGTATACAACATGGTTAAGAACTCGGGCTCTGTCGATTTCCGGTTTGTCACTAGGCATTACTGTGGGAATGCAATCAAGAAACTTATGTTCGGGACAAGAACGTTCTCTAAGAACACTGCACCTGACGGTGGACCGACCGTGGAAGATGTAGAGCATATGGAAGCCATGTTTGAAGCATTAGGGTTTACGTTTGCTTTTTGTATTTCTGATTATCTACCGTTGCTAACGGGACTTGATCTTAACGGTCACGAGAAGATCATGAGAGAATCTAGTGCCATTATGGACAAGTATCATGACCCAATCGTCGACGAGAGGATCAAGATGTGGAGAGAAGGAAAGAGAACTCAGATCGAAGATTTTCTTGATATTTTCATCTCTATCAAGGACGACGAAGGCAACCCATTGCTTACCGCCGATGAAATCAAACCCACCATTAAGGAGCTTGTAATGGCGGCGCCAGACAATCCATCAAACGCCGTCGAGTGGGCCATGGCGGAAATGGTAAACAAACCGGAGATTCTTCGGAAAGCAATGGAAGAAATCGACAGAGTCGTCGGAAAAGAAAGACTTGTTCAAGAATCCGACATCCCAAAACTAAACTACGTCAAAGCTATTCTCCGTGAAGCTTTCCGTCTTCATCCCGTCGCTGCCTTTAACCTACCACACGTGGCACTTTCCGACACAACCGTCGCCGGATATCACATCCCTAAAGGAAGTCAAGTCCTTCTTAGCCGATATGGGCTAGGCCGTAACCCAAAAGTTTGGGCTGACCCACTTAGCTTTAAACCGGAGAGACATCTCAACGAGTGCTCCGAAGTTACTTTGACTGAGAACGATCTCCGGTTTATCTCGTTTAGCACCGGGAAAAGAGGTTGTGCTGCTCCAGCCTTAGGAACGGCGTTGACCACGATGCTGCTCGCGAGACTTCTTCAAGGTTTCACTTGGAAACTACCGGAGAATGAGACACGTGTCGAGCTAATGGAGTCTAGTCATGATATGTTTTTGGCTAAACCGTTGGTTATGGTCGGTGAGCCGAGATTACCAGAGCATCTTTACCCGACGGTGAAG

**CYP83A1**

>CL13253.Contig1_All 55 1446 CYP83B1 [Arabidopsis lyrata subsp. lyrata] >gi|297313135|gb|EFH43558.1| CYP83B1 [Arabidopsis lyrata subsp. lyrata]

ATGGATCTCTTCTTGATTATTGCCGCCCTGGCCGCCCTGGTAGCCTTCTTCTTCCTCCGG

AGCACCACCAAAAAAACTCTCCGGCTACCTCCGGGGCCGAAAGGTCTTCCTATAATCGGA

AACCTCCACCAGATGGAAAAATCCAACCCACAACACTTCCTTTTCCGTCTTTCCAAGCTA

TACGGCCCAATTTTCACAATGAAAATCGGAGGCCTCCGCCTAGCGGTGATCTCCTCAGCT

GAGCTAGCTAAAGAGCTTCTCAAGACCCAAGACCTCAATTTCACCGCTCGTCCTCCTCTA

AAAGGGCAACAAACGATGTCGTATCAAGGCCGTGAGCTTGGTTTCGGACAGTACACAGCT

TACTACCGTGAGATGAGGAAGATGTGTATGGTCAATCTCTTCAGTCCAAACCGTGTGGCA

AGTTTCCGACCCGTTAGAGAAGAAGAGTGCAGTCGGATGATGGACAAGATCTACAAAGCC

GCTGATCAATCAGGCACCGTTGATCTTAGTGAGCTTCTCTTGTCCTTCACCAACTGTGTC

GTCTGCAGACAAGCATTTGGGAAGCGCTATAATGAGTACGGGACCGAGATGAAAAGATTC

ATAAACATCTTGTACGAGACTCAAGCCCTTTTGGGTACTCTGTTTTTCTCCGACCTTTTC

CCTTATTTTGGATTCCTTGACAACATCACTGGTCTCAGTGCGCGTATCAAGAAAGCTTTC

AAGGAGCTCGACACTTACCTTCAAGAACTCCTCGACGAGACTCTTGAACCTAATCGCCCT

AAACCCGAGACAGAGAGTTTCATTGATCTTTTGATGCAGATCTACAAAGATCAACCTTTT

TCCGTCAAATTCACTCACGAAAATGTCAAGGCCATGATATTGGATATTGTTGTGCCGGGA

ACTGACACAGCGGCTGCGGTGGTGGTATGGGCCATGACTTACCTTATAAAGTACCCAGAA

GCAATGAAGAAAGCTCAAGAAGAAGTGAGGAATGTCGTAGGCGACAAAGGATATGTCTCA

GAAGAAGACATTCCTAATCTCCCTTACCTGAAGGCCGTTATAAAGGAGTCTCTCCGGCTC

GAACCTGTTATCCCCATTCTTCTACCCAGAGAAACCATCGCAGACGCAAAGATTGGTGGC

TATGATATCCCGGCCAAGACCATCATTCAGGTGAACGCGTGGGCGGTTTCTCGTGACACA

GCTGCGTGGGGAGACAACCCTAATGAGTTCATTCCAGAGAGGTTCATGAACAAGTACAAA

GGAGTGGACTTCAAGGGGCAAGATTTTGAGCTCCTACCTTTCGGGTCGGGCCGGAGAATG

TGCCCCGCAATGCATCTTGGGGTTGCAATGGTAGAGATACCTTTCGCTAACCTTCTCTAC

AGATTTGACTGG

> CK_Unigene_BMK.12101 gi|197090681|gb|ACH41741.1| 0 gi|197090681|gb|ACH41741.1| CYP83B1 [Brassica rapa subsp. pekinensis]

CCGCAGTGGCCGCCCTGGCAGCCTTCTTCTTCCTCCGGAGCACCACCAAAAAAACCTCTCCGGCTACCTCCGGGGCCGAAAGGTCTTCCTATAATCGGAAACATCCACCAGATGGAAAAATCCAACCCACAACACTTCCTTTTCGGTCTTTCCAAGCTATACGGCCCAATTTTCACAATGAAAATGGGAGGCCGCCGCCTAGCGGTGATCTCCTCAGCTGAGCTAGCTAAAGAGCTTCTCAAGACCCAAGACCTCAATTTCACCGCTCGTCCTCTTCTAAAAGGGCAACAAACGATGTCGTATCAAGGCCGTGAGCTTGGTTTCGGACAGTACACAGCTTACTACCGTGAGATGAGGAAGATGTGTATGGTCAATCTCTTCAGTCCAAACCGTGTGGCAAGTTTCCGACCCGTTAGAGAAGAAGAGTGCAGTCGGATGATGGACAAGATCTACAAAGCCGCTGATGAATCAGGCACCGTTGATATTAGTGAGCTTCTCTTGTCCTTCACCAACTGTGTCGTCTGCAGACAAGCATTTGGGAAGCGCTATAATGAGTACGGGACCGAGATGAAAAGATTCATAAACATCTTGTACGAGACTCAAGCCCTTTTGGGTACTCTGTTTTTCTCCGACCTTTTCCCTTATTTTGGATTCCTTGACAACATCACTGGTCTCAGTGCGCGTATCAAGAAAGCTTTCAAGGAGCTCGACACTTACCTTCAAGAACTCCTCGACGAGACTCTTGAACCTAATCGCCCTAAACCCGAGACAGAGAGTTTCATTGATCTTTTGATGCAGATCTACAAAGATCAACCTTTTTCCGTCAAATTCACTCACGAAAATGTCAAGGCCATGATATTGGATATTGTTGTGCCGGGAACTGACACAGCGGCTGCGGTGGTGGTATGGGCCATGACTTACCTTATAAAGTACCCAGAAGCAATGAAGAAAGCTCAAGAAGAAGTGAGGAATGTCGTAGGCGACAAAGGATATGTGTCAGAAGAAGACATTCCTAATCTCCCTTACCTGAAGGCCGTTATAAAGGAGTCTCTCCGGCTCGAACCTGTTATCCCCATTCTTCTACCCAGAGAAACCATCGCAGACGCAAAGATTGGTGGCTATGATATCCCGGCCAAGACCATCATTCAGGTGAACGCGTGGGCGGTTTCTCGTGACACAGCTGCGTGGGGAGACAACCCTAATGAGTTCATTCCAGAGAGGTTCATGAACGAGTACAAAGGAGTGGACTTCAAGGGGCAAGATTTTGAGCTCCTACCATTCGGGTCGGGCCGGAGAATGTGCCCCGCAATGCATCTTGGGGTTGCAATGGTAGAGATACCTTTCGCTAACCTTCTCTACAGATTTGACTGGAGTCTACCTAAAGGGATTAAACCAGAAGATATAAAGATGGACGTCATGACCGGACTCGCAATGCACAAGAAAGAGCACCTTGTCCTTGCACCAAGGACCCACATT

**CYP83B1**

>CL13253.Contig1_All 55 1446 CYP83B1 [Arabidopsis lyrata subsp. lyrata] >gi|297313135|gb|EFH43558.1| CYP83B1 [Arabidopsis lyrata subsp. lyrata]

ATGGATCTCTTCTTGATTATTGCCGCCCTGGCCGCCCTGGTAGCCTTCTTCTTCCTCCGG

AGCACCACCAAAAAAACTCTCCGGCTACCTCCGGGGCCGAAAGGTCTTCCTATAATCGGA

AACCTCCACCAGATGGAAAAATCCAACCCACAACACTTCCTTTTCCGTCTTTCCAAGCTA

TACGGCCCAATTTTCACAATGAAAATCGGAGGCCTCCGCCTAGCGGTGATCTCCTCAGCT

GAGCTAGCTAAAGAGCTTCTCAAGACCCAAGACCTCAATTTCACCGCTCGTCCTCCTCTA

AAAGGGCAACAAACGATGTCGTATCAAGGCCGTGAGCTTGGTTTCGGACAGTACACAGCT

TACTACCGTGAGATGAGGAAGATGTGTATGGTCAATCTCTTCAGTCCAAACCGTGTGGCA

AGTTTCCGACCCGTTAGAGAAGAAGAGTGCAGTCGGATGATGGACAAGATCTACAAAGCC

GCTGATCAATCAGGCACCGTTGATCTTAGTGAGCTTCTCTTGTCCTTCACCAACTGTGTC

GTCTGCAGACAAGCATTTGGGAAGCGCTATAATGAGTACGGGACCGAGATGAAAAGATTC

ATAAACATCTTGTACGAGACTCAAGCCCTTTTGGGTACTCTGTTTTTCTCCGACCTTTTC

CCTTATTTTGGATTCCTTGACAACATCACTGGTCTCAGTGCGCGTATCAAGAAAGCTTTC

AAGGAGCTCGACACTTACCTTCAAGAACTCCTCGACGAGACTCTTGAACCTAATCGCCCT

AAACCCGAGACAGAGAGTTTCATTGATCTTTTGATGCAGATCTACAAAGATCAACCTTTT

TCCGTCAAATTCACTCACGAAAATGTCAAGGCCATGATATTGGATATTGTTGTGCCGGGA

ACTGACACAGCGGCTGCGGTGGTGGTATGGGCCATGACTTACCTTATAAAGTACCCAGAA

GCAATGAAGAAAGCTCAAGAAGAAGTGAGGAATGTCGTAGGCGACAAAGGATATGTCTCA

GAAGAAGACATTCCTAATCTCCCTTACCTGAAGGCCGTTATAAAGGAGTCTCTCCGGCTC

GAACCTGTTATCCCCATTCTTCTACCCAGAGAAACCATCGCAGACGCAAAGATTGGTGGC

TATGATATCCCGGCCAAGACCATCATTCAGGTGAACGCGTGGGCGGTTTCTCGTGACACA

GCTGCGTGGGGAGACAACCCTAATGAGTTCATTCCAGAGAGGTTCATGAACAAGTACAAA

GGAGTGGACTTCAAGGGGCAAGATTTTGAGCTCCTACCTTTCGGGTCGGGCCGGAGAATG

TGCCCCGCAATGCATCTTGGGGTTGCAATGGTAGAGATACCTTTCGCTAACCTTCTCTAC

AGATTTGACTGG

> CK_Unigene_BMK.12101 gi|197090681|gb|ACH41741.1| 0 gi|197090681|gb|ACH41741.1| CYP83B1 [Brassica rapa subsp. pekinensis]

CCGCAGTGGCCGCCCTGGCAGCCTTCTTCTTCCTCCGGAGCACCACCAAAAAAACCTCTCCGGCTACCTCCGGGGCCGAAAGGTCTTCCTATAATCGGAAACATCCACCAGATGGAAAAATCCAACCCACAACACTTCCTTTTCGGTCTTTCCAAGCTATACGGCCCAATTTTCACAATGAAAATGGGAGGCCGCCGCCTAGCGGTGATCTCCTCAGCTGAGCTAGCTAAAGAGCTTCTCAAGACCCAAGACCTCAATTTCACCGCTCGTCCTCTTCTAAAAGGGCAACAAACGATGTCGTATCAAGGCCGTGAGCTTGGTTTCGGACAGTACACAGCTTACTACCGTGAGATGAGGAAGATGTGTATGGTCAATCTCTTCAGTCCAAACCGTGTGGCAAGTTTCCGACCCGTTAGAGAAGAAGAGTGCAGTCGGATGATGGACAAGATCTACAAAGCCGCTGATGAATCAGGCACCGTTGATATTAGTGAGCTTCTCTTGTCCTTCACCAACTGTGTCGTCTGCAGACAAGCATTTGGGAAGCGCTATAATGAGTACGGGACCGAGATGAAAAGATTCATAAACATCTTGTACGAGACTCAAGCCCTTTTGGGTACTCTGTTTTTCTCCGACCTTTTCCCTTATTTTGGATTCCTTGACAACATCACTGGTCTCAGTGCGCGTATCAAGAAAGCTTTCAAGGAGCTCGACACTTACCTTCAAGAACTCCTCGACGAGACTCTTGAACCTAATCGCCCTAAACCCGAGACAGAGAGTTTCATTGATCTTTTGATGCAGATCTACAAAGATCAACCTTTTTCCGTCAAATTCACTCACGAAAATGTCAAGGCCATGATATTGGATATTGTTGTGCCGGGAACTGACACAGCGGCTGCGGTGGTGGTATGGGCCATGACTTACCTTATAAAGTACCCAGAAGCAATGAAGAAAGCTCAAGAAGAAGTGAGGAATGTCGTAGGCGACAAAGGATATGTGTCAGAAGAAGACATTCCTAATCTCCCTTACCTGAAGGCCGTTATAAAGGAGTCTCTCCGGCTCGAACCTGTTATCCCCATTCTTCTACCCAGAGAAACCATCGCAGACGCAAAGATTGGTGGCTATGATATCCCGGCCAAGACCATCATTCAGGTGAACGCGTGGGCGGTTTCTCGTGACACAGCTGCGTGGGGAGACAACCCTAATGAGTTCATTCCAGAGAGGTTCATGAACGAGTACAAAGGAGTGGACTTCAAGGGGCAAGATTTTGAGCTCCTACCATTCGGGTCGGGCCGGAGAATGTGCCCCGCAATGCATCTTGGGGTTGCAATGGTAGAGATACCTTTCGCTAACCTTCTCTACAGATTTGACTGGAGTCTACCTAAAGGGATTAAACCAGAAGATATAAAGATGGACGTCATGACCGGACTCGCAATGCACAAGAAAGAGCACCTTGTCCTTGCACCAAGGACCCACATT

**GSTF9**

>CL11782.Contig1_All 142 780 glutathione S-transferase [Eutrema halophilum]

ATGGTGCTAAAGGTGTACGGACCTCACTTTGCTTCACCGAAGAGAGCTCTGGTAACACTG

ATTGAGAAGGGCGTCGCCTTCGAGACTGTCCCCGTCGATCTCATGAAAGGAGAACACAAG

CAGCCTGCTTTTCTCGCTTTACAGCCTTTCGGTACTGTTCCTGCTGTTGTCGACGGCGAC

TACAAAATCTTCGAGTCACGAGCAGTGATGAGGTACATAGCTGAGAAGTACAGATCACAA

GGACCTGACCTTTTAGGGAAAACAGTTGAAGACAGAGGTCAAGTTGAACAATGGCTTGAC

GTTGAAGCAACCACTTACCACCCACCGCTACTTAACCTAACCCTACACATAATGTTTGCA

TCAGTCATGGGATTCCCATCAGATGAGAAACTGATCAAGGAAAGCGAAGAGAAACTCTCG

GCTGTTCTTGATGTGTACGAGGCACATCTGTCAAAGAGCAAGTACTTGGCTGGTGATTTC

GTTAGCTTGGCTGATTTGGCTCACCTTCCTTTCACTGATTACTTGGTTGGTCCGATTGGG

AAAGCTTACATGATCAAAGATAGGAAGCATGTGAGTGCTTGGTGGGATAATATTAGTAGC

CGTCCTGCTTGGAAGGAGACTCTTGAGAAGTATTCACTA

>CK_Unigene_BMK.21730 gi|297837145|ref|XP_002886454.1| 2.35347e-146 gi|297837145|ref|XP_002886454.1| hypothetical protein ARALYDRAFT_475075 [Arabidopsis lyrata subsp. lyrata] ATGCATTGCCCATTCAAAAAAAAAAAAAAAAAAAAAAATCTAATGGCTACGCTCTCAATCGGAATCGCCACCGCAACCACCGTCCGTACAATTCCGAAATTTAGTACACGGAGGAGCAAAATCTCCTGTGAATGGGATCCGAAAGGTTTGCTAGGTCCAGCTCAAACTGGTCATATCGCTCGTCTTGAGTTTAAGCGTAAGATAGAGAGAGATTCAGAAGCAAGAGAAGCTTTTCAGAAGCAACTTCGTGAGGAGAAAGAGCGTCGTCAAGCTCTTAGACAAGCTAGAGTTGTGCCAGATACTTCGGCTGAGCTGATTGAGTACTTTCTTGATACTGAAGCTCAGGAGATTGAGTATGAGATTGCTAGGCTTAGAGGAAGGTTAAACGATGAATTCTTTGCGCAGATTCGACTTGAAATCGGGCAAATTCGGTTTGCTGTAACAAAGACTGCGGAAAATGAAGACAGATTGATTGAGCTTGAATCACTTCAAAAAGCCTTAGAAGAAGGAATAGAGGCTTATGACAAAATGCAAAAGGAGCTTATGACAGCTACAAATAGCTTAACCAAGATCTTAACCTCAACCGATATTAAAGCGACATTGTTGGATATGGTTGAGAAAAACGAAATCAACAGATCTTTGTTAACACTTCTTGACGAAAACATAGCTAATGCATACAGAGGAAACCAGAAAGAAGCAGGAGATTACATGGAGAAGGTACGTTCTTCGGTTCTAAAGTACTTGACGGTG

**GSTF10**

>CL10342.Contig1_All 121 765 minus strand early dehydration-induced 13 [Arabidopsis lyrata subsp. lyrata] >gi|297325122|gb|EFH55542.1| early dehydration-induced 13 [Arabidopsis lyrata subsp. lyrata]

ATGGTGTTGACGATCTACGCGCCTTTGTACGCTTCTTCAAAGCGAGCTGTGGTAACGTTG

GTAGAGAAGGGAGTAGAGTTCGAGACCGTCAATGTCGATCTCCTGAAAGGAGAACAGAAG

CAGCCTGAGTATATCGCGATTCAGCCTTTCGGTAAAATCCCAGTGCTCGTCGATGGAGAC

TACAAAATCTTCGAGTCGCGTGCGATCATGAGGTACATAGCAGAGAAGTACAGATCACAA

GGACCAGATCTTTTGGGGAAGACGATTGAAGAAAGAGGACAAGTAGAGCAATGGCTAGAC

GTGGAAGCAACAAGCTACCACCCACCACTATTGGCTTTAACACTTAACATTATCTTTGCA

CCACTCATGGGATTACCAGCTGATGAGAAAGTTATTAAAGAGAGTGAAGAGAAGCTTGGA

GAAGTGCTTGATGTCTATGAAGCACAGCTCTCCAAGAACGAATACTTGGCTGGTGATTTT

GTCAGTCTAGCTGATTTAGCTCACCTTCCCTTCACTGAGTATCTCTTTGGTCCTATTGGG

AAACCTTATTTGATCAAAGATAGGAAGCATGTTAGCGCCTGGTGGGATAAGATTAGTAAC

CGTCCTGCGTGGAAGGAGGTTTCTGAGAAGTTCGCATTGACCGTT

> CK_Unigene_BMK.21162 gi|312283281|dbj|BAJ34506.1| 1.14861e-140 gi|312283281|dbj|BAJ34506.1| unnamed protein product [Thellungiella halophila]

CTCCCATTTCTAACCAAGAACAACTCTTCATCATCATATTCTTCAAACTCGAATTCAAATTCATCATCCTCTTCATGGCCATGGCCTTCTTGTCATCAAAATCCTAAAACCATATCTTTCAGAGCCACCATCACTTTCACAAACCCTATCCACGACCAAGACGACGATGAGCTCGATCCACCGGAGATTACAGACTCGATAGAGAATGTGATAAAAGGGCTAAGATCATCAAAGAGACTCATCTTCGAAAGCAAAGGAGAATCCAACTCTATACTTGAACAAGCTACGACTAAGCGAGAAGAAGAGAAAGAAGAAGACGAAGAAGAAGAAGGATCAGAGGAAGAAGAAAAAATGGTGTTGACGATCTACGCGCCTTTGTACGCTTCTTCAAAGCGAGCTGTGGTAACGTTGGTAGAGAAGGGAGTAGAGTTCGAGACTGTCAATGTCGATCTCCTCAAAGGAGAACAGAAGCAGCCTGAGTATATCGCGATTCAGCCTTTCGGTAAAATCCCAGTGCTCGTCGATGGAGACTACAAAATCTTCGAGTCGCGTGCGATCATGAGGTACATAGCAGAGAAGTACAGATCACAAGGACCAGATCTTTTGGGGAAGACGATTGAAGAAAGAGGACAAGTAGAGCAATGGCTAGATGTGGAAGCAACAAGCTACCACCCACCACTATTGGCTTTAACACTTAACATTATCTTTGCACCACTCATGGGATTACCAGCTGATGAGAAAGTTATTAAAGAGAGTGAAGAGAAGCTTGGAGAAGTGCTTGATGTCTATGAAGCACAACTCTCCAAGAACGAATACTTGGCTGGTGATTTTGTCAGTCTAGCTGATTTAGCTCACCTTCCTTTCACTGAGTATCTCTTTGGTCCTATTGGGAAACCTTATTTGATCAAAGATAGGAAGCATGTTAGCGCCTGGTGGGATAAGATTAGTAACCGTCCTGCGTGGAAGGAGGTTTCTGAGAAGTTCGCATTGACCCTT

**GSTF11**

>CL7458.Contig2_All 69 710 minus strand glutathione S-transferase F11 [Arabidopsis lyrata subsp. lyrata] >gi|297328119|gb|EFH58538.1| glutathione S-transferase F11 [Arabidopsis lyrata subsp. lyrata]

ATGGTAGTCAAGGTATATGGGCAGATCAAAGCAGCTAATCCTCAAAGAGTATTGCTCTGT

TTTTTGGAAAAAGGCATTGAGTTTGAAGTAATTCATGTAGATCTCGATAAACAAGAGCAG

AACAAACCAGAACATCTTCTTCGTCAGCCGTTTGGTAAAGTTCCAGCTATTGAAGATGGA

GATCTGAAGCTTTTTGAGTCGCGAGCCATAGCGAGGTACTATGCAACCAAGTATGCGGAC

CAAGGAACGGATCTATTGGGCAAGACTTTGGAGAAACGAGCCATCGTGGACCAGTGGATG

GAAGTTGAAGCTAACTATTTCTACGTTGTGGCTCTACCCTTAGTTGTTAACATCGTCTTT

AAGCCCAAGTTTGGTGAGCCATGCGACGTCGCTTTGGTCGAGGAGCTAAAGGTCAAGTTA

GAGAATATCCTGGATGTGTATGAGAACCGGTTAGCTACGAACCTGTACTTAGCCGGTGAT

GAATTCACATTGGCTGATTTGACTCATATGCCCGGTATGAGATATATGATGAATGAAACC

AGTTTGAGCGGTATGATTACATCTCGAAAGAATGTTAACCGGTGGTGGAATGAGATTTCG

GCTAGACCGGCTTGGAATAAGCTCATGGAATTGGCTGCCTAT

>T_Unigene_BMK.25038 gi|297828794|ref|XP_002882279.1| 1.67587e-140 gi|297828794|ref|XP_002882279.1| glutathione S-transferase F11 [Arabidopsis lyrata subsp. lyrata]

TTACATATAAACTTATTTAATCAGAGAATGGTAGTCAAGGTATATGGGCAGATAAAAGCAGCTAATCCTCAAAGAGTATTGCTCTGTTTTTTGGAAAAAGGCATTGAGTTTGAAGTAATTCATGTAGATCTCGATAAACAAGAGCAGAACAAACCAGAACATCTTCTTCGTCAGCCGTTTGGTAAAGTTCCAGCTATTGAAGATGGAGATCTGAAGCTTTTTGAGTCGCGAGCCATAGCGAGGTACTATGCAACCAAGTATGCGGACCAAGGAACGGATCTATTGGGCAAGACTTTGGAGAAACGAGCCATCGTGGACCAGTGGATGGAAGTTGAAGCTAACTATTTCTACGTTGTGGCTCTACCCTTAGTTGTTAACATCGTCTTTAAGCCCAAGTTTGGTGAGCCATGCGACGTCGCTTTGGTCGAGGAGCTAAAGGTCAAGTTAGAGAATATCCTGGATGTGTATGAGAACCGGTTAGCTACGAACCCGTACTTAGCCGGTGATGAATTCACATTGGCTGATTTGACTCATATGCCCGGTATGAGATATATGATGAATGAAACCAGTTTGAGCGGTATGATTACATCTCGAAAGAATGTTAACCGGTGGTGGAATGAGATTTCGGCTAGACCGGCTTGGAAGAAGCTCATGGAATTGGCTGCCTAT

**GSTU20**

>CL1055.Contig4_All 111 758 minus strand glutathione S-transferase TAU 19 [Arabidopsis thaliana] >gi|75338923|sp|Q9ZRW8.1|GSTUJ_ARATH RecName: Full=Glutathione S-transferase U19; Short=AtGSTU19; AltName: Full=GST class-tau member 19; AltName: Full=Glutathione S-transferase 8 >gi|14326477|gb|AAK60284.1|AF385691_1 At1g78380/F3F9_11 [Arabidopsis thaliana] >gi|4006934|emb|CAA10060.1| glutathione transferase [Arabidopsis thaliana] >gi|18700206|gb|AAL77713.1| At1g78380/F3F9_11 [Arabidopsis thaliana] >gi|332197978|gb|AEE36099.1| glutathione S-transferase TAU 19 [Arabidopsis thaliana] ATGGCGAACGAAGTGATCCTTCTTGATTTCTGGCCGAGTATGTTCGGGATGAGGACGAGG

ATCGCGTTGAGGGAGAAAGGCGTGGAGTTCGAGTACAGAGAAGAGGATCTGAGAAACAAG

AGCCCTTTGCTTCTCCAGATGAATCCGGTTCACAAGAAGATTCCGGTTCTTATCCACAAC

GGTAAACCGGTTAACGAATCTATCATCCAGGTTCAGTATATCGATGAGGTCTGGTCTCAC

AAGAACCCTATCCTTCCTTCTGATCCTTACCAGAAGGCTCAAGCTAGATTCTGGGCTGAT

TTCATTGACAAAAAGATGTACGAAGCCCAGAGGAAGGTTTGGGCGACTAAGGGTGAGGAA

CAAGAGGCAGGCAAGAAGGAGTTCATTGAGATACTCAAGACTCTTGAAAATGAGCTTGGA

GATAAGCCTTACTTTGGTGGCGATGACTTTGGTTATGTAGACATTGCATTCATTGGTTTC

TACACCTGGTTTCCAGCATACGAGAAGTTTGGTAACTTCAGCATCGAATCTGAGTGTCCG

AAACTAATCGCTTGGGCTAAGAAGTGTTTGCAGAGGGAGAGTGTGGCTAAGTCCTTGCCT

GATCCCGAGAAGGTTACTGGATTCGTGTCTGATCTCAGGAAGAAATTT

>CL1055.Contig1_All 114 761 glutathione S-transferase TAU 19 [Arabidopsis thaliana] >gi|75338923|sp|Q9ZRW8.1|GSTUJ_ARATH RecName: Full=Glutathione S-transferase U19; Short=AtGSTU19; AltName: Full=GST class-tau member 19; AltName: Full=Glutathione S-transferase 8 >gi|14326477|gb|AAK60284.1|AF385691_1 At1g78380/F3F9_11 [Arabidopsis thaliana] >gi|4006934|emb|CAA10060.1| glutathione transferase [Arabidopsis thaliana] >gi|18700206|gb|AAL77713.1| At1g78380/F3F9_11 [Arabidopsis thaliana] >gi|332197978|gb|AEE36099.1| glutathione S-transferase TAU 19 [Arabidopsis thaliana]

ATGGCGGACGAAGTGATTCTTCTAGATTTCTGGCCGAGCATGTTCGGGATGAGGACGAAG

ATGGCTTTGGCAGAGAAAGGAGTGGTGTATGAGTACAGGGAAACAAATCCATGGGTTAAG

ACTCCTTTGCTCATCGAGATGAACCCTATTCACAAGAAGATTCCGATTCTTATCCACAAC

GGTAAACCCATCTGTGAATCTCTAATTCAGCTTGAGTACATCGATGAGGTTTGGTCTCAT

ACAAACCCTATTCTTCCTTCTGATCCTTACCACAAAGCTCAAGCTAGATTCTGGGCTGAT

TTCATTGACAAAAAGATGTACGAAGCCCAGAGGAAGGTTTGGGCGACTAAGGGTGAGGAA

CAAGAGGCAGGCAAGAAGGAGTTCATTGAGATACTCAAGACTCTTGAAAATGAGCTTGGA

GATAAGCCTTACTTTGGTGGCGATGACTTTGGTTATGTAGACATTGCATTCATTGGTTTC

TACACCTGGTTTCCAGCATACGAGAAGTTTGGTAACTTCAGCATCGAATCTGAGTGTCCG

AAACTAATCGCTTGGGCTAAGAAGTGTTTGCAGAGGGAGAGTGTGGCTAAGTCCTTGCCT

GATCCCGAGAAGGTTACTGGATTCGTGTCTGATCTCAGGAAGAAATTT

> CK_Unigene_BMK.26299 gi|18411929|ref|NP_565178.1| 1.9483e-148 gi|18411929|ref|NP_565178.1| ATGSTU19 (GLUTATHIONE S-TRANSFERASE TAU 19); glutathione binding / glutathione transferase [Arabidopsis thaliana]

GGTATCGAAAGAATGGCGAACGAAGTGATCCTTCTTGATTTCTGGCCGAGTATGTTCGGGATGAGGACGAGGATCGCGTTGAGGGAGAAAGGCGTGGAGTTCGAGTACAGAGAAGAGGATCTGAGAAACAAGAGCCCTTTGCTTCTCCAGATGAATCCGATTCACAAGAAGATTCCGGTTCTTATCCACAACGGTAAACCGGTTAACGAATCTATCATCCAGGTTCAGTACATCGATGAGGTCTGGTCTCACAAGAACCCTATCCTTCCTTCTGATCCTTACCAGAAGGCTCAAGCTAGATTCTGGGCTGATTTCATTGACAAAAAGATGTACGAGGCCCAGAGGAAGGTTTGGGCGACCAAGGGTGAGGAACAAGAGGCAGGCAAGAAGGAGTTCATTGAGATACTCAAGACTCTTGAAAATGAGCTTGGAGATAAGCCTTACTTTGGTGGCGATGACTTTGGTTATGTAGACATTGCATTCATTGGTTTCTACACCTGGTTTCCAGCATACGAGAAGTTTGGTAACTTCAGCATCGAATCTGAGTGTCCGAAACTAATCGCTTGGGCTAAGAAGTGTTTGCAGAGGGAAAGTGTGGCTAAGTCCTTGCCTGATCCCGAGAAGGTTATTGGATTCGTGTCTGATCTCAGGAAGAAATTTGGCATTGAG

> T_Unigene_BMK.25288 gi|297844652|ref|XP_002890207.1| 4.17561e-137 gi|297844652|ref|XP_002890207.1| hypothetical protein ARALYDRAFT_471920 [Arabidopsis lyrata subsp. lyrata]

GCTGCGACAATGGGAGAGGAGGTGATTCTTCTGGATTGCGGGGTGAGTATGTTTGGGATGAGGACGAGGATTGCTCTAGCGGAGAAAGGAATCGAGTACGATTATAGAGAAGAAGATCTATGGAACAAGAGCTCCTTGCTCCTCGAGATGAATCCGGTTCATAAGAAAATCCCGGTTCTCATCCACAATGGTAAACCGGTATGTGAATCTCTCATTCAGATCGAGTACATAGACGAGACTTGGCCCCACAAAAACCCTCTCCTTCCTTCCGATCCTTACAAGAGAGCTCATGCCAAATTCTGGGCCGACTTCATCGACAAAAAGGTGAATGTAACGGCGAGGAGGATTTGGGCGACAAAAGGTGATGAGGAAGAAGCATCCAAGGAGTTAATGGAGATACTGAAGACGCTAGAATATGAGCTTGGAGACAAAACTTATTTTGGAGACGAAACGTTTGGGTATGTGGACATAGCTCTCATTGGATTCTATAGCTGGTTTGGAGTGTACGACAAGTTTGGGAATGTCAATATCGAATCAAAGTGTTCGAAATTGATAGCGTGGGCCAAAAGGTGTTTGGAGAGAGAGAGCGTCGCCAAAGCCCTGCCTGAGTCCGAGAAGGTCACTGCGATTATTTCCGAATATAGGAGGAAGAAACTTGGGTTCGAG

**GGP1**

>CL11697.Contig1_All 131 880 minus strand class I glutamine amidotransferase domain-containing protein [Arabidopsis thaliana] >gi|15294152|gb|AAK95253.1|AF410267_1 AT4g30530/F17I23_130 [Arabidopsis thaliana] >gi|20147269|gb|AAM10348.1| AT4g30530/F17I23_130 [Arabidopsis thaliana] >gi|332660377|gb|AEE85777.1| class I glutamine amidotransferase domain-containing protein [Arabidopsis thaliana]

ATGGTGGAGCAGAAAAAGTACTGTCTATTACTAGCGACTCCAGACTCAGAGTTCGTGAAG

AAGACATACGGAGGATACCACAACGTGTTCGTGTCGACATTCGGAGATGAAGGAGAGCAT

TGGGACTCCTTTAGGGTTGTCGAAGGCGTGTTTCCCGATGAGAAAGATCTTGACAAGTAC

GATGGCTTTGTTATCAGCGGAAGCTCTCACGACGCCTTCGAGAATCATGATTGGATCCTT

AAGCTCTGTGATCTTGTCAAGAAACTCGACGAGATGAAGAAGAAAGTTCTTGGCATCTGC

TTTGGTCACCAGATCATAGCTAGAGTAAGGGGAGGAACAGTGGGAAGAGCAAGGAAGGGA

CCAGAACTTAAACTTGGAGACATAACCATCGTGAAGGATGCTATTAAGCCGGGAAGTTAC

TTCGGAAACGAGATTCCGTCGAGCATTGCGATCATCAAATGCCACCAGGACGAAGTGTTG

GTGCTTCCTGAATCTGCTAAACTGCTTGCTTATTCCGACAACTACGAGGTGGAGATGTAC

TCCATTGAGGATCATTTCTTCTGCATTCAAGGTCATCCTGAGTATAACAAAGAGATCCTC

TTCGAGATCGTTGATCGTGTTCTTGGTCTTGGCTACATCAAGCAAGAATTTGCGGATGCT

GCAAAGGCAACGATGGAGAACAGAGGAGCAGACAGGAAGCTTTGGGAGACGGTTTGCAAG

AACTTCCTCAAAGGCAGAACTCCGGCTAAT

> T_Unigene_BMK.16910 gi|15234763|ref|NP_194782.1| 3.67689e-169 gi|15234763|ref|NP_194782.1| defense-related protein, putative [Arabidopsis thaliana]

TCAATCTCTCTCTGTGTGTTTTTTCTTTCGCCGGAGAAAATGGTGGAGCAGAAAAAGTACTGTCTATTACTAGCGACTCCAGACTCAGAGTTCGTGAAGAAGACATACGGAGGATACCACAACGTGTTCGTGTCGACATTCGGAGATGAAGGAGAGCATTGGGACTCCTTTAGGGTTGTCGAAGGCGTGTTTCCCGATGAGAAAGATCTTGACAAGTACGATGGCTTTGTTATCAGCGGAAGCTCTCACGACGCCTTCGAGAATCATGATTGGATCCTTAAGCTCTGTGATATTGTCAAGAAACTCGATGAGATGAAGAAGAAAGTTCTTGGCATCTGCTTTGGTCACCAGATCATAGCTAGAGTAAGGGGAGGAACAGTGGGAAGAGCAAGGAAGGGACCAGAACTTAAACTTGGAGACATAACCATCGTGAAGGATGCTATTAAGCCGGGAAGTTACTTCGGAAACGAGATTCCGTCGAGCATTGCGATCATCAAATGCCACCAGGACGAAGTTTTGGTGCTTCCTGAATCTGCTAAACTGCTTGCTTATTCCGACAACTACGAGGTGGAGATGTACTCCATTGAGGATCATTTCTTCTGCATTCAAGGTCATCCTGAGTATAACAAAGAGATCCTCTTCGAGATCGTTGATCGTGTTCTTGGTCTTGGCTACATCAAGCAAGAATTTGCGGATGCTGCAAAGGCAACGATGGAGAACAGAGGAGCAGACAGGAAGCTTTGGGAGACGGTTTGCAAGAACTTCCTCAAAGGCAGAACTCCGGCTAAT

**SUR1**

>CL12720.Contig1_All 99 1442 ROOTY/SUPERROOT1 [Arabidopsis lyrata subsp. lyrata] >gi|297332132|gb|EFH62551.1| ROOTY/SUPERROOT1 [Arabidopsis lyrata subsp. lyrata]

GTTTTTCCAAAGACTTTTTTTTCTTTTAACCTTCATATAAAATAAGAACT

CAGACCAACGAACAAAACCACATCACATCATCTAAACGAAAAGAGAAGAT

GGGCGAAGAACAACCACACGCCAATCTGGTGGTTCCCGCGTTTACAACTG

AGAAAGATCCCAAGACGCAATCGCAAAATGGCCACAGTAGCGTTTGGCGT

TTCAGTGGAAGCGATAAGGGCGCGAAAGCCTCCACCGTAACGCTGAGAGG

CGTTATCTACATGCTCTTCGGTAACTGCAGCAAAGACGTCAATAAGACCA

TTTTACCCCTCGGCCATGGAGATCCTTCCGTCTACCCTTGCTTCCGCACC

TGTATCGAAGCTGAAGACGCCGTCGTCGACGTTCTCCGCTCCGGCAAAAG

CAACTCTTACTGTCCCGGAGCTGGGATTCTCCCGGCCAGAAGAGCCGTTG

CCGATTACCTGAACCGAGATCTTCCGAACAAGTTAACATCGGAAGACATC

TTCTTAACCGCTGGATGCAACCAAGGGATAGAGATCGTGTTCGATGCGTT

GGCTCGACCAAACGCAAACATCTTGCTTCCACGTCCTGGCTTCCCTCATT

ACGATGCTCGTGCTGTTTACAGTGGTCTTGAGATCCGTAAGTTCGATCTT

CTTCCCAATAAAGAATGGGAGATTGATCTTGAAGGTATTGAAGCCATTGC

AGATGAGAACACTGTGGCAATGGTTGTGATAAACCCCAACAATCCCTGTG

GAAATGTCTACACTCACGACCATCTCAAAAAGGTTGCAGAGACAGCTCGG

AAGCTGGGGATAATGGTGATTACAGACGAAGTGTATGATCAAACTATATT

TGGAGACAATCCCTTTGTTCCAATGGCTAAGTTCGCATCGATTGTTCCTG

TGTTGACCTTTGGAGGCATATCTAAAGGATGGGTTGTTCCTGGATGGAAA

ATTGGCTGGATTGCTTTGAATGATCCCGAGGGCGTTTTCGAGACTACCAA

GGTGGCCCAATCCATCAAACAGATTATTGATATAACTCCTGATCCTGCCA

CTATAATTCAGGCTGCACTTCCCGAGATCCTAGAGAAAGCGGATAAAAAG

TTCTTTGCAAAGAAGAACTTGATATTGAAACATAATGTTGATTTGGTGTG

TGATAGGCTCAAGGACATCCCCTGTGTTGTCTGTCCCAAGAAACCCGAGT

CTTGCACTTACTTATTGACAAAGTTGGAGCTGTCATTGATGGAGAACATC

AAGGATGATATAGATTTCTGCGTGAAGCTGGCCAGAGAGGAGAATCTCGT

GTTTCTACCAGGAGAGGCTTTGGGTTTGAAGAACTGGATGAGGATAACTA

TCGGAGTTGAAGCTCATATGCTTGAGGATGCACTTGATAGACTAAAAGGT

TTCTGTACACGTCATGCCAAGAAGACAGAGACAGAAACCGAGTGATAATA

AATAATGTATTTTACACGCCCCAAGGAACACAGAAGAGGATGTGTAGTGT

ACATTAAATTTTAAGCTAATAAATAAAAAAACTATGGTTCATTTGTCACA

TTGCAATATTGGGAACATGATCTAGC

> T_Unigene_BMK.5984 gi|297836820|ref|XP_002886292.1| 0 gi|297836820|ref|XP_002886292.1| ROOTY/SUPERROOT1 [Arabidopsis lyrata subsp. lyrata]

CCTTCATATAAAATAAGAACTCAGACCAACGAACAAAACCACATCACATCATCTAAACGAAAAGAGAAGATGGGCGAAGAACAACCACACGCCAATCTGGTGGTTCCCGCGTTTTCAACTGAGAAAGATCCCAAGACGCAATCGCAAAATGGCCACAGTAGCGTTTGGCGTTTCAGTGGAAGCGATAAGGGCGCGAAAGCCTCCACCGTAACGCTGAGAGGCGTTATCTACATGCTCTTCGGTAACTGCAGCAAAGACGTCAATAAGACCATTTTACCCCTCGGCCATGGAGATCCTTCCGTCTACCCTTGCTTCCGCACCTGTATCGAAGCTGAAGACGCCGTCGTCGACGTTCTCCGCTCCGGCAAAAGCAACTCTTACTGTCCCGGAGCTGGGATTCTCCCGGCGAGAAGAGCCGTTGCCGATTACCTGAACCGAGATCTTCCGAACAAGTTAACTTCGGAAGACATCTTCTTAACCGCTGGATGCAACCAAGGGATAGAGATCGTGTTCGATGCGTTGGCTCGGCCAAACGCAAACATCTTACTTCCACGTCCTGGCTTCCCTCATTACGATGCTCGTGCTGTTTACAGTGGTCTTGAGATCCGTAAGTTCGATCTTCTTCCCAATAAAGAATGGGAGATTGATCTTGAAGGTATTGAAGCCATTGCTGATGAGAACACTGTGGCAATGGTTGTGATAAACCCCAACAATCCCTGTGGAAATGTCTACACTCACGACCATCTCAAAAAGGTTGCAGAGACAGCTCGGAAGCTGGGGATAATGGTGATTACAGACGAAGTGTATGATCAAACTATATTTGGAGACAATCCCTTTGTTCCAATGGCTAAGTTCGCATCGATTGTTCCTGTGTTGACCTTTGGAGGCATATCTAAAGGATGGGTTGTTCCTGGATGGAAAATTGGCTGGATTGCTTTGAATGATCCCGAGGGCGTTTTCGAGACTACCAAGGTGGCTCAATCCATCAAACAGATTCTTGATATAACTCCTGATCCTGCCACTATAATTCAGGCTGCACTTCCCGAGATCCTAGAGAAAGCGGATAAAAAGTTCTTTGCAAAGAAGAACTTGATATTGAAACATAATGTTGATTTGGTGTGTGATAGGCTCAAGGACATCCCTTGTGTTGTCTGTCCCAAGAAACCCGAGTCTTGCACTTACTTATTGACAAAGTTGGAGCTGTCAATGATGGAGAACATCAAGGATGATATAGATTTCTGCGTGAAGCTGGCCAGAGAGGAGAATCTCGTGTTTTTGCCAGGAGAGGCTTTGGGTTTGAAGAACTGGATGAGGATAACGATCGGAGTTGAAGCTCATATGCTTGAGGATGCACTTGATAGACTAAAAGGTTTCTGTACACGTCATGCCAAGAAGACAGAGACAGAAACCGAG

**UGT74B1**

>CL12550.Contig1_All 86 1468 UDP-glucosyl transferase 74B1 [Arabidopsis lyrata subsp. lyrata] >gi|297339167|gb|EFH69584.1| UDP-glucosyl transferase 74B1 [Arabidopsis lyrata subsp. lyrata]

ATGGCGGAAACAACTAACAAACTCAAACGCCACGTCCTAGTCTTACCATACCCAGTTCAA

GGCCACATTAACCCAATGCTTCAATTCGCTAAACGTCTAGTCTCCAAAAACGTCAAAGTC

ACAATCGCCACCACAACCTACACAGTCTCCTCAATCACAACTCCATCAGTCTCCGTCGAA

CCAATCTCCGACGGATTCGATTCAATCCCCTTAGCTATCCCCGGTTTCAGCGTCGATGTC

TTCTCGGAATCCTTCAAACTCAACGGATCCGAAACCCTAACTCGCCTAATCGAGAAATTC

AAATCCACCGATTCACCAATCGATTGCTTAGTCTACGATTCGTTTCTTCCTTGGGGACTC

GAAGTCGCTAGATCTATGGAGATCTCAGGTGCTTCGTTCTTCACTAACAATCTCTCTGTT

TGTTCTGTGCTTCGTAAATTCGCTAACGGAACTTTTCCTCTTCCCGCCGATCCTGATTCG

GCGCCGTTTTTGACACGTGGCTTGCCGTCGTTGAGTTACGATGAGTTACCTTCGTTTGTT

GGACGTCATTGGTTGACTCATCCTGGGCATGGGAGAGTGCTTCTGGATCAGTTTCCTAAT

CATGAAAATGCTGATTGGTTATTCGTTAATGGCTTTGAAGGCTTAGAAACACAAGATTGT

GAAACTGGTGAATCAGAGGCAATGAGGGCTACGTTGATAGGACCGATGATTCCATCTGCA

TATCTTGATGGTCGTATAAAAGATGATAAAGACTATGGTGCGAGTCTCTTGAAACCGGTT

TCAGAGGAATGTATGAAGTGGCTTGGGACTAAGCCGACTCAGTCTGTAGTGTTTGTCTCG

TTTGGTTCCTTTGGTGTTCTCTTTGAGAAACAACTCGTAGAGATAGCAGTCGCTTTACAA

GAATCGGACTTGAACTTCTTGTGGGTGATTAAAGAAGCTCATGTAGCGAAATTGCCAGAA

GGGTTTGTGGAATCGACTAAAGATAGAGCGTTGTTGGTTTCTTGGGTTAACCAGCTTGAG

GTTTTAGCTCATGAATCGATCGGTTGCTTTTTGACTCATTGTGGTTGGAACTCTACATTG

GAAGGTTTGAGTTTGGGTGTGCCAATGGTGGGTGTGCCTCAGTGGAGTGATCAGATGAAT

GATGCTAAGTTTGTGGAGGAAGTTTGGAAAGTTGGGTATAGAGCGAAAGAGGAAGGTGGT

GAAGCTATTGTGAAGAGTAAAGAGGTGGTGAGGTGTTTGAAAGGAGTGATGAAAGGAGAA

AGTAGTGTGAAGATTAGAGAGAGTTCGAAGAAGTGGAGAGATTTGGCTGTGAAGGCAATG

AGTGAAGGAGGAAGCTCTGATCGGAGTATTAACGAGTTTATAGAGAGTTTAGGGAAGAAA

CAT

> T_Unigene_BMK.12265 gi|297850888|ref|XP_002893325.1| 0 gi|297850888|ref|XP_002893325.1| UDP-glucosyl transferase 74B1 [Arabidopsis lyrata subsp. lyrata]

CTCTTCCGATCTCAAATCTCCCCTTCTCTGTCGTCTTCTTCAATTGAACCAAAACACTCTTATCAAAAGCTCTGCTTCTTTGAGCTCATTACAATGGCGGAAACAACTAACAAACTCAAACGCCACGTCCTCGTCTTACCATACCCAGTTCAAGGCCACATTAACCCAATGCTTCAATTCGCTAAACGTCTAGTCTCCAAAAACGTCAAAGTCACAATCGCCACCACAACCTACACAGTCTCCTCAATCACAACTCCATCAGTCTCTGTCGAACCAATCTCCGACGGATTCGATTCAATCCCCTTAGCTATCCCCGGTTTCAGCGTCGATGTCTTCTCGGAATCCTTCAAACTCAACGGATCCGAAACCCTAACTCGCCTAATCGAGAAATTAAAATCCACCGATTCACCAATCGATTGCTTAGTCTACGATTCGTTTCTTCCTTGGGGACTCGAAGTCGCTAGATCTATGGAGATCTCAGGTGCTTCGTTCTTCACTAACAATCTCTCTGTTTGTTCTGTGCTTCGTAAATTCGCTAACGGAACTTTTCCTCTTCCCGCCGATCCTGATTCGGCGCCGTTTTTGACACGTGGCTTGCCGTCGTTGAGTTACGATGAGTTACCTTCGTTTGTGGGACGTCATTGGTTGACTCATCCTGGGCATGGGAGAGTGCTTCTGGATCAGTTTCCTAATCATGAAAATGCTGATTGGTTATTCGTTAATGGCTTTGAAGGCTTAGAAACACAAGATTGTGAAATTGGAGAATCAGAAGCAATGAGGGCTACGTTGATAGGACCGATGATTCCATCTGCATATCTTGATGGTCGTATAAAAGATGATAAAGACTATGGTGCGAGTCTCTTGAAACCGGTTTCAGAAGAATGTATGAAGTGGCTTGGGACTAAGCCGACTCAGTCTGTAGTGTTTGTCTCGTTTGGTTCCTTTGGTGTTCTCTTTGAGAAACAACTCGTAGAGATAGCAGTCGCTTTACAAGAATCGAACTTGAACTTCTTGTGGGTGATTAAAGAAGCTCATGTAGCGAAATTGCCAGAAGGGTTTGTGGAATCGACTAAAGATAGAGCGTTGTTGGTTTCTTGGGTTAACCAGCTTGAGGTTTTAGCTCATGAATCGGTCGGTTGCTTTTTGACTCATTGTGGTTGGAACTCTACATTGGAAGGGTTGAGTTTAGGTGTGCCAATGGTGGGTGTGCCTCAATGGAGTGATCAGATGAATGATGCTAAGTTTGTGGAGGAAGTTTGGAAAGTTGGGTATAGAGCGAAAGAGGAAGGTGGTGAAGCTATTGTGAAGAGTAAAGAGGTGGTGAGGTGTTTGAAAGGAGTGATGGAAGGAGAAAGTAGTGTGAAGATTAGAGAGAGTTCGAAGAAGTGGAGAGATTTGGCTGTGAAGGCAATGAGTGAAGGAGGAAGCTCTGATCGGAGCATTAACGAGTTTATAGAGAGTTTAGAGAAGAAACAT

**UGT74C1**

>CL12837.Contig1_All 77 1444 UDP-glucoronosyl/UDP-glucosyl transferase family protein [Arabidopsis lyrata subsp. lyrata] >gi|297325182|gb|EFH55602.1| UDP-glucoronosyl/UDP-glucosyl transferase family protein [Arabidopsis lyrata subsp. lyrata]

ATGGGTGAAGGAAAGGGTCACGTACTGTTTTTTCCATATCCATTACAAGGCCACATTAAC

CCAATGATCCAACTCGGCAAACGCTTATCCAAAAAGGGACTCACCGTCACACTTATCATC

GCCTCCAAAGACCACCGTGAACCTTACACCGACGACGAGTACTCCATCACCGTCCAGACC

ATCCACGACGGTTTCTTTCCACACGAACACCCTCACGCCAAGTTCCAAGAAACTCACCGT

TTCAACAAGTCTACTGCTCGTAGTCTCACCGATTTCATCTCTAGAGAGAAGTTATCGGAT

AATCCTCCCAAAGCTTTGATCTATGATCCATTTGTGCCCTTTGCATTGGACGTAGCCAAG

GACTTGGGTCTATACGTAGTGGCCTATTTTACTCAGCCATGGTTGGCTAGTCTTATTTAC

TACCACATCAACGAAGGCACCTACGATGTTCCCATTGATAGACACGACAACCCAATACTC

GCTTCTTTTCCAGCTTTCCCATTGTTAAGCCCAAATGATCTGCCTTCGTTCGCTTGTGAA

AAAGGATCGTACCCTCTTCTCTTCGAATGTGTGGTTAGCCAATTCTCTAATTTTCGGCGA

GCTGATTGCATTCTATGCAACACTTTTGATCAACTTGAACCTAAGGTCTTGAAATGGATG

AATGATCAGTGGCCGGTGAAGAACATTGGACCAGTGGTTCCATCGAAGTTCTTGGATAAC

CGGTTGGCAGAAGACAAAGATTACGACCTCGGGGACTTCAAAACAGAGCCGGACGAGTCT

GTTTTGAGGTGGTTGGGGAATAAGCCGGCGAAATCAGTGGTCTATGTGGCGTTTGGTACG

TTGGTGGCTTTGAGTGAGAAGCAGATGAAGGAAACCGCAATGGCGATTAGACAAACCGGA

TATCACTTCTTGTGGTCTGTTAGAGAATCCGAGAGAAGCAAATTACCGTCTGGATTTATC

GAGGAGGCGTTGGAGAAAGACTGTGGACTTGTGGCTAAGTGGGTTCCTCAGCTGGAGGTT

TTAGCACATGAATCAGTCGGGTGTTTTGTGACACATTGTGGATGGAACTCAACATTGGAG

GCACTGTGCTTAGGGGTTCCATTGGTGGGAATGCCGCAATGGACGGATCAGCCCACAAAC

GCTAAGTTTATAGAGGATGTGTGGAAGATTGGGGTTAGAGTGACGACCGATGGAGAAGGG

TTTACAAGTAAGGAAGAGATTGCAAGATGTGTTGTTGAGGTCATGGAAGGAGAGAATGGA

AAAGAGATTAGGAAGAATGTCGAGAAGCTTAAGGTGTTGGCTCGTGAAGCTATCTCTGAA

GGAGGTAGTTCTGACAAGAACATTGATGACTTTGTTGCTCTTTTGAGT

> T_Unigene_BMK.22372 gi|297822921|ref|XP_002879343.1| 0 gi|297822921|ref|XP_002879343.1| UDP-glucoronosyl/UDP-glucosyl transferase family protein [Arabidopsis lyrata subsp. lyrata]

CATATCATCATCCACTTAGTGTTATTTTTATTTTTTTTTGGCTTCGAACAAATGGGTGAAGGAAAGGGTCACGTACTGTTTTTTCCATATCCATTACAAGGCCACATAAACCCAATGATCCAACTCGGCAAACGCTTATCCAAAAAGGGACTCACCGTCACACTTATCATCGCCTCCAAAGACCACCGTGAACCTTACACCGACGACGAGTACTCCATCACCGTCCAGACCATCCACGACGGTTTCTTTCCACACGAACACCCTCACGCCAAGTTCCAAGAAACTCACCGTTTCAACAAGTCTACTGCTCGTAGTCTCACCGATTTCATCTCTAGAGAGAAGTTATCGGATAATCCTCCCAAAGCTTTGATCTATGATCCATTTGTGCCCTTTGCATTGGACGTAGCCAAGGACTTGGGTCTATACGTAGTGGCCTATTTTACTCAGCCATGGTTGGCTAGTCTTATTTACTACCACATCAACGAAGGCACCTACGATGTTCCCATTGATAGACACGACAACCCAATACTCGCTTCTTTTCCAGCTTTCCCATTGTTAAGCCCAAATGATCTGCCTTCGTTCGCCTGTGAAAAAGGATCGTACCCTCTTCTCTTCGAATGTGTGGTTAGCCAATTCTCTAATTTTCGGCGAGCTGATTGCATTCTATGCAACACTTTTGATCAACTTGAACCTAAGGTCTTGAAATGGATGAATGATCAGTGGCCGGTGAAGAACATTGGACCAGTGGTTCCATCGAAGTTCTTGGATAACCGGTTGCCAGAAGACAAAGATTACGACCTCGGGGATTTCAAAACAGAGCCGGACGAGTCTGTTTTGAGGTGGTTGGGGAATAAGCCGGCGAAATCAGTGGTCTATGTGGCGTTTGGTACGTTGGTGGCTTTGAGTGAGAAGCAGATGAAGGAAACCGCAATGGCGATTAGACAAACCGGATATCACTTCTTGTGGTCTGTTAGAGAATCCGAGAGAAGCAAATTACCGTCTGGATTTATCGAGGAGGCGTTGGAGAAAGACTGTGGACTTGTGGCTAAGTGGGTTCCTCAGCTGGAGGTTTTAGCACATGAATCAGTCGGGTGTTTTGTGACACATTGTGGATGGAACTCAACATTGGAGGCACTGTGCTTAGGGGTTCCATTGGTGGGAATGCCTCAATGGACGGATCAGCCCACAAACGCTAAGTTTATAGAGGATGTGTGGAAGATTGGGGTAAGAGTGAAGACGGATGGAGAAGGGTTTACGAGTAAGGAAGAGATTGCAAGATGTGTTGTTGAGGTCATGGAAGGAGAGAATGGAAAAGAGATTAGGAAGAATGTCGAGAAGCTTAAGCTGTTGGCTCGTGAAGCTATCTCTGAAGGAGGTAGTTCTGACAAGAACATTGATGACTTTGTTGCTCTTTTGTGT

**SOT18**

>CL13054.Contig1_All 73 1107 sulfotransferase 18 [Arabidopsis thaliana] >gi|75169683|sp|Q9C9C9.1|SOT18_ARATH RecName: Full=Sulfotransferase 18; Short=AtSOT18; AltName: Full=Desulfoglucosinolate sulfotransferase B; Short=AtST5b >gi|332197428|gb|AEE35549.1| sulfotransferase 18 [Arabidopsis thaliana]

ATGGCATCAGAAGCCGTAGTAACCGCCACGACCGTACCGAACCACGACAAGACCGAGACA

GAGTCAACAGAATTCGAGAAGAATCAGAAACGGTACCAAGACCTAATCTCCACGTTTCCT

CACGAGAAAGGCTGGAGACCTAAAGAGCCCTTGATCGAGTACGGTGGTTACTGGTGGCTA

CAGCCTCTCCTCGAAGGTTGTATTCACGCGCAAGAGTTCTTTCAAGCACGACCCAGTGAC

TTCCTCGTCTGTAGCTACCCAAAGACAGGTACCACTTGGCTCAAAGCCCTAACTTTCGCC

ATCGTCAATCGATCCCGCTTCGACGATTCCTCGAACCCTCTCCTAAAACGTAACCCTCAC

GAGTTTGTTCCATACATTGAGATCGATTTCCCTTTCTTCCCTGAAGTAGATGTTCTCAAA

GACAAAGGTAACACTCTGTTTTCGACTCATATCCCACATGAGTTATTACCCGATTCGGTT

GTGAAATCTGGTTGTAAGATGGTTTACATCTGGAGAGATCCAAAGGACACTTTTATCTCC

ATGTGGACTTTTCTCCACAAGGAAAGGTCAGAACTTGGACCTATCAACAATCTTGAGGAG

TCTTTTGATATGTTCTGTCTTGGTCAGTCTGGGTACGGTCCTTATCTTAATCATGTCTTG

GCGTATTGGAAAGCTTACCAAGAAAATCCAGATCGGATTTTGTTCCTCAAGTATGAGACT

ATGAGAGCAGATCCTTTGCCGTATGTGAAGATATTGGCTGAGTTTATGGGCCATGGATTC

ACAGCGGAGGAAGAGGAGAAAGGTATTGCTGAGAAAGTGGTGAATCTTTGCAGCTTCGAG

ACATTGAAGAATCTTGAAGCTAACAAAGGGGAAAAAGACAGAGAGGATCGTCCTGGTGTC

TATGCGAATAGCGCGTATTTCAGGAAAGGAAAGGTGGGAGATTGGTCGAATTATCTGACT

CCAGAGATGGCAGCTCGTATTGATGGGATAATGGAAGAGAAATTTAAGGGCACCGGCTTG

CTTGAACATGGTAAA

> T_Unigene_BMK.12426 gi|15221130|ref|NP_177549.1| 0 gi|15221130|ref|NP_177549.1| SOT18 (DESULFO-GLUCOSINOLATE SULFOTRANSFERASE 18); 3-methylthiopropyl-desulfoglucosinolate sulfotransferase/ 4-methylthiobutyl-desulfoglucosinolate sulfotransferase/ 5-methylthiopentyl-desulfoglucosinolate sulfotransferase/ 7-methylthioheptyl-desulfoglucosinolate sulfotransferase/ 8-methylthiooctyl-desulfoglucosinolate sulfotransferase/ desulfoglucosinolate sulfotransferase/ indol-3-yl-methyl-desulfoglucosinolate sulfotransferase/ sulfotransferase [Arabidopsis thaliana]

GAAGACAGACCACTCCCACACACACACAAGCCTTCAAATCCTCTTTCTCTATCTCCAATGGCATCAGAAGCCGTAGTAACCGCCACGACCGTACCGAACCACGACAAGACCGAGACAGAGTCAACAGAATTCGAGAAGAATCAGAAACGGTACCAAGACCTAATCTCCACGTTTCGTCACGAGAAAGGCTGGAGACCTAAAGAGCCCTTGATCGAGTACGGTGGTTACTGGTGGCTACAGCCTCTCCTCGAAGGTTGTATTCACGCGCAAGAGTTCTTTCAAGCACGACCCAGTGACTTCCTCGTCTGTAGCTACCCAAAGACAGGTACCACTTGGCTCAAAGCCCTAACTTTCGCCATCGTCAATCGATCCCGCTTCGACGATTCCTCGAACCCTCTCCTAAAACGTAACCCTCACGAGTTTGTTCCTTACATTGAGATCGATTTCCCTTTCTTCCCTGAGGTAGATGTTCTCAAAGACAAAGGTAACACTCTGTTTTCGACTCATATCCCACATGAGTTATTACCCGATTCGGTTGTGAAATCTGGTTGTAAGATGGTTTACATCTGGAGAGATCCAAAGGACACTTTTATCTCCATGTGGACTTTTCTCCACAAGGAAAGGTCAGAACTTGGACCTATCAACAATCTTGAGGAGTCTTTTGATATGTTCTGTCTTGGTCAGTCTGGGTACGGTCCTTATCTTAATCATGTCTTGGCGTATTGGAAAGCTTACCAAGAAAATCCTGATCGGATTTTGTTCCTCAAGTACGAGACTATGAGAGCTGATCCTTTGCCATATGTGAAGACACTAGCTGAGTTTATGGGCCATGGATTCACAGCGGAGGAAGAGGAGAAAGGTATTGCTGAGAAAGTGGTGACTCTTTGCAGCTTCGAGACATTGAAGAATCTTGAAGCTAACAAAGGGGAGAAAGACAGAGAGGATCGTCCTGGTGTCTATGCGAATAGCGCGTATTTCAGGAAAGGAAAGGTTGGAGATTGGTCGAATTATCTGACTCCAGAGATGGCAGCTCGTATTGATGGATTAATGGAAGAGAAATTTAAGGGCACCGGCTTGCTTGAACATGGTAAA

**SOT17**

>CL19111.Contig1_All 53 754 minus strand sulfotransferase 17 [Arabidopsis thaliana] >gi|75173378|sp|Q9FZ80.1|SOT17_ARATH RecName: Full=Sulfotransferase 17; Short=AtSOT17; AltName: Full=Desulfoglucosinolate sulfotransferase C; Short=AtST5c >gi|332191611|gb|AEE29732.1| sulfotransferase 17 [Arabidopsis thaliana]

ATGGAATCCAAAACTCTAAACGACGTCATTGCGTCAGAGTGTAATCACGACTCGACCGAG

TTAGTATCTTCGTCGCCATCAGAATTCGAGAAGAACCAAAAACATTACCAAGAAATCATC

GCTACGCTTCCTCACAAAGATGGCTGGAGACCAAAAGATCCGTTCGTCGAGTACGGTGGT

CACTGGTGGCTACAACCTCTGCTTGAAGGTTTACTTCACGCTCAGAGATTCTTCAAAGCA

CGACCCAGTGATTTCTTCGTCTGCAGCTACCCAAAAACCGGCACGACTTGGCTCAAAGCC

TTAACTTTCGCAATCGCGAACCGTACCAAGTTCGAAGATTCCACAAACCCTCTTCTCAAA

CGTAACCCACACGAGTTTGTTCCTTACATCGAAATCGATTTCCCGTTTTTCCCGAGCGTT

GATGTTCTTAACGACGAAGGAAACACGCTTTTCTCAACTCATATCCCTTACGCTCTCTTA

CCTGAATCAATCTTGGAATCAGATTTTAAGATGGTTTACATCTGGAGAGACCCAAAAGAC

ACATTTGTCTCCATGTGGACTTTTTCTCATAAGGAGAGATCACAACAAGGATCACTCATT

AGCCTCGAGGAAGCTTTTGATAAGTATTGTCAAGGCTTATCTGTGTACGGTTCTTATCTA

GATCATGTTTTAGGGTATTGGAAAGCTTACGTAGCAAACCCA

> T_Unigene_BMK.8283 gi|15221820|ref|NP_173294.1| 8.38771e-170 gi|15221820|ref|NP_173294.1| SOT17 (SULFOTRANSFERASE 17); desulfoglucosinolate sulfotransferase/ sulfotransferase [Arabidopsis thaliana]

AATCTCTCTACAATGGAATCCAAAACTCTAAACGACGTCATTGGGTCAGAGTCTAATCACGACTCGACCGAGTTAGTATCGTCGTCGCCATCAGAATTCGAGAAGAACCAAAAACATTACCAAGAAATCATCGCTACGCTTCCTCACAAAGATGGCTGGAGACCAAAAGATCCGTTCGTCGAGTACGGTGGTCACTGGTGGCTACAACCTCTGCTCGAAGGTTTACTCCACGCTCAGAGATTCTTCAAAGCACGACCCAGTGATTTCTTCGTCTGCAGCTATCCAAAAACCGGCACGACTTGGCTCAAAGCCTTAACTTTCGCAATCGCGAACCGTACCAAGTTCGAAGATTCCACAAACCCTCTTCTCAAACGTAACCCACACGAGTTTGTTCCTTACATCGAAATCGATTTCCCGTTTTTCCCAAGCGTTGATGTTCTTAAAGACGAAGGAAACACGCTTTTCTCAACTCATATCCCTTACGCTCTCTTACCTGAATCAATCTTGGAATCAGATTGTAAGATGGTTTACATATGGAGAGACCCAAAGGACACATTTGTCTCCATGTGGACTTTTTCTCATAAGGAGAGATCACAACAAGGATCACTCATTAGCCTCGAGGAAGCTTTTGATAAGTATTGTCAAGGCTTATCTGTGTACGGTTCTTATCTAGATCATGTTTTAGGGTATTGGAAAGCTTACGAAGCAAACCCAAATCAGATTTTGTTTCTTAAGTACGAGACTATGAGAGCTGAT

**SOT16**

>CL14695.Contig1_All 288 785 minus strand sulfotransferase family protein [Arabidopsis lyrata subsp. lyrata] >gi|297334805|gb|EFH65223.1| sulfotransferase family protein [Arabidopsis lyrata subsp. lyrata]

ATGGAATCAAACACGACCCAAAACGGATCCGAAGTCCAACTCACAGAGTTCGAAAAAACC

CAGAAGAAGTACCACGATCTCATCGCTACACTTCCAAAGAGCAAAGGCTGGAGACCAGAC

GAGATCTTAACCCAATATGGTGGACACTGGTGGCAACAATGTCTCCTCGAAGGTCTTTTT

CACGCCAACGACCATTTCGAAGCACGACCAACTGATTTCCTCGTCTGTAGCTATCCTAAA

ACCGGTACGACTTGGCTCAAAGCACTAACGTACGCCATCGTTAACCGATCCCGATTCGAT

GACGCAACGAACCCTCTCCTCAAACGTAACCCTCACGAGTTCGTGCCTTACGTTGAGATC

GATTTCGCGTTTTACCCAACCGTTGATGTTCTTCAAGACAGAAAGAACCCGCTTTTCTCT

ACCCATATTCCAAACGGGTCATTACCCGATTCGATTGTGAAGTCGGGTTGTAAAATGGTG

TACATTTGGAGAGATCCT

>Unigene1796_All 93 590 minus strand sulfotransferase family protein [Arabidopsis lyrata subsp. lyrata] >gi|297334805|gb|EFH65223.1| sulfotransferase family protein [Arabidopsis lyrata subsp. lyrata]

ATGGAATCAAACACGACCCAAAACGGATCCGAAGTCCAACTCACAGAGTTCGAAAAAACC

CAGAAGAAGTACCACGATCTCATCGCTACACTTCCAAAGAGCAAAGGCTGGAGACCAGAC

GAGATCTTAACCCAATATGGTGGACACTGGTGGCAACAATGTCTCCTCGAAGGTCTTTTT

CACGCCAACGACCATTTCGAAGCACGACCAACTGATTTCCTCGTCTGTAGCTATCCTAAA

ACCGGTACGACTTGGCTCAAAGCACTAACGTACGCCATCGTTAACCGATCCCGATTCGAT

GACGCAACGAACCCTCTCCTCAAACGTAACCCTCACGAGTTCGTGCCTTACGTTGAGATC

GATTTCGCGTTTTACCCAACCGTTGATGTTCTTCAAGACAGAAAGAACCCGCTTTTCTCT

ACCCATATTCCAAACGGGTCATTACCCGATTCGATTGTGAAGTCGGGTTGTAAAATGGTG

TACATTTGGAGAGATCCT

> T_Unigene_BMK.10319 gi|297842165|ref|XP_002888964.1| 0 gi|297842165|ref|XP_002888964.1| sulfotransferase family protein [Arabidopsis lyrata subsp. lyrata]

CATCTCTCAAATAATCTCAATTCGAGCTTTCCAATTATATCTTCTAACTCAATCAATCCAAACTCATCCGCAATGGAATCAAACACGACCCAAAACGGATCCGAAGTCGAACTCACAGAGTTCGAAAAAACCCAGAAGAAGTACCACGATCTCATCGCTACACTTCCAAAGAGCAAAGGCTGGAGACCAGATGAGATCTTAACCCAATATGGTGGACACTGGTGGCAACAATGTCTCCTCGAAGGTCTTTTTCATGCCAACGACCATTTCGAAGCACGACCAACTGATTTCCTCGTCTGTAGCTATCCAAAAACCGGTACGACTTGGCTCAAAGCACTAACGTACGCCATCGTTAACCGATCCCGATTCGATGACGCAACGAACCCTCTCCTCAAACGTAACCCTCACGAGTTCGTGCCTTACGTTGAGATCGATTTCGCGTTTTACCCAACCGTTGATGTTCTTCAAGACAGAAAGAACCCGCTTTTCTCTACCCATATTCCAAACGGGTCATTACCCGATTCGATTGTGAAGTCGGGTTGTAAAATGGTGTACATATGGAGAGATCCAAAGGATACTTTCATCTCCATGTGGACTTTTCTCCATAAGGAGAAGTCTCAAGAAGGTCAATTAGCGAGTCTTGAAGAGAGCTTTGATATGTTTTGCAAAGGTTTATCTGTGTATGGTCCTTATCTGGATCATGTTTTAGGTTATTGGAAAGCTTACCAAGAGAATCCAGATAGGATTTTGTTTCTTAGGTATGAGACTATGAGAGCTAATCCTTTGCCGTTTGTGAAGAGATTGGCTGAGTTTATGGGTTATGGATTCACGGATGAGGAAGTGGAGAAAGGTGTTGCTGAGAAAGTGGTGAATCTT

**FMO-GSOX1**

>CL18160.Contig1_All 2 409 FMO-like protein [Eutrema halophilum]

GTGGCAGTAATCGGAGCCGGAGCAGCCGGTCTCGTAACAGCTAGGGAGCTCCGTCGTGAA

GGTCACACTGTTGTAGTCTTTGACAGGGAGAAACAAGTGGGAGGTCTCTGGATCTACTCA

CCTAAAGCTGATTCTGACCCGCTTAGCCTTGACCCGACCCGATCCATAGTCCACTCGAGC

ATCTACGAGTCTCTTCGAACCAACCTACCAAGAGAAAGCATGGGTTTCACAGACTATCCA

TTCGTGCCACGTGGCGATGACTTGTCAAGAGACACGAGACGGTATCCGAGTCACAGGGAA

GTTCTTGCGTACCTTCAAGACTTTGCTAGAGAGTTTAAAATAGAGGAGATGGTTCGGTTC

GAGACTGAGGTGGTTCGGGTTGAACCGGTTAACGGGAAATGGAGTGTC

> T_Unigene_BMK.12547 gi|15218834|ref|NP_176761.1| 4.14262e-134 gi|15218834|ref|NP_176761.1| FMO GS-OX1 (FLAVIN-MONOOXYGENASE GLUCOSINOLATE S-OXYGENASE 1); 3-methylthiopropyl glucosinolate S-oxygenase/ 4-methylthiopropyl glucosinolate S-oxygenase/ 5-methylthiopropyl glucosinolate S-oxygenase/ 6-methylthiopropyl glucosinolate S-oxygenase/ 7-methylthiopropyl glucosinolate S-oxygenase/ 8-methylthiopropyl glucosinolate S-oxygenase/ monooxygenase [Arabidopsis thaliana]

TCGGAAACTATGCCAGTGGTGCCGATATTAGTAGGGACACAGCTAAGGTTGCGAAAGAAGTTCACATTGCGTCTTAGAGCGAATGAATCTAATACATATGAGAAGATTCTAGTGCCCCAGAACAATCTATGGATTCATTCAGAGATAGACTTCGCTAATGAAGATGGATCAATTGTTTTCAAAAACGGGAAGGTGGTATATGTTGATACCATTGTGCATTGCACCGGGTATAAATATTACTTCCCATTTCTTGAAACCAATGGTTATATGAGCGTTGATGAAAACCGAGTTGAACATCTATACAAGCATGTCTTTCCACCCGCGCTAGCCCCTGGACTTTCTTTCGTCGGTTTGCCAGGAATGGCCATACAGTTTGTTATGTTTGAAATTCAAAGTAAATGGGTGGCTCGAGTCTTGTCAGGACGAGTTATACTTCCCTCGCAAGAAAAGATGATGGAAGATGTTAGTTCGTGGTATGCATCACTTGATGTGTTAGGACTTCCCAAAAGATTTACTCATAAATTGGGTAAAATTACGCGTGAGTACCTCAATTGGATCGCGGAAGAATGTCGTGGTCCGCTCGTTGAAAATTGGAGAATTCGACAAGTTGAACGAGGATTCGAGAGGATGGTCACCCACGCAGAAGTGTACCGCGATGAATGGGATGATGATCATCTCATGGAAGAAGCATACGAGGATTTCGCTAGGAAGAAGTTGATTAGTTTGCATCCTTCTCATTTTCTCGAAATGTATGGA

**FMO-GSOX5**

>CL5158.Contig1_All 1 693 flavin-monooxygenase glucosinolate S-oxygenase 5 [Arabidopsis thaliana] >gi|332190721|gb|AEE28842.1| flavin-monooxygenase glucosinolate S-oxygenase 5 [Arabidopsis thaliana]

AAAGAAGTTCATATCGCGTCTAGATCGAATCCATCTGAGACATACGAAAAGCTTCCCGGG

TCCAACAATCTATGGCTTCACTCTATGATAGAAAGCGCGCGGGAAGATGGATCGATTGTT

TTCCAGAACGGTAAGGTTGTACAAGCTGATACCATTGTACATTGCACCGGTTACAAATAT

CACTTCCCGTTTCTCGATACCAATGGCTATCTTACCGTCGAGGATAACTGTGTTGGACCG

CTTTACAAACATGTGTTTCCGCCCGCACTTGCTCCAGGGCTTTCCTTCGTTGGTTTACCA

TGGATGACACTTCAATTTTTCATGTTTGAGCTCCAAAGCAAGTGGGTGGCTGCAGTTTTG

TCCTGTGGGGTTTCACTTCCTTCAGAAGACAAAATGATGGAAGATGTTATCGCCTTCTAT

GCAGAGCGTGATGCAAACGGGATTCCCAAGAGATACGCACATAAGCTTGGTGGGTCTCAA

GTTGAATACCTCAACTGGATAGCCGAACAAATTGGTGCCTTGCCGCTTGAACAATGGAGA

GCTGAGGAAGTAGAAGGCGGCTATCGAAGACTTGCCACACAATCAGACACTTTCCGCGAT

AAGTGGGATGATGATCATCTCATTGTTGAGGCTTATGATGATTTCTTGAGACAAAAGCTG

ATTAATGTTCTTCCTTCTAAGTTGTTGAAATCC

>CK_Unigene_BMK.19786 gi|42561939|ref|NP_172678.3| 0 gi|42561939|ref|NP_172678.3| FMO GS-OX5 (FLAVIN-MONOOXYGENASE GLUCOSINOLATE S-OXYGENASE 5); 8-methylthiopropyl glucosinolate S-oxygenase/ flavin-containing monooxygenase/ monooxygenase [Arabidopsis thaliana]

AAAACAAAGAAAAAAATGGCACCCGCACGGAGCAAAATCAACTCAGTCCACGTGGCAGTGATCGGAGCCGGAGCAGCCGGACTTGTAGCTGCACGTGAGCTACGCCGCGAAAATCACTCAGTCGTCGTCTTTGAACGCAACTCAAAAGTCGGAGGTCTCTGGGTATACACACCTGATAGTGAGCCAGATCCACTTAGCCTCGATCCAAATCGAACCATAGTCCACTCAAGCGTCTACGATTCTCTCCGTACAAACCTCCCACGAGAGTGTATGGGTTACAGAGACTTCCCTTTCGTGCCTCGACCTGAAGACGACGAATCTAGAGACCCGAGAAGGTATCCTAGTCACAGAGAAGTCCTCGCTTACCTTGAAGACTTCGCGAGAGAATTCAAACTCGTGGAGATGGTTCGATTCGAGACTGAAGTGGTTTGTGTTGAGCTTGAAGATCCAAAATGGAGGATTCAGTTCAGAAGTTCAGATGGAATCTCCGGTGATGAGATCTTTGATGCCGTCGTTGTTTGTAATGGACATTTTACAGAGCCTCGAGTTGCTCATGTTCCTGGTATAGATTCATGGCCAGGGAAACAAGTTCATAGCCACAATTATCGTGTTCCTGATCCATTCAAAGACCAGGTGGTGGTAGTGATAGGAAATTTTGCTAGTGGATCCGATATCAGTAGGGATATAACGGGAGTGGCGAAAGAAGTTCATATCGCGTCTAGATCGAATCCATCTGAGACATGCGAAAAGCTTCCCGGGTCCAACAATCTATGGCTTCACTCTATGATAGAAAGCGCGCGGGAAGATGGATCGATTGTTTTCCAGAACGGTAAGGTTGTACAAGCTGATACCATTGTACATTGCACCGGTTACAAATATCACTTCCCGTTTCTCAACACCAATGGCTATCTTACCATCGAGGATAACTGTGTTGGACCGCTTTACAAACATGTGTTTCCGCCCGCACTTGCTCCAGGGATTTCCTTCGTCGGTTTACCATGGATGACACTTCAATTTTTCATGTTTGAGCTCCAAAGCAAGTGGGTGGCTGCAGTTTTGTCCGGTGGGGTTTCACTTCCTTCAGAAGACAAACTGATGGAAGATGTTACCGCCTTCTATGCAGAGCGTGATGCAAACGGGATTCCCAAGAGATACGCACATAAGCTTGGTGGGTCTCAAGTTGAATACCTCAACTGGATAGCCGAACAAATTGGTGCATTGCCGCTTGAACAATGGAGAGCTGAGGAAGTAGAAGGCGGCTATCGAAGACTTGCCACACAATCAGACACTTTCCGCGATAAGTGGGATGATGATCATCTCATTGTTGAGGCTTATGATGATTTCTTGAGACAAAAGCTGATTAATGTTCTTCCTTCTAAGTTGTTGAAATCCAGGAAAATGATGAAT

**GS-OH**

>CL12207.Contig1_All 142 1215 RecName: Full=Probable 2-oxoacid dependent dioxygenase

CGTGTACTCGGTTTGATGCTTTAACCCGTTCTCCTCTCCTTATATATAGA

GAGACAGAGAGGTCTCTGACTCAAAACAGAGCCAAAGTTTCTCTATTTCC

TCTGTTTCTTGAAAGAAAGATCGATGGACTCTGAGGATACAATGGCTGAA

ATATACGACCGTGCTAGTGAGTTAAAAGCCTTCGACGAGACGAAGACCGG

AGTGAAGGGTCTTGTAGAGACTGGAATCACAAAAATCCCTCGCATTTTCC

ATAACCCGCTTGTCACAGAAACAACCAGTAAACCTGGCTCAAGGGTGACG

TTCCCAGAAATCGATCTAGGAGGTGGCGTGTTGGAATCCCCGGCCATGCG

AGAGAGGGTGGTTGATGAGATTAAATACGCGATGGAGAAGTATGGGTTTT

TCTACGCGGTTAACCATGGGATTCCACTACATGTGATGGAGAAGATGAAA

GATGGCGTTCGTAGGTTTCACGAGCAAGATCCTGAAGTGAGGAAAATGTT

CTACACCCGAGACAAAACCAAAAAAGTTAGGTATAACTCAAATGCTGATC

TCCATGAATCTCCTGCTGCGAGTTGGAAAGATACTTTGACTACGATAATG

GCTCCTGATGCTCCAAAGGCAGAGGAGTTGCCAAAGGTTTGCGGGGAGAT

CATGTTGGAGTACTCAAAGGAAGCGATGAAGTTAGCAGAGTTAATCTTTC

AACTTATATCAGAAGCTTTAGGGTTGAGTTCTAACCACCTCAAAGAAATG

GATTGCACAAAAGGTTTAGTCATGCTCAATCTTTACTACCCGCCCTGTCC

TGAGCCAAATCTAACATTAGGGGGCGCTCCTCACACGGACAGATCTTTCA

TCACTATCCTTCTTCAAGACCACATTGAAGTATTTCAAGTTTTCCGTGAT

GGATCCTGGATCGATGTTGCTCCTAATCCCAAAGCTCTTCTCATTAACGT

TGGAGATCTCCTACAGCTTATATCGAATGACAAGTTTATAAGTGTGGAGC

ATAGGGTTTTGGCAAATAGACATCAAGAGCCGCGCATTTCGATCGCGTGT

TTCTTCGTGCATCCTTTCCCAGGTTCAAGAAAATATGGACCCATTAAAGA

GATTTTGTCTGAACAAAACCCTCCCAAGTACAGAGAGACCACCGCGGAAA

CCTCTAGCCACTATGTGGCTAGACAACTTGATGGGAAGAATGCTTCGTTG

CTTCATTTAAGGATCTGAAAAATATGGTGAACCCAAATCTCCTAAGTTTT

CTTGAGTTTCCAATAAGATATTAGTGCACACAGATGCACCAATAAGGTTC

ATATAAATACCACATGTTCTAGTCCTAGTAAGATGTCTATGCCAATTAAA

TTTGGTTTATGATGTTATTGTCTATCCAATAATTTTTGTATTTTCTAAAC

CATAAGTATCAATCAATAATGCTTGAGTTGTGAGTTTCAATGCATGTTTC

TTCGGATATAATAAACGCTACAATATGATCTAAGAGCATGTTTAACGTTG

ATGACTTGGTGATCTGACCCACCCAAAAGTCA

> T_Unigene_BMK.14596 gi|15224730|ref|NP_180115.1| 0 gi|15224730|ref|NP_180115.1| 2-oxoglutarate-dependent dioxygenase, putative [Arabidopsis thaliana]

AGGAACATCAAGTTTCCATCGAGAAAACCAAAAGAAAAAAAAGAAAAAAGGTTGATGGAGTCGTACGATCGTGCCAGTGAGTTAAAAGCATTCGACGAGACAAAGATCGGCGTGAAAGGTCTTGTCGAAGCTGGAATAACAAAAATCCCACGCATTTTCCATGATCCGCGAGCTACCTCAAGAAACACTAAACCATCCTCAATGATGGTGACGATTCCAACAATCGATATGAGTGGCGTTTTCGAATCCATGGACACAAGAAAGAGTGTGGTTGCAAAGGTGAAAGAGGCAACGGAGAAGTTTGGGTTTTTCCAGGCGATAAACCATGGGATTCCTATGGAACTTATGGAGAAAATGATAGATGTGACTCGTCGGTTTCACGAGCAAGATCCAGAAGTGAGGAAAACGTTCTATAGCCGAGACAAAACCAAACAGTTTAAGTATAACTCTAATAATGATCTCTTTGGCTCACCTGCTGCGGGTTGGAGAGATACTTTCACTTGTTTTATGGCTCCTAATGTTCCAAAACTAGAGGACTTACCAAAGATTTGTGGGGAGGTCATGTTGGAGTACTCAAAGGAAGTGATGAAGTTTGGGGAGTTAATCTTTGAACTTCTTTCAGAAGCTTTAGGTTTGAATCCTAACCATCTCAAAGATATGGATTGCGCAGAAGGTTTAATGCTGCTATGTCATTTCTACCCGCCTTGTCCTGAGCCCGACCGAACACTCGGCGGCACTCCTCACACAGACAGATCTTTCCTCACTATTCTTCTTCAGGACCACATTGGAGGACTTCAAGTTCTCCAGGATGGATACTGGATCGATGTTCCTCCTACTCCGGGAGCTCTTATCCTTAACGTAGGAGATCTTATACAGCTTTTAACAAATGACAAGTTTGTAAGTGTGGAGCATAAAATTCTGGCAAACGCAGGTCAAGAGCCACGCATCTCTATCGCGTCTTTCTTTATCCATCCACCGACAAGTTCGAAAGTATATGGACCCATTAAAGAGCTTCTGTCGGAAGAAAACCCTCCCAAGTACAGAGACTCCACTGCGGAAGTCTCCAACCACTTCGTGGATAGAAAACCTAATGTGAATAATTCGTTGAGCCATTTAAGGATC

**CYP81F2**

>CL2102.Contig1_All 29 1528 cytochrome P450 monooxygenase CYP91A2 [Arabidopsis thaliana]

ATGTTGTACTTCATTCTTCTCCCTCTTCTGTTCCTTGTTTTAACTTACAAATTCCTCTAC

TCCAAAACGCACCGTTACAAGCTTCCTCCGGGACCACCATCACGTCTCTTCGTCGGCCAT

CTCCACCTCATGAAACCACCGATCCACCGTCTCCTCCAACGCTTCTCCAACCAATACGGC

CCAATCTTCTCCCTCCGTTTCGGCTCCCGACAAGTCGTGGTCATCACTTCCCCTTCCCTC

GCTCAAGAAGCCTTCACCGGCCAAAACGACATCATCCTCTCTAGCCGGCCACTTCAGCTC

ACCGCCAAATACGTCGCTTATAACCACACCACCGTCGGAACCGCTCCATACGGTGACCAT

TGGCGTAACCTCCGCCGAATTTGCTCCCAAGAGATTCTCTCCTCTAACCGTCTCATCAAT

TTCCAACACATCCGCAAAGACGAGATCTTACGGATGATCACGCGCCTCTCACGTGACACA

CAAACCTCTAACGGAACCAGATGTTTCACTCACGTCGAGCTTGAACCTCTCTTATCCGAT

TTAACGTTCAACAACATAGTAAGGATGGTCACAGGGAAGAGATATTACGGCGACGACGTC

AACAACAAGGAAGAAGCAGAGCTTTTCAAGAAGCTCGTTTATGATATCGCCATGTATAGT

GGCGCGAATCATTCCGCTGATTACTTGCCGGTGCTGAAACTATTCGGAAACAAATTCGAG

AAACGAGTTAAAGCTCTGGGCAAATCCATGGATGATATTTTGCAACGTTTGCTTGATGAG

TGTAGAAGAGATAAAGAAGGTAACACAATGGTGAATCATTTGCTTTCTCTACAACAACAA

GAACCTGAGTATTACACTGACGTTATCATCAAAGGCCTTATGATGTCGATGATGCTCGCC

GGGACTGAGACCTCCGCCGTGACTCTAGAGTGGGCGATGGCGAATTTGTTGAGAAATCCA

GAAGTATTGGAGAAGGCGAGATCGGAGATCGATGAGAAGATCGGGAAAGATCGTCTGATC

GATGAATCAGACATCGCCGTCCTTCCTTATCTCCAAAACGTCGTCTCCGAAACATTCCGG

CTATTTCCTGTGGCGCCGTTTTTAATCCCTCGCACGACGACGGAGGATATGAAAATCGGC

GGTTACGATGTCCCGCGCGATGCGATAGTGTTGGTTAACGCTTGGGCTATACACAGAGAT

CCAGTGCTGTGGGAAGATGCAGAGAAGTTTAATCCGGATCGGTTTATCGACGGATGTGTA

AATGAGTACAACGTTTACAAGCTGATGCCGTTTGGAAACGGTCGGAGAACTTGTCCCGGC

GCCGGATTAGGGCAGAGGATCGTGACGTTGGCGTTGGGAACGTTGATTCAGTGCTTTGAT

TGGGAGAATGTAAAAGGCGAAGAGATGGATATGTCAGAGAGTGCTGGGTTAGGTATGCGT

AAGATGGATCCTTTACGGGCCATGTGCAGGCCTAGGCCCATTATTGCTAAGCTTCTAATC

> T_Unigene_BMK.5602 gi|21618274|gb|AAM67324.1| 0 gi|21618274|gb|AAM67324.1| cytochrome P450 monooxygenase CYP91A2 [Arabidopsis thaliana]

AAAATGTTGTACTTCATTCTTCTCCCTCTTCTGTTCCTTGTTTTAACTTACAAATTCCTCTACTCCAAAACGCATCGTTACAAGCTTCCTCCGGGACCACCATCACGTCTCTTCGTCGGCCATCTCCACCTCATGAAACCACCGATCCACCGTCTCCTCCAACGCTTCTCCAACCAATACGGCCCAATCTTCTCCCTCCGTTTCGGCTCCCGCCAAGTCGTGATCATCACTTCACCTTCCCTCGCTCAAGAATCCTTCACCGGCCAAAACGACATCATCCTCTCTAGCCGGCCACATCAGCTCACCGCCAAATACGTCGCTTATAATCACACCACCGTCGGAACCGCTCCATACGGTGACCATTGGCGTAACCTCCGCCGAATTTGCTCCCAAGAGATTCTCTCCTCTAACCGTCTCATCAATTTCCAACACATCCGCAAAGACGAGATCTTACGGATGATCACGCGCCTCTCACGTGACACACAAACCTCCAACGGAACCAGAAGTTTCACTCACATCGAGCTCGAACCTCTCTTATCCGATTTAACGTTCAACAACATAGTAAGGATGGTCACAGGGAAGAGATATTACGGCGACGACGTCAACAACAAGGAAGAAGCAGAGCTTTTCAAGAAGCTCGTTTATGATATCGCCATGTATAGTGGCGCGAATCATTCCGCTGATTACTTGCCGGTGCTGAAACTATTCGGAAACAAATTCGAGAAACAGGTTAAAGCTCTGGGCAAAACCATGGATGATATTTTGCAACGTTTGCTTGATGAGTGTAGAAGAGATAAAGAAGGTAACACAATGGTGAATCATTTGCTTTCTCTACAACAACAAGAACCTGAGTATTACACTGACGTTATCATCAAAGGCCTCATGATGTCGATGATGCTCGCCGGGACTGAGACCTCCGCCGTGACTCTAGAGTGGGCGATGGCGAATTTGTTGAGAAATCCAGAAGTATTGGAGAAGGCGAGATCGGAGATCGATGAGAAGATCGGAAAAGATCGTCTGATCGATGAATCAGACATCGCCGTCCTTCCTTATCTCCAAAACGTCGTCTCCGAAACATTCCGGCTATTTCCTGTGGCGCCGTTTTTAATCCCTCGGACGACGACGGAGGATATGAAAATCGGCGGTTACGATGTCCCGCGCGACGCGATAGTGTTGGTTAACGCTTGGGCTATACACAGAGATCCAGAGCTGTGGGAAGATGCAGAGAAGTTTAATCCGGATCGGTTTATCGACGGATGTGTAAATGAGTACAACGTTTACAAGCTGATGCCGTTTGGAAACGGCCGGAGAACTTGTCCCGGCGCCGGATTAGGGCAGAGGATCGTGACGTTGGCGTTGGGATCGTTGATTCAGTGCTTTGATTGGGAGAATGTGAAAGGCGAAGAGATGGATATGTCAGAGAGTGCTGGGTTAGGTATGCGTAAGATGGATCCTTTACGGGCCATGTGCAGGCCTAGGCCCATTATTGCTAAGCTTCTAATC

**APK1**

>CL11525.Contig1_All 81 899 minus strand Adenylyl-sulfate kinase 1 [Arabidopsis thaliana] >gi|7387811|sp|Q43295.1|KAP1_ARATH RecName: Full=Adenylyl-sulfate kinase 1, chloroplastic; AltName: Full=ATP adenosine-5'-phosphosulfate 3'-phosphotransferase; AltName: Full=Adenosine-5'-phosphosulfate kinase; Short=APS kinase; Flags: Precursor >gi|414737|emb|CAA53426.1| APS kinase [Arabidopsis thaliana] >gi|450235|gb|AAC50035.1| APS kinase [Arabidopsis thaliana] >gi|1575322|gb|AAC50034.1| APS kinase [Arabidopsis thaliana] >gi|15810038|gb|AAL06946.1| At2g14750/F26C24.11 [Arabidopsis thaliana] >gi|22135773|gb|AAM91043.1| At2g14750/F26C24.11 [Arabidopsis thaliana] >gi|330251236|gb|AEC06330.1| Adenylyl-sulfate kinase 1 [Arabidopsis thaliana]

ATGATCGCCGCCGGAGCTAAATCTCTTCTAGGGCTTTCAATTGCTTCTCCCAAAGGTATT

TCTGATTGTAATCCGAGATCTGTCGGTGTTGTTCGTTGTTGTGTTTCCATGGAAGGATCT

CAAACAATGAGTCATAACACAAATGGTTCTATTCCTGAGCTCAAATCGATTAATGGTCAC

ACAGGGCAAAAACAAGGTCCATTGTCTACAGTTGGAAACTCAACAAACATAAAATGGCAT

GAATGTTCTGTTGAGAAGGTTGATAGACAAAGATTGCTTGATCAGAAAGGATGTGTAATT

TGGGTCACTGGTCTTAGTGGTTCAGGGAAGAGTACTGTGGCTTGTGCTTTGAATCAGATG

TTGTATCAAAAGGGGAAGCTTTGTTATATTCTTGATGGTGATAATGTTAGGCATGGCTTA

AACCGTGATCTTAGCTTTAAAGCTGAAGATCGTGCTGAGAACATTCGTAGAGTTGGTGAG

GTAGCTAAGCTATTTGCAGATGCTGGAATTATCTGCATTGCAAGTTTAATATCTCCTTAT

AGAAGAGATAGGGACGCTTGTCGAAGTTTGCTTCCCGAGGGAGATTTTGTTGAGGTTTTC

ATGGATGTACCACTTGAAGTTTGCGAGGCGAGAGATCCAAAGGGTCTCTACAAGCTTGCT

CGTGCTGGAAAGATCAAAGGTTTTACCGGTATTGATGACCCTTACGAGCCACCATTGAAT

TGCGAGATTGCTCTGGGACACCAAGAAACAGGAACTTCACCAATAGAAATGGCTGCAACA

GTTGTTGGATACTTAGAAAAGAAGGGTTATCTTCAGGCA

> T_Unigene_BMK.9883 gi|297831946|ref|XP_002883855.1| 3.56916e-179 gi|297831946|ref|XP_002883855.1| hypothetical protein ARALYDRAFT_480365 [Arabidopsis lyrata subsp. lyrata]

TTAATTAAAAAACATTCAGACACAACAAAAACTAGATTCTTTTCTTTTTTTTCCTTGTTCTGTCTTAATCCAAATTCCGATTTCAATTCCATTTCCCACCACACCGTAATTATGATCGCCGCCGGAGCTAAATCTCTTCTAGGGCTTTCAATTGCTTCTCCTAAAGGTGTTTCTGATTGTAATCCGAGATCTGTCGGTGTTGTTCGTTGTTGTGTTTCCATGGAAGGATCTCAAACAATGAGTCATAACAAAAATGGTTCTATTCCTGAGCTCAAATCTATTAATGGTCACACAGGGCAAAAACAAGGTCCATTGTCTACAGTTGGAAACTCAACAAACATAAAATGGCATGAATGTTCTGTTGAGAAGGTTGATAGACAAAGATTGCTTGATCAGAAAGGATGTGTAATTTGGGTCACTGGTCTTAGTGGTTCAGGGAAGAGTACTGTGGCTTGTGCTTTGAATCAGATGTTGTATCAAAAGGGGAAGCTTTGTTATATTCTTGATGGTGATAATGTTAGGCATGGCTTAAACCGCGATCTTAGCTTTAAAGCTGAAGATCGTGCTGAGAACATTCGTAGAGTTGGTGAGGTAGCTAAGCTATTTGCAGATGCTGGAATTATCTGCATTGCAAGTTTAATATCTCCTTATAGAAGAGATAGGGACGCTTGTCGAAGTTTGCTCCCCGAGGGAGATTTTGTTGAGGTTTTTATGGATGTACCACTTGAAGTTTGCGAGGCGAGAGATCCAAAGGGCCTCTACAAGCTTGCTCGTGCGGGAAAGATCAAAGGTTTTACCGGTATTGATGACCCTTACGAGCCACCATTGAATTGCGAGATTGCTCTGGGACACCAAGAAACAGGAACTTCACCAATAGAAATGGCTGCAACAGTTGTTGGATACTTAGAAAAGAAGGGTTATCTTCAGGCA

**APK2**

>CL4873.Contig1_All 372 980 Adenylyl-sulfate kinase 2 [Arabidopsis thaliana] >gi|7387808|sp|O49196.1|KAP2_ARATH RecName: Full=Adenylyl-sulfate kinase 2, chloroplastic; AltName: Full=ATP adenosine-5'-phosphosulfate 3'-phosphotransferase; AltName: Full=Adenosine-5'-phosphosulfate kinase; Short=APS kinase; Flags: Precursor >gi|18087563|gb|AAL58913.1|AF462823_1 AT4g39940/T5J17_110 [Arabidopsis thaliana] >gi|2829133|gb|AAC39520.1| adenosine-5'-phosphosulfate-kinase [Arabidopsis thaliana] >gi|4490745|emb|CAB38907.1| adenosine-5'-phosphosulfate-kinase [Arabidopsis thaliana] >gi|7271049|emb|CAB80657.1| adenosine-5'-phosphosulfate-kinase [Arabidopsis thaliana] >gi|20453397|gb|AAM19937.1| AT4g39940/T5J17_110 [Arabidopsis thaliana] >gi|332661742|gb|AEE87142.1| Adenylyl-sulfate kinase 2 [Arabidopsis thaliana]

TCTTTTTTAATTAATAATTTATTTATTTGTACGTTGATTTTTGTATAATT

TCAGAAAACCGTAACACCCAACACCCGGTTGGGAATTTCTGTGTTATCTT

GGCCGTCGGATTCAATGGAAGGATTAACTATCAGAGCATCGCGACCGTCG

ATTTTCTGTTCTCTTCCAGGTCTCGGCGGCGATTCTCAGCGACGACCTCC

AACTGACGGTTTCCTCAAGCTTCCGGCGTCGTCAAATGCAGTAGATAACA

CAAAATTAGTCACGAACTCTGCTTCTTTTCATCCAATCTCCGCCGTTAAC

GTCTCTGCTCAAGCTTCCCTCACCGCCGATTTTCCCGCCATCTCAGAAAC

GAAGGAAAGGTTCAAAGGAGAAGAGAAGGCAGAGAACATAGTGTGGCACG

AGAGCTCAATCTGCAGATGCGACAGACAACAACTTCTTCAACAAAAGGGT

TGTGTCATTTGGATCACTGGTCTCAGCGGTTCAGGGAAAAGCACTGTTGC

GTTGCTCTAAGTAAAGCATTGTTTGAAAGAGGCAAACTCACTTATACACT

CGACGGCGACAATGTTCGTCACGGCCTTAACCGGGACCTCACTTTCAAAG

CAGAGGACCGTACCGAAAACATACGAAGAATCGGTGAGGTGGCTAAGTTG

TTTGCTGACGTGGGGGTCATTTGTATAGCAAGTTTGATTTCTCCGTACCG

GAGAGACAGAGACGCGTGCCGGTCTTTGTTACCTGAGGGCGATTTCGTCG

AGGTGTTCATGGACGTTCCTCTTCATGTGTGCGAGTCAAGAGATCCCAAG

GGGTTGTACAAGCTTGCACGTGCTGGCAAAATCAAAGGCTTCACCGGAAT

CGACGACCCTTACGAGGCGCCACTGAATTGCGAGGTTGTGCTGAAACACA

CAGGAGACGATGTTTCGTGTTCGCCACGTCAGATGGCTGAGAACATCATC

TCTTACCTGCAAAACAAAGGTTACCTTGAGAGCTAAGCTGAGTCTGAAGA

ATCAAATACAAAAACCCAATTTGTCGAATCCTACTAGTTAGAATGTTTTA

TATATATATAATGTTATTGCTCTCGCTGGTTCGACCAGAAGCTTGAGACC

TCATACGACGTCGTCTCGCTTCGTCACACTATCATTTGTTCTTGAATTGT

GTTAAACTCCGAAGCCAGGTGAAGGGAAAATATTTATATCTTCTTTCTAC

GTATCATTTAGTTAGTTAATAATGAGCTTTGCTCCAATGATATCGACTGT

>T_Unigene_BMK.10173 gi|15236087|ref|NP_195704.1| 0 gi|15236087|ref|NP_195704.1| AKN2 (APS-kinase 2); ATP binding / adenylylsulfate kinase/ kinase/ transferase, transferring phosphorus-containing groups [Arabidopsis thaliana]

CCGTCGGATTCAATGGAAGGATTAACTATCAGAGCATCGCGACCGTCGATTTTCTGTTCTCTTCCAGGTCTCGGCGGCGATTCTCAGCGACGACCTCCAACTGACGGTTTCCTCAAGCTTCCGGCGTCGTCAAATGCAGTAGATAACACAAAATTAGTCACGAACTCTGCTTCTTTTCATCCAATCTCCGCCGTTAACGTCTCTGCTCAAGCTTCCCTCACCGCCGATTTTCCCGCCATCTCAGAAACGAAGGAAATGTTCAAAGGAGAAGAGAAGGCAGAGAACATAGTGTGGCACGAGAGCTCAATCTGCAGATGCGACAGACAACAACTTCTTCAACAAAAGGGTTGTGTCATTTGGATCACTGGTCTCAGCGGTTCAGGGAAAAGCACTGTTGCGTGTGCTCTAAGTAAAGCATTGTTTGAAAGAGGCAAACTCACTTATACACTCGACGGCGACAATGTTCGTCACGGCCTTAACCGGGACCTCACTTTCAAAGCAGAGGACCGTACCGAAAACATACGAAGAATCGGTGAGGTGGCTAAGTTGTTTGCTGACGTGGGGGTCATTTGTATAGCAAGTTTGATTTCTCCGTACCGGAGAGACAGAGACGCGTGCCGGTCTTTGTTACCTGAGGGCGATTTCGTCGAGGTGTTCATGGACGTTCCTCTTCATGTGTGCGAGTCAAGAGATCCCAAGGGGTTGTACAAGCTCGCACGTGCTGGCAAAATCAAAGGCTTCACCGGAATCGACGACCCTTACGAGGCGCCAATGAATTGCGAGGTTGTGCTGAAACACACAGGAGACGACGTTTCGTGTTCGCCACGTCAGATGGCTGAGAACATCATCTCTTACCTGCAAAACAAAGGTTACCTTGAGAGC

**GSH1**

>CL51.Contig13_All 157 1719 minus strand gamma-glutamylcysteine synthetase [Chorispora bungeana]

ATGGCGCTCTTGTCTCAGGCGGGAGGATCATACACTGTCCCTTCTGGACCTGTATGCTCA

AAGAATGGAACTAAAGCAGTTTCTGGTGGTGTGAGAAATTTGGATGTGTTGAGGATGAAA

GATGCCTATGTTAGCTCCTACTCTAGGAGTGTATCTACCAAATCAATGCTCCTCCGTTCT

GTTAAGAGGAGCAAGAGAGGGGATCAATTGATTGTTGCGGCAAGCCCTCCAACAGAAGAG

GCTGTAGTTGCGACTGAGCCACTTACGAGAGAGGATCTCATCGCTTATCTTGCCTCTGGA

TGCAAATCAAAGGAAAAATGGAGAATAGGTACAGAGCATGAGAAATTTGGTTTTGAGGTC

AATAGTTTGCGCCCTATGAAGTATGATCAAATAGCCGAGCTGCTTAATAGTATCGCTGAA

AGATTTGAATGGGAAAAAGTAATGGAAGGTGACAAGATCATTGGTCTGAAGCAGGGAAAG

CAAAGCATTTCACTGGAACCTGGGGGTCAATTCGAGCTTAGTGGTGCACCTCTTGAGACT

TTGCACCAAACTTGTGCTGAAGTCAATTCGCACCTTTATCAGGTAAAAGCTGTTGCTGAG

GAAATGGGAATTGGTTTCTTAGGAATCGGCTTCCAGCCCAAATGGCGCCGGGAGGATATA

CCCATCATGCCAAAGGGGAGATATGATATTATGAGAAACTACATGCCGAAAGTGGGTTCC

CTTGGACTTGATATGATGCTTCGAACATGTACTGTTCAGGTTAATCTGGATTTTAGCTCA

GAAGCTGATATGATCAGGAAGTTCCGCGCTGGTCTTGCTTTGCAACCTATAGCAACGGCT

CTATTTGCGAATTCCCCCTTTACCGAAGGAAAGCCGAATGGGTTTCTCAGCATGAGAAGC

CATATATGGACAGACACTGACAAGGACCGCACAGGAATGCTACCGTTTGTTTTCGACGAC

TCTTTTGGGTTTGAGCAGTATGTTGACTACGCACTCGATGTCCCTATGTACTTTGCCTAC

CGAAACAAGAAATACGTCGACTGTACTGGAATGACATTTCGGCAATTTTTGGCTGGAAAG

CTTCCTTGTCTCCCTGGTGAACTGCCTACGTATAATGATTGGGAAAATCATCTAACAACA

ATATTCCCAGAGGTTCGGTTGAAGAGATACTTGGAGATGAGAGGTGCTGATGGAGGTCCT

TGGAGGAGGTTGTGTGCCCTGCCAGCTTTCTGGGTGGGTTTACTATATGACGAGGATACA

CTCCAAGCTATCCTGGATCTGACAGCTGACTGGACTCCAGCAGAAAGAGAGATGCTAAGG

AACAAAGTTCCAGTAACTGGCTTGAAGACACCGTTTAGAGATGGTTTGTTAAAGCATGTC

GCTGAAGATGTCCTGAAACTCGCAAAGGATGGTTTAGAGCGTAGAGGCTACAAGGAAGCA

GGTTTCTTAAATGCAGTCGCTGAAGTGGTCAGAACAGGAGTTACACCAGCGGAGAAGCTC

TTGGAATTGTACAATGGAGAGTGGGGACAAAGCGTAGACCCAGTGTTCGAAGAACTCCTG

TAC

> T_Unigene_BMK.5893 gi|312282405|dbj|BAJ34068.1| 0 gi|312282405|dbj|BAJ34068.1| unnamed protein product [Thellungiella halophila]

CGAATTCAGTTGCAGGAGCTATATACGATGGCGCTCTTGTCTCAGGCGGGAGGATCATACACTGTCCCTTCTGGACCTGTATGCTCAAAGAATGGAACTAAAGCAGTTTCTGGTGGTGTGAGAAATTTGGATGTGTTGAGGATGAAAGATGCCTATGTTAGCTCCTACTCTAGGAGTCTATCTACCAAATCAATGCTCCTCCGTTCTGTTAAGAGGAGCAAGAGAGGGGATCAATTGATTGTTGCGGCAAGCCCTCCAACGGAAGAGGCTGTAGTTGCGACTGAGCCACTTACGAGAGAGGATCTCATCGCTTATCTTGCCTCTGGATGCAAATCAAAGGAAAAATGGAGAATAGGTACAGAGCATGAGAAATTTGGTTTTGAGGTCAATAGTTTGCGCCCTATGAAGTATGATCAAATAGCCGAGCTGCTTAATAGTATCGCTGAAAGATTTGAATGGGAAAAAGTAATGGAAGGTGACAAGATCATTGGTCTGAAGCAGGGAAAGCAAAGCATTTCACTGGAACCTGGGGGTCAATTCGAGCTTAGTGGTGCACCTCTTGAGACTTTGCACCAAACTTGTGCTGAAGTCAATTCGCACCTTTATCAGGTAAAAGCTGTTGCTGAGGAAATGGGAATTGGTTTCTTAGGAATCGGCTTCCAGCCCAAATGGCGCCGGGAGGATATACCCATCATGCCAAAGGGGAGATATGATATTATGAGAAACTACATGCCGAAAGTGGGTTCCCTTGGACTTGATATGATGCTTCGAACATGTACTGTTCAGGTTAATCTGGATTTTAGCTCCGAAGCTGATATGATCAGGAAGTTCCGCGCTGGTCTTGCTTTGCAACCTATAGCAACGGCTCTATTTGCGAATTCCCCCTTTACCGAAGGAAAGCCGAACGGGTTTCTCAGCATGAGAAGCCATATATGGACAGACACTGACAAGGACCGCACAGGAATGCTACCGTTTGTTTTCGACGACTCTTTTGGGTTTGAGCAGTATGTTGACTACGCACTCGATGTCCCTATGTACTTTGCCTACCGAAACAATAAATACGTCGACTGTACTGGAATGACATTTCGGCAATTTTTGGCTGGAAAACTTCCTTGTCTCCCTGGTGAACTGCCTACGTATAATGATTGGGAAAATCATCTAACAACAATATTCCCAGAGGTTCGGTTGAAGAGATACTTGGAGATGAGAGGTGCTGATGGAGGTCCTTGGAGGAGGTTGTGTGCCCTGCCAGCTTTCTGGGTGGGTTTACTATATGACGAGGATACACTCCAAGCTATCCTGGATCTGACAGCTGACTGGACTCCAGCAGAAAGAGAGATGCTAAGGAACAAAGTTCCAGTAACTGGCTTGAAGACGCCGTTTAGAGATGGTTTGTTAAAGCATGTCGCTGAAGATGTCCTGAAACTCGCAAAGGATGGTTTAGAGCGTAGAGGCTACAAGGAAGCAGGTTTCTTAAATGCAGTCGCTGAAGTGGTCAGAACAGGAGTAACACCAGCGGAGAAGCTCTTGGAATTGTACAATGGAGAGTGGGGACAAAGCGTAGACCCAGTGTTCGAAGAACTCCTGTAC

**MYR**

>CL766.Contig2_All 33 1643 minus strand myrosinase [Armoracia rusticana]

ATGAAGCTTCTTGGACTCGCCTTAGTTCTTCTCTTAGCTGTGGTGACTTGCAAAGCTGAG

ATTACTTGCGAAGACGACAATCCATTCACATGTAGCCAAACTGAACGTTTAAACAGAAAC

CATTTCGATCCGGATTTTGTCTTCGGTGTTGCATCTTCGGCTTACCAGATCGAAGGTAGC

AGAGGTCGTGGAATTAACACTTGGGATGCTTTCACTCACCGATATCCAGAGAAAGGAGGA

CCTGACTTGGGGAATGGAGACACTACTTGTGGATCATATGAGCACTGGCAGAAAGATTTA

GACGTAATGGACGAACTCGGGGTTGACGCCTACAGATTCTCACTTGCGTGGTCAAGAATC

GTTCCGAGAGGAAAGGTGAGTAGGGGAATCAACCAAGACGGTGTTAGGTACTACAACAAT

CTCATAGATGGCCTCCTAGAAAAGAATATAACTCCTTTTGTTACAATCTACCACTGGGAC

CTTCCTCAGTGTCTACAAGATGAGTATGAAGGCTTCTTGGACAGCGAGATCGTAGAGGAT

TTCAAAAATTACGCGGATCTTTGCTTCCAGCTATTTGGTGACAGAGTAAAGAACTGGATT

ACGATCAACCAGCTCTTCACAGTGCCTACGAGAGGCTATGCAACAGGAACAGATGCACCT

GGTCGATGTTCTTCATGGCTTAACAAAGGTTGTTACGCCGGAGATTCTGGAACGGAACCT

TACATCGTTGCACACAACCAGCTTCTTGCTCATGCCACAGCCGTTGATCTTTACAGGAAG

AAATATAAGAAGGAACAAGGAGGACAAATCGGACCTGTGATGATAACAAGATGGTTCCTT

CCATATGATGACACTCAAGAGAGTAAAGATGCAACTGAGAGGAACAAAGAATTCTTCTTG

GGATGGTTCATGGAGCCGCTAACAAAGGGTAAATACCCAGACATCATGAGGAAGCTTGTG

GGTGATAGGCTTCCTGAGTTCACGGAAGCAGAATCCAAACTTGTAAAGGGTTCATTTGAT

TTTCTTGGTCTCAACTATTACTTCACTCAGTTTGTCTACGCCATTCCTCCAAATCCTCCG

AACAGACTCACTGTCATGAATGACTCACTCTCAGCACTCTCATATGTAAATAAGGATGGT

CCCATTGGTCCATGGTTCAATGCAGAATTGTATTACCGCCCAAGAGGTATCTTAGACACA

ATGGAGTACTTCAAAACAAGATACGACAATCCTTCAGTCTATATCACAGAGAATGGATTT

AGTAGCCCCGGTGGTGACACTCCTCAGGAGGTGGTTATTGCTGATGATAACCGGACTGAT

TATCTCTGCAGTCATCTCTGTTTCCTCCGCAAGGCCATCCAGGAGTCGGGTTGTAACGTG

AAAGGATACTTTGTGTGGTCTCTTGGCGATAATTATGAATTCTGCCAAGGCTTTACCGTC

AGATTCGGAGTTAGTTATGTTGATTTCAAGAATATCACTGCTGACAGAGACCTCAAAGAT

TCTGGCAAATGGTATAAGCGGTTCTTGTCCGTAAAGGACAACAAGGTCACTGAGAACCAA

GACCTCCTCCGCTCAAGGTTCTTCATGGGTCCGAAGAAGGTTGCCGATGCA

>CL766.Contig4_All 33 1643 minus strand myrosinase [Armoracia rusticana]

ATGAAGCTTCTTGGACTCGCCTTAGTTCTTCTCTTAGCTGTGGTGACTTGCAAAGCTGAG

ATTACTTGCGAAGACGACAATCCATTCACATGTAGCCAAACTGAACGTTTAAACAGAAAC

CATTTCGATCCGGATTTTGTCTTCGGTGTTGCATCTTCGGCTTACCAGATCGAAGGTAGC

AGAGGTCGTGGAATTAACACTTGGGATGCTTTCACTCACCGATATCCAGAGAAAGGAGGA

CCTGACTTGGGGAATGGAGACACTACTTGTGGATCATATGAGCACTGGCAGAAAGATATA

GACGTAATGGACGAGCTCGGGGTTGACGCCTATAGATTCTCGCTTGCTTGGTCAAGAATC

GTTCCGAGGGGAAAGGTGAGTAGAGGAATCAACCAAGACGGTGTTAGGTACTACAACAAT

CTCATAGATGGTCTCCTAGAAAAGAATATAACTCCTTTCGTTACCATCTACCATTGGGAC

CTTCCTCAATGTCTACAAGATGAGTATGAAGGCTTCTTGGACCGTGAGATCATAGAGGAT

TTCAAAAACTATGCAGATCTTTGCTTCCAGCTATTTGGTGACAGAGTAAAGAACTGGATT

ACGATCAACCAGCTCTTCACAGTGCCTACGAGAGGCTATGCAACAGGAACAGATGCACCT

GGTCGATGTTCTTCATGGCTTAATAAAAATTGCTACGCCGGAGATTCTGGAACAGAACCT

TACATCGTTGCACACAACCAGCTTCTTGCTCATGCCACGGCCGTTGATCTTTACAGGAAG

AAGTATAAGAAGGAACAAGGAGGACAAATCGGACCTGTGATGATAACAAGATGGTTCCTT

CCATATGATAACACTAAAGCCAATATTGATGCAACTGAAAGGAACAAAGAATTCTTCTTG

GGATGGTTCATGGAGCCGCTAACAAAGGGTAAATACCCAGACATCATGAGGAAACTCGTG

GGTGATAGGCTTCCCAAGTTCAATAGTTCAGAATCCAAACTTGTAAAGGGTTCATTTGAT

TTTCTTGGTCTCAACTATTACTTCACTCAGTACGTCTACGCCATTCCCCCGAATCCTCCG

AACAGACTCACCGTCATGAATGATTCACTCTCTGCACTCGCATATGCAAATAAGGATGGC

CCTATTGGTCCATGGTTCAATGCAGATATGTATTACCGCCCAAGAGGCATTTTGGACATA

ATGAAGCACTTCAAAACCAAATACGACAACCCTTTAGTCTATGTCACCGAGAACGGATAT

AGTAGCCCCGGTAGTGACACGCCCTTTGAGGAGGCTATTGCTGATTCCAACCGGACAGAT

TATCTCTGTAGTCATCTCTGTTTTCTCCGCAAGGCTATCAAGGAGTCTGGTTGCAACGTG

AAAGGATACTTTGTATGGTCTCTTGGCGATAATTATGAATTCTGCCAAGGCTTTACCGTC

AGATTCGGAGTTAGTTATGTTGATTTCAAGAATATCACTGCTGACAGAGACCTCAAAGAT

TCTGGCAAATGGTATAAGCGGTTCTTGTCCGTAAAGGACAACAAGGTCACTGAGAACCAA

GACCTCCTCCGCTCAAGGTTCTTCATGGGTCCGAAGAAGGTTGCCGATGCA

>Unigene1232_All 33 1643 minus strand myrosinase [Armoracia rusticana]

ATGAAGCTTCTTGGACTCGCCTTAGTTCTTCTCTTAGCTGTGGTGACTTGCAAAGCTGAG

ATTACTTGCGAAGACGACAATCCATTCACATGTAGCCAAACTGAACGTTTAAACAGAAAC

CATTTCGATCCGGATTTTGTCTTCGGTGTTGCATCTTCGGCTTACCAGATCGAAGGTAGC

AGAGGTCGTGGAATTAACACTTGGGATGCTTTCACTCACCGATATCCAGAGAAAGGAGGA

CCTGACTTGGGGAATGGAGACACTACTTGTGGATCATATGAGCACTGGCAGAAAGATATA

GACGTAATGGACGAGCTCGGGGTTGACGCCTATAGATTCTCGCTTGCTTGGTCAAGAATC

GTTCCGAGGGGAAAGGTGAGTAGAGGAATCAACCAAGACGGTGTTAGGTACTACAACAAT

CTCATAGATGGTCTCCTAGAAAAGAATATAACTCCTTTCGTTACCATCTACCATTGGGAC

CTTCCTCAATGTCTACAAGATGAGTATGAAGGCTTCTTGGACCGTGAGATCATAGAGGAT

TTCAAAAACTATGCAGATCTTTGCTTCCAGCTATTTGGTGACAGAGTAAAGAACTGGATT

ACGATCAACCAGCTCTTCACAGTGCCTACGAGAGGCTATGCAACAGGAACAGATGCACCT

GGTCGATGTTCTTCATGGCTTAATAAAAATTGCTACGCCGGAGATTCTGGAACAGAACCT

TACATCGTTGCACACAACCAGCTTCTTGCTCATGCCACGGCCGTTGATCTTTACAGGAAG

AAGTATAAGAAGGAACAAGGAGGACAAATCGGACCTGTGATGATAACAAGATGGTTCCTT

CCATATGATAACACTAAAGCCAATATTGATGCAACTGAAAGGAACAAAGAATTCTTCTTG

GGATGGTTCATGGAGCCGCTAACAAAGGGTAAATACCCAGACATCATGAGGAAACTCGTG

GGTGATAGGCTTCCCAAGTTCAATAGTTCAGAATCCAAACTTGTAAAGGGTTCATTTGAT

TTTCTTGGTCTCAACTATTACTTCACTCAGTACGTCTACGCCATTCCCCCGAATCCTCCG

AACAGACTCACCGTCATGAATGATTCACTTTCTGCACTCGCATATGCAAATAAGGATGGC

CCTATTGGTCCATGGTTCAATGCAGATATGTATTACCGCCCAAGAGGCATTTTGGACATA

ATGAAGCACTTCAAAACCAAATACGACAACCCTTTAGTCTATGTCACCGAGAACGGATAT

AGTAGCCCCGGTAGTGACACGCCCTTTGAGGAGGCTATTGCTGATTCCAACCGGACAGAT

TATCTCTGTAGTCATCTCTGTTTTCTCCGCAAGGCTATCAAGGAGTCTGGTTGCAACGTG

AAAGGATACTTTGTATGGTCTCTTGGCGATAATTATGAATTCTGCCAAGGCTTTACCGTT

AGATTCGGAGTTAGTTACGTTGATTTCAAGAATATCACTGCCGACAGAGACCTCAAAAGA

TCTGGCCAATGGTACCAGCGGTTCTTGTCCGTAAAGGACAACAAGGTCCGTGAAAACCAA

GATGTCCTCCGCTCAAGGTTCTTCTTGGGTCAGAAGAAGGTTGCCGATGCA

>CL766.Contig1_All 33 1643 minus strand myrosinase [Armoracia rusticana]

ATGAAGCTTCTTGGACTCGCCTTAGTTCTTCTCTTAGCTGTGGTGACTTGCCAAGCTGAG

ATCACTTGCGAAGATGAAAAGCCTTTCACATGTAACCAAACTGAACGTTTAAACAGAAAC

CATTTCGATCCGGACTTTATCTTCGGTGTTGCATCTTCTGCTTACCAGATCGAAGGTAGC

AAAGGTCGTGGAGTTAACACTTGGGATGCCTTCACTCACCGATATCCAGAGAAAGGAGGA

GAGGATTTGGGGAATGGAGATACTGCTTGTGGATCATATGAGCACTGGCAGAAAGATTTA

GACGTAATGGACGAACTCGGGGTTGACGCCTACAGATTCTCACTTGCGTGGTCAAGAATC

GTTCCGAGAGGAAAGGTGAGTAGGGGAATCAACCAAGACGGTGTTAGGTACTACAACAAT

CTCATAGATGGCCTCCTAGAAAAGAATATAACTCCTTTTGTTACAATCTACCACTGGGAC

CTTCCTCAGTGTCTACAAGATGAGTATGAAGGCTTCTTGGACAGCGAGATCGTAGAGGAT

TTCAAAAATTACGCGGATCTTTGCTTCCAGCTATTTGGTGACAGAGTAAAGAACTGGATT

ACGATCAACCAGCTCTTCACAGTGCCTACGAGAGGCTATGCAACAGGAACAGATGCACCT

GGTCGATGTTCTTCATGGCTTAATAAAAATTGCTACGCCGGAGATTCTGGAACAGAACCT

TACATCGTTGCACACAACCAGCTTCTTGCTCATGCCACGGCCGTTGATCTTTACAGGAAG

AAGTATAAGAAGGAACAAGGAGGACAAATCGGACCAGTGATGATAACAAGATGGTTCCTT

CCATATGATAACACTAAAGCCAATATTGATGCAACTGAAAGGAACAAAGAATTCTTCTTG

GGATGGTTCATGGAGCCGCTAACAAAGGGTAAATACCCAGACATCATGAGGAAGCTTGTG

GGTGATAGGCTTCCTGAGTTCACGGAAGCAGAATCCAAACTTGTAAAGGGTTCCTTTGAT

TTTCTTGGTCTCAACTATTACTTCACTCAGTTTGTCTACGCCATTCCTCCAAATCCTCCG

AACAGACTCACTGTCATGAATGACTCACTCTCAGCACTCTCATATGTAAATAAGGATGGT

CCCATTGGTCCATGGTTCAATGCAGAATTGTATTACCGCCCAAGAGGTATCTTAGACACA

ATGGAGTACTTCAAAACAAGATACGACAATCCTTCAGTCTATATCACAGAGAATGGATTT

AGTAGCCCCGGTGGTGACACTCCTCAGGAGGTGGTTATTGCTGATGATAACCGGACTGAT

TATCTCTGCAGTCATCTCTGTTTCCTCCGCAAGGCCATCCAGGAGTCGGGTTGTAACGTG

AAAGGATACTTTGTGTGGTCTCTTGGCGATAATTATGAATTCTGCCAAGGCTTTACCGTT

AGATTCGGAGTTAGTTACGTTGATTTCAAGAATATCACTGCCGACAGAGACCTCAAAAGA

TCTGGCCAATGGTACCAGCGGTTCTTGTCCGTAAAGGACAACAAGGTCCGTGAAAACCAA

GATGTCCTCCGCTCAAGGTTCTTCTTGGGTCAGAAGAAGGTTGCCGATGCA

>CL766.Contig5_All 1 900 minus strand myrosinase [Armoracia rusticana]

GAACCTTACATCGTTGCACACAACCAGCTTCTTGCTCATGCCACTGTCGTCGATCTTTAC

AGGAAAAAGTACAAGAGTGAACAAGGAGGACAGATTGGACCCGTGATGATAACTAGATGG

TTTCTTCCATTTGATAACACTACAGCCAACCTAGATGCAACCGAGAGGAACAAAGAATTC

TTCTTGGGATGGTTCATGGAGCCGCTAACAAAGGGTAAATATCCAGACATCATGAGGCAA

CTTGTGGGTGATAGGCTTCCCGAATTCACGAAATCAGAATCTGAACTTGTAAAGGGTTCA

TACGATTTTCTTGGTCTCAACTATTACTTCACTCAGTACGTCTACGCCATTCCTCCAAAT

GCTCCGGATAGACTCACCGTCATGAATGACTCACTCTCAGCACTCGCATATGAAAACAAG

GACGGCCCCATTGGTCCATGGTTTAACAATGAAGGCGTATATTACCGTCCACAAGGCATT

CAAGACATAATGGAGCACTTCAAAACTAAATACGAAAATCCGTTAGTCTATGTCACTGAG

AACGGATTTAGTAGCCCCGGTGGTGACACACAACATACAGAGTGTTTGGCTGATTCCAAC

CGGACTGATTATCTCTGTAGTCATCTCTGTTTTCTCCGCAAGGCTATCAAGGAGTCTGGT

TGCAACGTGAAAGGATACTTTGTATGGTCTCTTGGCGATAATTATGAATTCTGCCAAGGC

TTTACCGTCAGATTCGGAGTTAGTTATGTTGATTTCAAGAATATCACTGCTGACAGAGAC

CTCAAAGATTCTGGCAAATGGTATAAGCGGTTCTTGTCCGTAAAGGACAACAAGGTCACT

GAGAACCAAGACCTCCTCCGCTCAAGGTTCTTCATGGGTCCGAAGAAGGTTGCCGATGCA

> T_Unigene_BMK.16179 gi|56112345|gb|AAV71147.1| 0 gi|56112345|gb|AAV71147.1| myrosinase [Armoracia rusticana]

GGGATGCTTTCACTCACAGATGAACACAAAACATACATCTATCAATTAACCATGAAGCTCCTTGGAACCGCCTTAGTTCTTTTATTAGCTGTGGTGACTTGCAAAGCTGAAGAGATTACTTGCGAAGAGAATTTACCATTCACATGTAACAAAACTGATCGTTTCAATAGAAGCCATTTCGACGACGACTTCATCTTCGGTGTTGCATCTTCTGCTTACCAGATTGAAGGTGGCGAAGATCGTGGAGTTAACACTTGGGATGCCTTCACTCACCGATATCCAGAGAAAGGAGGAGAGGATTTGGGGAATGGAGATACTGCTTGTGGATCATATGAGCACTGGCAGAAAGATATAGACGTAATGGACGAACTCGGGGTTGACGCCTACAGATTCTCACTTGCGTGGTCAAGAATCGTTCCGAGAGGAAAGGTGAGTAGGGGAATCAACCAAGACGGTGTTGCGTACTACAACAATCTCATAGATGGCCTCCTAGAAAAGAATATCACTCCTTTTGTTACAATCTACCACTGGGACCTTCCTCAGTGTCTACAAGATGAGTATGAAGGCTTCTTGGACAGCGAGATCGTAGAGGATTTCAAAAATTACGCGGATCTCTGCTTCCAGCTATTTGGTGACAGAGTAAAGAACTGGATTACGATCAACCAACTCTTCACAGTGCCTACGAGAGGCTATGCAACAGGAACAGATGCACCTGGTCGATGTTCTTCATGGCTTAACAAAGGTTGTTACGCCGGAGATTCTGGAACGGAACCTTACATCGTTGCACACAACCAGCTTCTTGCTCATGCCACAGCCGTTGATCTTTACAGGAAGAAATATAAGAAGGAACAAGGAGGACAAATCGGACCAGTGATGATAACAAGATGGTTCCTTCCATATGATGACACTCAAGAGAGTAAAGATGCAACTGAGAGGAACAAAGAATTCTTCTTGGGATGGTTCATGGAGCCGCTAACAAAGGGTAAATACCCAGACATCATGAGGAAGCTTGTGGGTGAAAGGCTTCCCGAGTTCACGGAAGCAGAATCCAAACTTGTAAAGGGTTCCTTTGATTTTCTTGGTCTCAACTATTACTTCACTCAGTACGTCTACGCCATTCCTCCAAATGCTCCGGATAGACTCACCGCCATGAGTGACTCACAATGTGACTACAAATATGTAAATTTCACTGGAAAGCCCAGTGGTCCACCAGCCGGTGAAGGCAGATATTACTATCCAAGGGGCATTTTACAAGTAATGGATTACTTCAAAACCAATTATAGCAACCCTTTAATCTATGTCACTGAAAACGGAATTAGTACCCCCGGTGGTAATTTAACCAAAAATGAGACTATGGCTGATCCCGAACGTACTGATTATCTCTGCAGTCATCTCTGTTTCCTCCGCAAGGCCATCCAGGAGTCGGGTTGTAACGTGAAAGGATACTTTGTGTGGTCTCTTGGCGATAATTATGAATTCTGCCAAGGCTTTACCGTTAGATTCGGAGTTAGTTACGTTGATTTCAAGAATATCACTGCCGACAGAGACCTCAAAAGATCTGGCCAATGGTACCAGCGGTTCTTGTCCGTAAAGGACAACAAGGTCCGTGAAAACCAAGATGTCCTCCGCTCAAGGTTCTTCTTGGGTCAGAAGAAGGTTGCCGATGCA

**Dof1.1**

>CL441.Contig6_All 91 1059 Dof zinc finger protein DOF1.1 [Arabidopsis thaliana] >gi|8439908|gb|AAF75094.1|AC007583_30 Strong similarity to zinc finger protein OBP2 from Arabidopsis thaliana gb|AF155816. EST gb|N65215 comes from this gene >gi|332190033|gb|AEE28154.1| Dof zinc finger protein DOF1.1 [Arabidopsis thaliana]

CAGCCTACAAATTCGAATCATCAGCATCACCAGCTTCACGAAAATGGAAGTCTAGTTAGT

GGCCATGGACTACTCTCTCATCAACTTCCACCTCTCCAAGCAAACCCTAACCCTAACCCT

AACCACCACCATGCCACTGCCTCTGCTGGACTTCCGGCGAGGATGGGTGGATCGATGGTG

GAGAGAGCGAGACAGGCCAAACTTGCTCCGCCTGAGGGACCCATAAAGTGTCCTCGATGC

GACTCCATCAACACTAAGTTCTGTTACTACAACAACTATAACCTCTCTCAGCCTCGTCAC

TTCTGCAAAGGTTGCCGTCGCTACTGGACACAAGGTGGCGCCCTAAGGAACGTCCCTGTC

GGTGGAGGCTGCCGGAGGAATAACAAGAAGGGCAAAAATGGAAATTCAAAATCTTCTTCT

TCTTCGTCAAAACAGTCTTCCACGGTCAACGCTTCAAGTCCTAGCTCAGGACAGCTAAGG

ACAAATCATCAGTTCCCATTTTCACCAACTCTTTACAATCTCACTCAACTCGGAGGTATA

GGTTTGAACTTAGCCGCCACTAATGGCAACAACCAAGCTCACCAGATCGGTTCCAGTTTG

ATGAACGATCTAGGGTTTCTCCATGTCGGAAATGGACGAAATACTTCAACTCTGATTACC

GGAAACATTCATGACAACAACAACAACAATGAAAGCAACCTAATGAGTCACTTCGCTCTC

TTCGATCCAACGACGGGGCTATACGCTTTCCCGAACGAGGGTAATATCGGAAACAACGTC

GGGATATCTTGTTCCCCTGCTTCCATGGTCGATTCTAGGGCTTACCAGACAGCTCCGGTG

AAGATGGAAGAGCAGCCTAATTCGGTTAACTTGCCTAGACCGATCTCCGGTTTGACGTCT

CCGGGGAATCAAACTAATCAGTACTACTGGAGCGGTTCGGATTTCTCCGGTCCTTCTAAT

GAACTCTTG

>CL441.Contig1_All 88 819 minus strand Dof zinc finger protein DOF1.1 [Arabidopsis thaliana] >gi|8439908|gb|AAF75094.1|AC007583_30 Strong similarity to zinc finger protein OBP2 from Arabidopsis thaliana gb|AF155816. EST gb|N65215 comes from this gene >gi|332190033|gb|AEE28154.1| Dof zinc finger protein DOF1.1 [Arabidopsis thaliana]

TCGAATAATCATCCTCATCATCTTCAGATTCAAGAAAGTGGAAGTTTAGTTAATGGTCAC

AACCAAGTACCCTCTCACCACTTCCCACAAAACCCTAACCCTAACCACCACCATGCCGCT

GTCTCTGCTGGACTTCCGGCGAGGATGGGTGGATCGATGGTGGAGAGAGCGAGACAGGCC

AAACTTGCTCCGCCTGAGGGACCCATAAAGTGTCCTCGATGCGACTCCATCAACACTAAG

TTCTGTTACTACAACAACTATAACCTCTCTCAGCCTCGTCACTTCTGCAAAGGTTGCCGT

CGCTACTGGACACAAGGTGGCGCCCTGAGGAACGTCCCTGTCGGTGGAGGCTGCCGGAGG

AATAACAAGAAGGGCAAAAATGGAAATTCAAAATCTTCTTCTTCTTCGTCAAAACAGTCT

TCCACGGTCAACGCTTCAAGTCCTAGCTCAGGACAGCTAAGGACAAATCATCAGTTCCCA

TTTTCACCAACTCTTTACAATCTCACTCAACTCGGAGGTATAGGTTTGAACTTAGCCGCC

ACTAATGGCAACAACCAAGCTCACCAGATCGGTTCCAGTTTGATGAACGATCTAGGGTTT

CTCCATGTCGGAAATGGACGAAATACTTCGACTCTGATTACCGGAAACATTCATGACAAC

AACAACAACAATGAAAGCAACCTAATGAGTCACTTCGCTCTCTTCGACCCAACGACAGGG

CTATACGCTTTC

> T_Unigene_BMK.20715 gi|297849058|ref|XP_002892410.1| 0 gi|297849058|ref|XP_002892410.1| predicted protein [Arabidopsis lyrata subsp. lyrata]

GGTTTCTTGGTCGTTCCTTCTTTTAATTTGATTACTTTGATTTATTGTTTGGTTTTGCAGCCTACAAATTCAAATCATCAGCATCACCAGCTTCACGAAAATGGAAGTCTAGTTAGTGGCCATGGACTACTCTCTCATCAACTTCCACCTCTCCAAGCAAACCCTAACCCTAACCCTAACCACCACCATGCCACTGCCTCTGCTGGACTTCCGACGAGGATGGGTGGATCGATGGTGGAGAGAGCGAGACAGGCCAAACTTGCTCCGCCTGAGGGACCCATAAAGTGTCCTCGATGCGACTCCATCAACACTAAGTTCTGTTACTACAACAACTATAACCTCTCTCAGCCTCGTCACTTCTGCAAAGGTTGCCGTCGCTACTGGACACAAGGTGGCGCCCTGAGGAACGTCCCTGTCGGTGGAGGCTGCCGGAGGAATAACAAGAAGGGCAAAAATGGAAATTCAAAATCTTCTTCTTCTTCGTCAAAACAGTCTTCCACGGTCAACGCTTCAAGTCCTAGCTCAGGACAGCTAAGGACAAATCATCAGTTCCCATTTTCACCAACTCTTTACAATCTCACTCAACTCGGAGGTATAGGTTTGAACTTAGCCGCCACTAATGGCAACAACCAAGCTCACCAGATCGGTTCCAGTTTGATGAACGATCTAGGGTTTCTCCATGTCGGAAATGGACGAAATACTTCAACTCCGATTACCGGAAACATTCATGACAACAACAACAATGAAAGCAACCTAATGAGTCACTTCGCTCTCTTCGACCCAACGACGGGGCTATACGCTTTCCCGAACGAGGGTATTATCGGAAACAACGTCGGGATATCTTGTTCCCCTGCTTCCATGGTCGATTCTAGGGCTTACCAGACAGCTCCGGTGAAGATGGAAGAGCAGCCTAATTCGGTTAACTTGCCTAGACCGATCTCCGGTTTGACGTCTCCGGGGAATCAAACTAATCAGTACTACTGGACCGGTTCGGATTTCTCCGGTCCTTCTAATGAACTCTTG

**IQD1-1**

>CL10699.Contig1_All 273 1631 minus strand protein IQ-domain 2 [Arabidopsis thaliana] >gi|238481199|ref|NP_001154693.1| protein IQ-domain 2 [Arabidopsis thaliana] >gi|334187391|ref|NP_001190211.1| protein IQ-domain 2 [Arabidopsis thaliana] >gi|15982840|gb|AAL09767.1| AT5g03040/F15A17_70 [Arabidopsis thaliana] >gi|23506103|gb|AAN28911.1| At5g03040/F15A17_70 [Arabidopsis thaliana] >gi|332003165|gb|AED90548.1| protein IQ-domain 2 [Arabidopsis thaliana] >gi|332003166|gb|AED90549.1| protein IQ-domain 2 [Arabidopsis thaliana] >gi|332003167|gb|AED90550.1| protein IQ-domain 2 [Arabidopsis thaliana]

ATGGGCAAAAAAGGAAAATGGTTTTCAAGTGTTAAGAAAGCTTTCAGCCCAGATTCAAAG

AAGTCGAAACAAAAATTGGCTGAGAGCCAAAATGGTGTGATCTCTAATCCTCCTTTGCCG

GATAATGCCAGACAAGCTTCTCCTCCTGCTGCTCCTCCTCCTCTTGAGGTGAGAGTAGCT

GAAGTGATTGTTGAACAGAACAGGAATATTTCTCCTCCTTCCACGGCAGATGTTGTGAAT

GTTACAGCCACTGATGTACCTGTAGTTCCATCTTCATCTACTCCTGAGGTTGTTCGTCGT

GCTACAGCTACTCGATTTGCTGGAAAGTCAAACGAAGAAGCGGCTGCAATCTTGATTCAG

ACTATCTTTAGAGGCTACTTGGCAAGAAGAGCACTGAGGGCAATGAGGGGTTTGGTCAGA

CTTAAGTTATTGATGGAAGGATCTGTTGTTAAACGGCAAGCTGCAAATACTCTAAAATGT

ATGCAGACTCTCTCTCGGGTACAGTCACAGATCCGAGCTAGGAGAATCAGGATGTCAGAA

GAGAATCAAGCTCGCCAGAAGCAACTCCTTCAGAAGCATGCCAAAGAGTTAGCTGGCTTG

AAGAACGGGGATAACTGGGATGATAGCATTCAATCAAAGGAAAAAGTTGAAGCGAATTTG

CTGAGCAAGTACGAGGCAACACAGAGAAGGGAAAGGGCATTGGCTTATGCATACTCTCAT

CAGCAAAACTGGAAGAACAACTCTAAATCTGCTAACCCTATGTTCATGGATCCAAGCAAC

CCGACATGGGGTTGGAGCTGGTTAGAGAGATGGATGGCTGGTCGGCCATTAGAGAGTTCC

GAGAAAGAACAAAACAGCAGCAACAACAATGACAATGCTGCTTCAGTCAAGGGATCTATT

AACCGCAATAGCTCAACTCAACCAAACACACCATCGTCCGCAAGAGCCACCCCAAGAAAC

AAGAACAGTTTCTTCTCTCCTCCAACTCCCTCAAGGCTAAACCAATCCTCAAGAAAATCC

AACAACAATGATGACGACGCCAAAAGCACAATCTCGGTCCTGTCTGAGAGGAACCGTAGA

CACAGCATCGCTGGGTCATCAGTGAGAGACGACGAGAGCCTTGCTGGGTCACCAGCTCTC

CCAAGCTACATGGTTCCAACTAAATCAGCTAGAGCCAGGCTGAAGCCGCAGAGCCCAATA

GGTGGTACTACCCAAGAAAATGATGGGTTCACGGACAAGGCATCAGCTAAGAAACGGCTA

TCATATCCTACGTCACCTGCATTGCCTAAACCAAGGCGATTCTCAGCTCCGCCTAAGGTG

GAGCATAGCGGCGTCATCGTGACTAATGGAGGTGGCAGC

> T_Unigene_BMK.11540 gi|145357576|ref|NP_568110.2| 0 gi|145357576|ref|NP_568110.2| iqd2 (IQ-domain 2); calmodulin binding [Arabidopsis thaliana] AGTGGACTCCTCCAGGTTTTATTATCTTCAAGGTTTTTGAGCTCTTCGTCTATCAGATCTGGTGTCACTCTCTCTTACAGAATCAGATTAGAGATGGGCAAAAAAGGAAAATGGTTTTCAAGTGTTAAGAAAGCTTTCAGCCCAGATTCAAAGAAGTCGAAACAAAAATTGGCTGAGAGCCAAAATGGAGTGATCTCTAATGCTCCTTTGCCGGATAATGCCAGACAAGCTTCTCCTCCTGCTGCTCCTCCTCCTCTTGAGGTGAGAGTAGCTGAAGTGATTGTTGAACAGAACAGGAATATTTCTCCTCCTTCCACGGCAGATGTTGTGAATGTTACAGCCACTGATGTATCTGTAGTTCCATCTTCATCTACTCCTGAGGTTGTTCGTCGTGCTACAGCTACTCGATTTGCTGGAAAGTCAAACGAAGAAGCAGCTGCAATCTTGATTCAGACTATATTTAGAGGCTACTTGGCACGAAGAGCACTGAGGGCAATGAGGGGTTTGGTCAGACTTAAGTTATTGATGGAAGGATCTGTTGTTAAACGGCAAGCTGCAAATACTCTAAAATGTATGCAGACTCTCTCTCGTGTACAGTCACAGATCCGAGCTAGGAGAATCAGGATGTCAGAAGAGAATCAAGCTCGCCAGAAGCAACTCCTTCAGAAGCATGCCAAAGAGTTAGCTGGCTTGAAGAACGGGGATAACTGGGATGATAGCATTCAATCAAAGGAAAAAGTTGAAGCGAATTTGCTGAGCAAGTACGAGGCAACACAGAGAAGGGAAAGGGCATTGGCTTATGCATACTCTCATCAGCAAAACTGGAAGAACAACTCTAAATCTGCAAACCCGATGTTCATGGATCCGAGCAACCCGACATGGGGTTGGAGCTGGTTAGAGAGATGGATGGCTTGTCGGCCATTAGAGAGTTCCGAGAAAGAACAAAACAGCAGCAACAACAATGACAATGCTGCTTCAATCAAGGGATCTATTAACCGCAATAGCTCAACTCAACCAAACACACCATCGTCCGCAAGAGCCACCCCAAGAAACAAGAACAGTTTCTTCTCTCCTCCAACTCCCTCAAGGCTAAACCAATCCTCAAGAAAATCCAACAACAATGATGACGATGCCAAAAGCACAATCTCGGTCCTGTCTGAGAGGAACCGTAGACACAGCATCGCTGGGTCATCAGTGAGAGACGACGAGAGCCTTGCTGGGTCACCGGCTCTCCCGAGCTACATGGTTCCAACTAAATCAGCTAGAGCCAGGCTGAAGCCGCAGAGCCCAATAGGTGGTACCACGCAAGAAAATGATGGGTTCACGGACAAGGCATCAGCTAAAAAACGGCTCTCATATCCTACTTCACCTGCATTGCCTAAACCAAGGCGATTCTCAGCTCCGCCTAAGGTGGAGCATAGCGGCGTCATCGTGACCAATGGAGGAGGCAGC

**MYB28**

>CL622.Contig5_All 226 1332 myb domain protein 28 [Arabidopsis thaliana] >gi|9757864|dbj|BAB08498.1| transcription factor [Arabidopsis thaliana] >gi|41619486|gb|AAS10113.1| MYB transcription factor [Arabidopsis thaliana] >gi|114213503|gb|ABI54334.1| At5g61420 [Arabidopsis thaliana] >gi|332010084|gb|AED97467.1| myb domain protein 28 [Arabidopsis thaliana]

GTTGAACTCTACGTGAAAAAATGAAAACACGTAGCAGCGGTCTGGGTAAG

ATCCAAGAGCGTTTCTCCATCAGTCTCATATTCAGATGCATCAGAGTTCT

CATCAACAGATCTATTTCTTTCTTATTTGATTAAACAATTTTCCTTTCAA

AATTTGCTTCCAATTTTTGTGTTTCTCTGTTTGAAATCTTGAGTGATTGT

GAGAAGTTATACATCGGGGAAAAAAATGTCAAGAAAGCCATGTTGTGCCG

GAGAAGGGCTGAAGAAAGGGGCGTGGACCACCGAGGAGGACAAGAAACTC

ATCTCTTACATCCACGACCATGGCGAAGGAGGCTGGCGTGACATTCCCCA

AAAAGCTGGGTTGAAACGGTGTGGAAAGAGTTGTAGACTGAGATGGACTA

ATTACCTAAAACCTGAGATCAAAAGAGGCGAGTTTAGTTCAGAGGAGGAG

CAGATTATCATCATGCTTCATGCTTCTCGTGGCAACAAGTGGTCGGTCAT

AGCGAGACATTTACCTCGAAGAACAGACAACGAGATCAAGAATTACTGGA

ACACGCATCTCAAAAAACGTTTGATCGAACAGGGTATTGATCCCACGACT

CACAAGCCACTAGCTTCTAATTCCAACCCTACAGCTGCTGAGAATCTGAA

TCCCCTTATTAATGCCCCTAGTTCCGACAAGCCATACTCCAGGTCGAGCT

CAATGCCTTCTCTGTCTCCCTTCTCTGGTTTCAACACGACTTCCGAGGTG

TTCGAGATTAGCAACAATGATGGGAAACAAGTGGAGAGAGGTCCCTTGAG

TTGCAAGAAACGTTTTAAAAAATATAGTTCTACATCAAGGTTGCTGAACA

AAGTTGCAGCTAAGGCCACTTCCATCAAAGAAATATTGTCGGCTTCCATG

GAAGGTAGCTTGAGTGCTTCGACAATATCACATGCAAGCTTTTTCAATGG

CTTCTCTGAGCAGATTCTCAATGACGATGATAGTTCTAACGCAACCGTGA

CAAATACTCTGGCTGAATTCGACCCCTTCTCCCAATCATTGTACCCAGAG

CATGAGATCAATCCTACTTCTGATCTCGGCATGGACCAGAGTTACGATTT

CTCACATTTCCTCGAGAAGCTAAGGGGAGATAACCAGGATGAGGAAAACA

ATATGAATGTTGAATATAGTCACGATCTTCTTATGTCCGATGTTTCCCAA

GAAGTCTCATCAACTAGCGTTGATGATCAAGACAATATGGTGGGAAACTT

GGAGGGATGGTCCAAATATCTTCTTGACCATAATGATTTCATGTACGAGA

TCGACTCAGATTCTCTCGAAAAGCATTTCGTATGACCATTCATATCCCAA

GAGAGGTTTGAAATCATTTGAAGACGTAAAACAATGGTTATGTATCCAAG

GCTAGTCTATTACTACTAACTAGCTCGAACGAGATGTTGTGTATGTGTTT

AATTAGTATTTGGGTTGTTTAGTATCTGCTGCCCAAAGATTCGTGGTTAT

TAATCCCAAGTGTATGGTTAATACGACGTTAAATAAGGGTTTATCTTAGT

AAAAAATAGGTTTTGAGTAAGGGTTTCTCTTACATTGAGAACCACATGCA

TGTATATAACCTGGCGGATCAATTGGTAATTGATTTGCGCGGGCC

> CK_Unigene_BMK.23587 gi|297797099|ref|XP_002866434.1| 0 gi|297797099|ref|XP_002866434.1| hypothetical protein ARALYDRAFT_919385 [Arabidopsis lyrata subsp. lyrata]

GAAGTTATACATCGGGGAAAAAAAATGTCAAGAAAGCCATGTTGTGCCGGAGAAGGGCTGAAGAAAGGGGCGTGGACCACCGAGGAGGACAAGAAACTCATCTCTTACATCCACGACCATGGCGAAGGAGGCTGGCGTGACATTCCCCAAAAAGCTGGGTTGAAACGGTGTGGAAAGAGTTGTAGACTGAGATGGACTAATTACCTAAAACCTGAGATCAAAAGAGGCGAGTTTAGTTCAGAGGAGGAGCAGATTATCATCATGCTTCATGCTTCTCGTGGCAACAAGTGGTCGGTCATAGCGAGACATTTACCTCGAAGAACAGACAACGAGATCAAGAATTACTGGAACACGCATCTCAAAAAACGTTTGATCGAACAGGGTATTGATCCCACGACTCACAAGCCACTAGCTTCTAATTCCAACCCTACAGCTGCTGAGAATCTGAATCCCCTAAATGCCCCTAGTTCCGACAAGCCATACTCCAGGTCGAGCTCAATGCCTTCTCTGTCTCCCTTCTCTGGTTTCAACATGACTTCCGAGGTGTTCGAGATTAGCAACAATGATGGGAAACAAGTGCAGGGAGGTCCCTTGAGTTGCAAGAAACGTTTTAAAAAATCTAGTTCTACATCAAGGTTGCTGAACAAAGTTGCAGCTAAGGCCACTTCCATCAAAGAAATATTGTCGGCTTCCATGGAAGGTAGCTTGAGTGCTTCGACAATATCACATGCAAGCTTTTTCAATGGCTTCTCTGAGCAGATTCTCAATGAAGATGATAGTTGTAACGCAACCGTGACAAATACTCTGGCTGAATTCGACCCCTTCTCCCAATCATTGTACCCAGAGCATGAGATCAATCCTACTTCTGATCTCGGCATGGACCAGAGTTACGATTTCTCACATTTCCTCGAGAAGCTAAGGGGAGATAACCAGAATGAGGAAAACAATATGAATGTTGAATATAGTCACGATCTTCTTATGTCCGATGTTTCCCAAGAAGTCTCATCAACTAGCGTTGATGATCAAGACAATATGGTGGGAAACTTGGAGGGATGGTCCAAATATCTTCTTGACCATAATGATTTCATGTACGAGATCGACTCAGATTCTCTCGAAAAGCATTTCGTA

**MYB29**

>CL622.Contig4_All 187 1263 minus strand myb domain protein 29 [Arabidopsis thaliana] >gi|9759591|dbj|BAB11448.1| transcription factor-like protein [Arabidopsis thaliana] >gi|41619370|gb|AAS10087.1| MYB transcription factor [Arabidopsis thaliana] >gi|332003811|gb|AED91194.1| myb domain protein 29 [Arabidopsis thaliana]

ATGTCAAGAAAGCCATGTTGTGTGGGAGAAGGGCTGAAGAAAGGAGCATGGACCGCCGAA

GAAGACAAGAAACTCATCTCTTACATTCATGATCACGGCGAAGGAGGCTGGCGAGACATT

CCTCAAAAAGCTGGACTAAAACGATGTGGAAAGAGTTGTAGATTGCGATGGGCTAACTAT

TTGAAACCAGATATCAAGAGAGGAGAGTTTAGCTACGAAGAGGAACAAATTATCATCATG

CTTCACGCTTCTCGCGGAAACAAGTGGTCGGTCATAGCGAGACATTTGCCCAAAAGAACA

GACAATGAAATCAAGAACTACTGGAACACGCATCTCAAAAAACGCCTGATCGATCAGGGA

ATCGATCCCGTGACCCACAAGCCACTTGCCTCTAACCCTAATCCTAAGGCTTCTGATTTC

CAAGATGATGATGATGATGACTCAAACCAGGATGAGCAATCGCAATCAGATTCCATATCT

CCAAAGTCTCTTCCTCCTTCTTCTTCAAGCTACTGCAATCTACCGGAGATAAGCAGCAGC

ACAGATGAGACACCGATCAGAAACAATGTTTCCGTAAGCTCCAAGAAGCGTTATTTTAAG

AGATCGAGTTCTACATCAAAACTGTTAAACAAAGTTGCAGCTAGGGCTGCTTCCATTGCA

ACTATCTTATCAGCCTCCGTTGAAGGAACCTTGATGAGCTCTTCTACACCATTGTCCTCA

TCATGTCCCAATGATGACTTGTCGGAAACTAGTCCTTTTCAGATGGACGACTTTTATCCA

TTTTCTCAGTCGTCTGAACACAAGATTCTCAGTGACATCGGCATCAACGACACTGATCTC

AGCAATTCTGGATACAATTTCTCGCAGTTTCTTGAGAAATTAAGTAACGACGAAGGCGAA

GAAGCCGAGAACATTGGAGGAGGATATAATCAAGATCTGCTTATGTCTGATGTCTCATCA

ACAAGCGTTGATGACGATGATATTATGCGGAACATAACCGGTTGGTCAAATTATCTTACT

GACCATTCCGATTTCAATTATGACACGAATCAAGATTACGACGAGACGAACTTCATA

> T_Unigene_BMK.23671 gi|3941436|gb|AAC83594.1| 1.43222e-96 gi|3941436|gb|AAC83594.1| putative transcription factor [Arabidopsis thaliana]

GATGATGATGATGATGATGATGACTCAAACCAGGATGAGCAATCGCAATCAGATTCCATATCTCCAAAGTCTCTTCCTCCTTCTTCTTCAAGCTACTGCAATCTACCGGAGATAAGCAGCAGCACAGATGAGACACCGATCAGAAACAATGCTTCCTTAAGCTCCAATAAGCGTTATTTTAAGAGATCGAGTTCTACATCAAAACTGTTAAACAAAGTTGCAGCTAGGGCTGCTTCCATTGCAACTATCTTATCAGCCTCCGTTGAAGGAACCTTGATGAGCTCTTCTACACCATTGTCCTCATCATGTCCCAATGATGACTTGTCGGAAACTAGTCATTTTCAGATGGACGACTTTTATCCATTTTCTCAGTCGTCTGAACACAAGATTCTCAGTGATATCGGCATCAACGACACTGATCTCAGCAATTCTGAATACAATTTCTCGCAGTTTCTTGAGAAATTAAGTAACGACGAAGGCGAAGAAGCCGAGAACATTGGAGGAGGATATAATCAAGATCTGCTTATGTCTGATGTCTCATCAACAAGCGTTGATGACGATGATATTATGCGGAACATAACCGGTTGGTCAAATTATCTTACTGACCATTCCGATTTCAATTATGACACGAATCAAGATTACGACGAGACGAACTTCATA

> T_Unigene_BMK.23672 gi|312283111|dbj|BAJ34421.1| 2.36499e-99 gi|312283111|dbj|BAJ34421.1| unnamed protein product [Thellungiella halophila]

ATTCGACGACATTTCTGGGGTTTTTTTTCCCTTAATTATAATTTTCCTATTTCTTCTTCATCTGTATATGTAAATTTATATCTGAAAGAAACAAAACAAAAAATGTCAAGAAAGCCATGTTGTGTGGGAGAAGGGCTGAAGAAAGGAGCATGGACCGCCGAAGAAGACAAGAAACTCATCTCTTACATTCATGATCACGGCGAAGGAGGCTGGCGAGACATTCCTCAAAAAGCTGGACTAAAACGATGTGGAAAGAGTTGTAGATTGCGATGGGCTAACTATTTGAAACCAGATATCAAGAGAGGAGAGTTTAGCTACGAAGAGGAACAAATTATCATCATGCTTCACGCTTCTCGCGGAAACAAGTGGTCGGTCATAGCGAGACATTTGCCCAAAAGAACAGACAATGAAATCAAGAACTACTGGAACACGCATCTCAAAAAACGCCTGATCGATCAGGGAATCGATCCCGTGACCCACAAGCCACTTGCCTCTAACCCTAATCCTAAGGCTTCTGATTTCCAAGATGATGATGATGATGAT

**MYB34**

>CL6638.Contig1_All 300 1196 myb domain protein 34 [Arabidopsis thaliana] >gi|3150037|gb|AAC16897.1| ATR1 [Arabidopsis thaliana] >gi|10177313|dbj|BAB10639.1| ATR1 [Arabidopsis thaliana] >gi|41619482|gb|AAS10112.1| MYB transcription factor [Arabidopsis thaliana] >gi|126352288|gb|ABO09889.1| At5g60890 [Arabidopsis thaliana] >gi|332010010|gb|AED97393.1| myb domain protein 34 [Arabidopsis thaliana]

ATGGTGAGGACACCATGTTGCAAAGAAGAAGGAATAAAGAAAGGGGCTTGGACTCCTGAG

GAAGATCAAAAGCTTATTGCTTATCTTCAATTACATGGTGAAGGTGGATGGCGTACTCTC

CCTGAAAAAGCTGAGTTGAAGAGATGTGGGAAGAGTTGTCGATTGAGATGGGCGAATTAC

TTAAGACCAGATATTAAGAGAGGAGAGTTTAGTCGTGAAGAAGACGAAACTATCATCAAG

CTTCATGCTCTCAAGGGTAACAAGTGGGCCGCAATAGCCACTAGTTTGGCGGGACGAACT

GACAACGAAATAAAAAATTATTGGAACACCAATCTCAAGAAGCGTTTGAAACAAAAAGGC

CTCGATCCAACCACTCACAAACCGATCAATTCAACCGGTCTCGAACCGAAAAACAATAAA

CCCGTTAGTTCATCCGGTTCCGCGAGGCTTCTTAACCGCGTCGCAAGCAAATATGCCGTC

GAATTAAACCGGGATTTACTAACCGGAATCATCAGCGGAAGCTCCACAATCGTCGAGGAT

TCACAAAACTCCAGCGACGTTGAGTCTCCGACCTCCACATTTCTCAACAAAACGGCGGCA

GGATCAACCGGTCTCACATCGATTCTGATGGACACTACGTCGACATCTTCCGGCTTCTCC

GAAAACTGTTCTTTCTCCGATGGTTTGGCTGAATTCTTTAGCAACGAAGAGATCTCCGAT

ATGTATACGACAGTCGATAATTTTGGATTCATGGAGGAGCTAAAGGGTATTTTAAGCTAC

GACGGTTCCCACGCCGGAGTTATTGAAGATTCGCCGGAGGTTAATGTTATTACTGATGAA

ATGGAGTTTCTTGATTCTTGGAACGAAGAAGATCACAGTATGGTTGGAGTCTTTGTC

> T_Unigene_BMK.21595 gi|297793651|ref|XP_002864710.1| 0 gi|297793651|ref|XP_002864710.1| hypothetical protein ARALYDRAFT_496240 [Arabidopsis lyrata subsp. lyrata]

AATCAAGAATCAAGAATCAAGAAGGAGGAAAGAGATATGGTGAGGACACCATGTTGCAAAGAAGAAGGAATAAAGAAAGGGGCTTGGACACCTGAGGAAGATCAAAAGCTTATTGCTTATCTTCAATTACATGGTGAAGGTGGATGGCGTACTCTCCCTGAAAAAGCCGGGTTGAAGAGATGTGGGAAGAGTTGTAGATTGAGATGGGCGAATTACTTAAGACCAGATATTAAGAGAGGAGAGTTTAGTCGTGAAGAAGACGACACTATCATCAAGCTTCATGCTCTCAAGGGTAACAAGTGGGCCGCAATAGCCACTAGTTTGGCGGGACGAACTGACAACGAAATAAAAAATTATTGGAACACCAATCTCAAGAAGCGTTTGAAACAAAAAGGCCTCGATCCAATCACTCACAAACCGATCAATTCAACCGGTCTCGAACCGAAAAACAATAAACCCGTTAGTTCATCCGGTTCCGCAAGGCTTCTTAACCGCGTCGCTAGCAAATATGCCGTCGAATTAAACCGGGATTTACTAACCGGAATCATCAGCGGAAGCTCCACAATCGTCGAGGATTCACAAAACTCCAGCGACGTTGAGTCTCCGACCTCCACATTTCTCAACAAAACGGCGGCAGGATCAACCGGTCTCACATCGATTCTGATGGACACTACGTCGACATCTTCCGGCTTCTCCGAAAACTGTTCTTTCTCCGATGGTTTGGCTGAATTCTTTAGCAACGAAGAGATCTCCGATATGTATACGACAGTCGATCATTTTGGATTCATGGAGGAGCTAAAGGGTATTTTAAGCTACGACGGTTCCCACGCCGGAGTTATTGAAGATTCGCCGGAGGTTAATGTAATTACTGATGAAATGGAGTTTCTTGATTCTTGGAACGAAGAAGATCACAGTATGGTTGGAGTCTTTGTC

**MYB51**

>CL4908.Contig1_All 361 1413 myb domain protein 51 [Arabidopsis thaliana] >gi|30017319|gb|AAP12893.1| At1g18570 [Arabidopsis thaliana] >gi|41619108|gb|AAS10025.1| MYB transcription factor [Arabidopsis thaliana] >gi|332191609|gb|AEE29730.1| myb domain protein 51 [Arabidopsis thaliana]

ATGGTGCGGACACCCTGCTGCAAAGCTGAGCTAGGGTTAAAGAAAGGAGCTTGGACTCCC

GAAGAAGATCAGAAGCTTCTCTCTTACCTTAACCGTCACGGTGAAGGTGGATGGCGAACT

CTCCCCGAAAAAGCTGGACTCAAGAGATGCGGCAAAAGCTGCAGACTGAGATGGGCCAAT

TATCTTAGACCTGATATCAAAAGAGGAGAGTTCACCGAAGACGAAGAACGTTCTATCATC

TCTCTCCACGCCCTTCACGGCAACAAATGGTCTGCCATAGCTCGTGGATTACCAGGAAGA

ACCGATAACGAAATCAAGAACTACTGGAACACTCATATCAAAAAACGTTTGATCAAGAAA

GGTATCGATCCGGTTACACACAAGGCCTTGAGCTCAAAAAACCTCCCAGAGAAACAAAAC

GTTAATCATACGTTAACGAGTGGTGATGATCTCGATAATGAAAAGACGAAGAAGAACGAC

AAGAAGCCGGGATTCTCATCGGCTAGGTTCTTGAACAAAGTAGCTAATAGATTCGGAAAG

AGAATCAATCACAGTGTTCTATCTGAGATTATTGGAAGTGGTGGCCCACTTACTACTACC

ACTCACATTACTACTACTACTACAACAAGTGTTTCCGTTGACTCCGAATCAGATCAGTCA

ACGAGCTCCTCCCTAGCACCAACCTCGAACCTTCTCTGTCAAATTGCCCTTGCAACAACT

CCTGTTTCATTGATCTTTGACGTTAACGGTAACAGTAACGTTCATCCGACGACAACCTCT

TCGTCCACGTTCTCTGATGCCTCCTTTAACGATCCTCTAATGTACTGTGATAATTATTTC

GTTGGTAATAACATTGATGATGAGGATACTATCAGATTCTCGACATTTCTCAATGATGAA

GATTTCGTCATGTTGGAGGATTCTTGTGTTGGGAACACTGTGTTCATGAAAGAACTTACG

AGGTTTCTTCAAGAGGATGAAAACGACATCGCTGAGGTCACACCGGTCTATGAACGACAA

GACATTTTTGACGAGATCGATAACTATTTTGGA

> CK_Unigene_BMK.7289 gi|297844808|ref|XP_002890285.1| 0 gi|297844808|ref|XP_002890285.1| hypothetical protein ARALYDRAFT_472077 [Arabidopsis lyrata subsp. lyrata]

CAAGAATCAAGAATGGTGCGGACACCGTGCTGCAAAGCTGAGCTAGGGTTAAAGAAAGGAGCTTGGACTCCCGAAGAAGATCAGAAGCTTCTCTCTTACCTTAACCGTCACGGTGAAGGTGGATGGCGAACTCTCCCCGAAAAAGCTGGACTCAAGAGATGCGGCAAAAGCTGCAGACTAAGATGGGCCAATTATCTTAGACCTGATATCAAAAGAGGAGAGTTCACCGAAGACGAAGAACTTTCTATCATCTCCCTCCACGCCCTTCACGGCAACAAATGGTCTGCCATAGCTCGTGGATTACCAGGAAGAACCGATAACGAAATCAAGAACTACTGGAACACTCATATCAAAAAACGTTTGATCAAGAAAGGTATCGATCCGGTTACACACAAGGCCTTGAACTCAAAAAACATCCCAGAGAAACAAAACGTTAATCATACGTTAACGAGTGATGATGATCTCGATAATGAGAAGACGAAGAAGAACGACAAGAAGCCGGGATTCTCGTCGGCTAGGTTCTTGAACAAAGTAGCTAATAGATTCGGAAAGAGAATCAATCACAGTGTTCTATCTGAGATTATTGGAAGTGGTGGCCCACTTACTACTACCACTCACATTACTACTACTACGACTACAACAAGTGTTTCCGTTGACTCCGAATCAGATCAGTCAACGAGCTCCTCCCTAGCACCAACCTCGAACCTTCTCTGTCAAATGGCCGTTGCAAACGTTAACGGTAACAGTAACGTTCATCCGACGACAACCTCTTCGTCCACGTTCTCTGATGCCTCCTTTAACGATCCTCTAATGTACTGTGATAATTATTTCGTTGGTAATAACATTGATGATGAGGATACTATCAGATTCTCGACATTTCTCAATGATGAAGATTTCGTGATGTTGGAGGATTCTTGTGTTGAGAACACTGTGTTCATGAAAGAACTTACGAGGTTTCTTCAAGAGGATGAAAACGACATCGCTGAGGTCACACCGGTCTATGAACGACAAGACATTTTTGACGAGATCGATAACTATTTTGGA

**MYB76**

> T_Unigene_BMK.12366 gi|297806805|ref|XP_002871286.1| 0 gi|297806805|ref|XP_002871286.1| predicted protein [Arabidopsis lyrata subsp. lyrata]

TGGTTCTCAGTTAGTTTCCTATTCAGATGCATCATAATTCTATTCGAAGAAAATAATTCTATTCGAAGAGATATCTCTCTCTGTTATGAATTAGCTACTTCTTCCTTACGCACGTTTGTCCTATATATAAGGAAGAAAATGTCAAAGAAACCATGTTTTACTGGAGAAGGATTGAAGAAAGGAGCGTGGAGTGCAGAAGAGGATAAAAAACTCATCTCTTATATCCACGACCATGGCGAAGGAGGCTGGCGTGACATTCCCGCAAAAGCTGGTTTAAAACGGTGTGGAAAGAGTTGTAGATTGAGGTGGGCTAACTATTTAAAACCAGATATCAAGAGAGGAGAATTTAGCTACGAGGAAGAACAGATTATCATCATGCTTCATGCTTCTCGTGGCAATAAGTGGTCGGTCATTGCAAGACATTTGCCAAAAAGAACAGACAACGAGATCAAAAATTATTGGAACACACATCTCAAGAAACGTCTGATCGATGAAGGCATTGATCCCGTGACACACAAGCCACTAGCTTCTTCTAACCCTAAACCAACCGAGCCTAACAAGTTTTATTCCCAAGAAGGATCCAATCCGGATGAGCACTCGTCACAATGGAGTTCTACGTCTCCAATATCTCATCCCGTTTCTTCGAGTTTCAACAGTGCTATACCCAAAATCAGGAGTGACGAGACTGCGTTAGAGTATGGTTTCTTGAGCTGCAAGAAACGTTTGGAAAGATCGAGCTCTACTACATCAAAGCTGTTAAACAAAGTTGCAGCTAAAGCTTCTTCCATTGGGAAGATCTTATCAACCTCCATTGAAGGAACCTTGAGATCTCCTTCATCTTCTTCATGTCACCCAAACTCATTGTCTCAATCATCTGAACAAATGATCGATAACAAGGAAGATCTTAGTACGAGCATTGATCACAACATCCCCCCCGAGTATGATTTCTCAAACTTTCTCGAGCAACTCATTGACGACGACGAAGCCGAGAACGTTGGGGGCAACAATCAAGATCTCCTGATGTCCGATGTCCCATCAACATTAGTTGATGAAGACAATATGATTGGAGACATAACCGGTTGGTCAAGTTATATTCTTGACCATCCCAATTTTATGAATGAGTTCGAAGCGGAA
